# *Mycobacterium tuberculosis*-induced PCBP1 degradation drives macrophage ferroptosis to promote infection: a lung-macrophage-targeted RNAa nanotherapy

**DOI:** 10.1186/s12951-026-04569-x

**Published:** 2026-05-21

**Authors:** Huan-Shao Huang, Jia-Xin Chi, Jia-Jun Wang, Le-Yao Xiao, Lan Chen, Shi-Ying Lai, Wan-Yi Liu, Feng Yang, Kang-Sheng Liao, Jiang Pi, Yan-Guang Cong, Yi-Ming Shao, Jun-Fa Xu

**Affiliations:** 1 Dongguan Key Laboratory for Pathogenesis and Experimental Diagnosis of Infectious Diseases, The First Dongguan Affiliated Hospital, School of Medical Technology, Guangdong Medical University, Dongguan, 523710 Guangdong province China; 2 Institute of Laboratory Medicine, School of Medical Technology, Guangdong Medical University, Dongguan, 523808 Guangdong province China; 3 Songshan Lake Innovation Center of Medicine & Engineering, Guangdong Medical University, Dongguan, 523808 Guangdong province China

**Keywords:** *Mycobacterium tuberculosis*, PCBP1, RNA activation (RNAa), Nanotherapy, Ferroptosis, Host-directed therapy

## Abstract

**Background:**

Immune evasion by *Mycobacterium tuberculosis* (Mtb) complicates tuberculosis (TB) therapy. Ferroptosis, an iron-dependent form of regulated cell death, is increasingly recognized as a critical process in host-pathogen interactions. We aimed to define the role of poly(C)-binding protein 1 (PCBP1) in macrophage ferroptosis during Mtb infection and to develop a targeted RNA activation (RNAa) nanotherapy to exploit this pathway.

**Methods:**

We analyzed clinical samples from TB patients and investigated Mtb-host interactions in macrophage models using molecular and biochemical assays. Mannosylated lipid nanoparticles (MLNPs) were engineered to deliver PCBP1-targeting small activating RNAs (saRNAs). Therapeutic efficacy, lung-specific delivery, and biocompatibility were evaluated in a murine TB model.

**Results:**

Mtb utilizes the host E3 ubiquitin ligase Trim21 to mediate the proteasomal degradation of PCBP1. PCBP1 loss induced macrophage ferroptosis by modulating its downstream targets GPX4, PTGS2, and HMOX1, promoting bacterial survival. In vitro, saPCBP1@MLNPs restored PCBP1 expression, reversed ferroptosis markers (Fe²⁺, 4-HNE), and reduced Mtb burden. In murine models, the nanotherapy achieved lung-specific delivery, significantly attenuated lung pathology, and enhanced bacterial clearance.

**Conclusions:**

PCBP1 is a critical, druggable immune-metabolic checkpoint that governs macrophage ferroptosis in TB. Our targeted RNAa nanotherapy represents a promising host-directed strategy for Mtb infection, linking a key molecular mechanism to a translational therapeutic platform and offering a new approach for treating drug-resistant TB.

**Graphical abstract:**

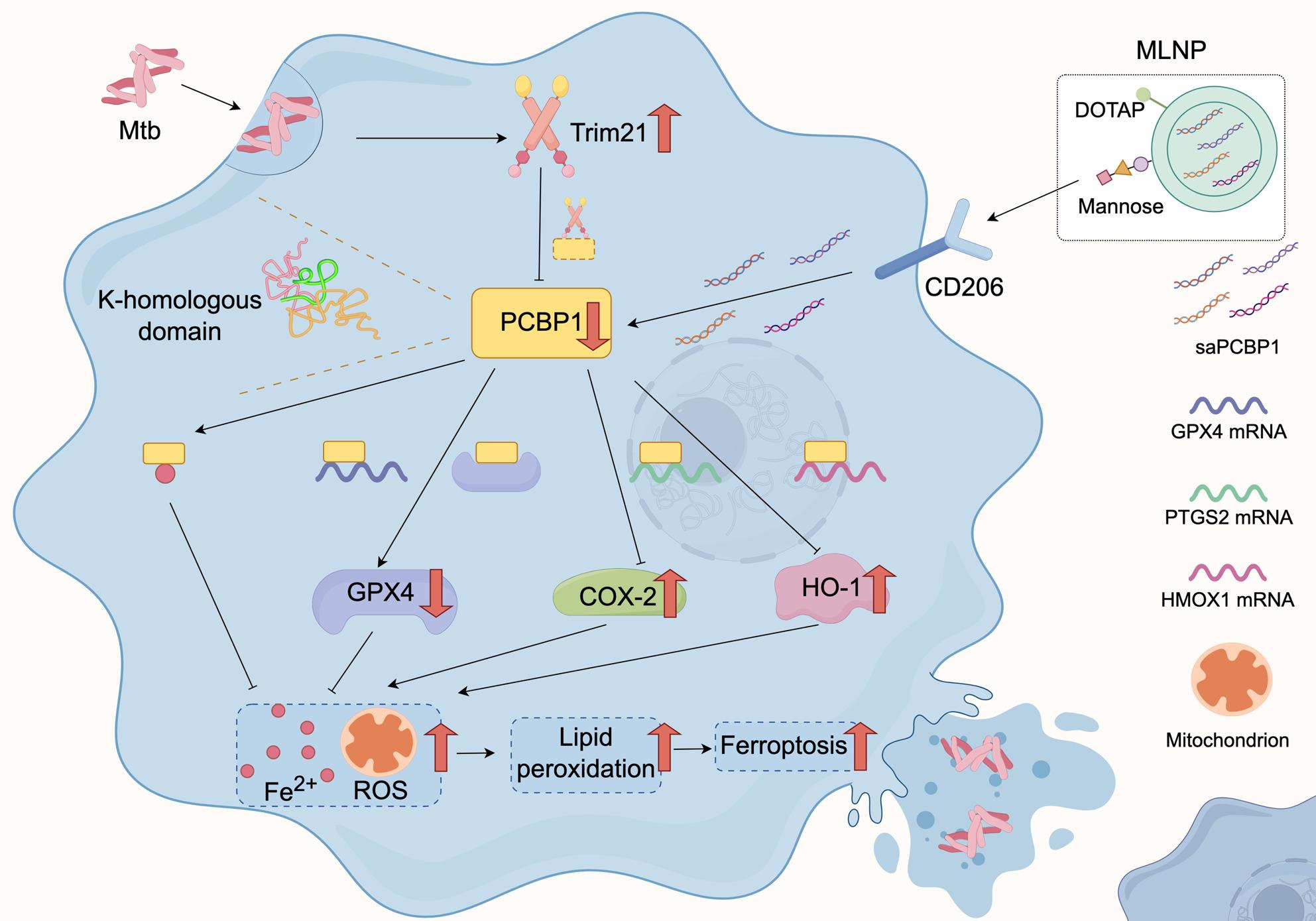

**Supplementary Information:**

The online version contains supplementary material available at 10.1186/s12951-026-04569-x.

## Background

Tuberculosis (TB), caused by *Mycobacterium tuberculosis* (Mtb), remains a formidable global health challenge. As an intracellular pathogen, Mtb deploys multifaceted strategies to subvert host immunity, including the blockade of phagosome-lysosome fusion, subversion of redox homeostasis, and exploitation of regulated cell death pathways [1–3]. Despite diagnostic advancements, the World Health Organization (WHO) reports about 10 million new TB cases and 1.3 million related deaths annually, with multidrug-resistant TB accounting for 13% of infections [4]. Current therapies are hindered by prolonged treatment durations (6–9 months), hepatotoxicity, and Mtb adaptive evolution through genomic mutations, which lead to drug resistance and increased treatment failure risks [5, 6]. Consequently, in-depth research into TB pathogenesis and the exploration of novel therapies have become a priority in the global medical community.

Macrophages, the primary battleground of Mtb infection, perform their antimicrobial functions using phagosome acidification and reactive species production [7, 8]. However, Mtb sabotages these defenses through secreted effectors that destabilize phagosomal integrity and manipulate autophagy-apoptosis cross-talk, converting macrophages into sheltered replicative niches [9, 10]. This immune evasion facilitates pathogen persistence and promotes dissemination through regulated cell death [1, 11, 12]. Emerging evidence suggests that ferroptosis, an iron-dependent cell death pathway driven by lipid peroxidation [13, 14], may play a role in TB pathogenesis. Indeed, Mtb can modulate host iron metabolism to enhance its intracellular survival [13–15]. Mtb disrupts iron metabolism to elevate labile iron pools, thereby activating lipid peroxidation cascades that culminate in ferroptosis. The resultant release of inflammatory mediators exacerbates tissue damage and granuloma formation [16, 17], yet the molecular orchestrators linking Mtb to ferroptotic signaling remain elusive. Given its well-known roles in iron homeostasis and ferroptosis regulation in various biological contexts, poly(C)-binding protein 1 (PCBP1) emerged as a potential candidate as an orchestrator [18, 19].

PCBP1 is a multifunctional RNA/DNA-binding protein involved in post-transcriptional regulation, iron homeostasis, and oxidative stress response [18, 19]. Located on human chromosome 2p13.3, the PCBP1 gene encodes a protein with multiple KH domains that enable RNA and DNA binding. PCBP1 regulates mRNA stability, translation efficiency, and alternative splicing through these domains [20, 21]. As an iron chaperone, PCBP1 transports free iron to ferritin and iron-sulfur cluster synthase, maintaining iron homeostasis and preventing or alleviating oxidative stress [22]. Several studies have shown that PCBP1 can effectively inhibit ferroptosis. For instance, it suppresses iron autophagy-related ferroptosis in head and neck cancer [23], protects mouse liver from lipid peroxidation [24], and safeguards bladder cancer cells from mitochondrial damage and ferroptosis [25]. Conversely, PCBP1 deficiency leads to intracellular free iron overload, activates lipid peroxidation pathways, and ultimately triggers ferroptosis [26]. Mtb exploits host iron via siderophores (e.g., mycobactin) to acquire iron from ferritin [27]. Hence, PCBP1 is a promising therapeutic target for Mtb infection.

The rapid advancement of RNA-based therapeutics, particularly mRNA vaccines, has established them as the third-generation drugs after small molecules and antibodies, leveraging high specificity, durability, and scalable production [28]. Small activating RNA (saRNA) offers a precision therapeutic strategy for loss-of-function diseases by recruiting RNA polymerase to the promoter region of target genes to activate transcription. For instance, saRNA targeting the miR-181a promoter can inhibit the proliferation of leukemia cells [29], while saRNA targeting MAS1 demonstrates broad-spectrum anticancer potential by modulating the renin-angiotensin system [30]. In the field of metabolic diseases, SIRT1-activating saRNA can reverse lipid metabolism disorders [31], and p21 saRNA can suppress proliferative vitreoretinopathy [32]. Clinically, MTL-CEBPA has entered trials, while RAG-01 achieved FDA Fast Track status in 2024, accelerating saRNA translation from bench to bedside [33]. Therefore, saRNA could be used to re-establish the expression and function of PCBP1 in infected macrophages by inhibiting ferroptosis, possibly reinforcing host defenses against Mtb.

Despite advancements in RNA technologies, nucleic acid drug delivery remains hindered by enzymatic degradation, poor membrane permeability, and endosomal escape limitations [34]. Lipid nanoparticles (LNPs) have emerged as one of the most promising delivery platforms. Their clinical value was confirmed by the success of COVID-19 mRNA vaccines [35–37]. Based on the parasitic and reproductive characteristics of Mtb within macrophages [38, 39], macrophages have long been recognized as critical cellular targets for TB therapy. Consequently, achieving macrophage-specific targeting of LNPs is paramount. Surface modifications of LNPs, such as PEGylation, can extend their systemic circulation time while enabling macrophage-specific targeting through ligand-receptor interactions [40, 41]. Building on our prior mannosylated nanocarrier for enhanced intracellular drug retention [42], we propose a “DOTAP-mannose dual-functionalized LNP” system: DOTAP concentrates LNPs in lung tissue [43], while mannose directs alveolar macrophage targeting. The strategy could synergistically overcome TB therapy barriers.

Therefore, this study investigated whether PCBP1 modulates macrophage iron metabolism and ferroptosis to restrict Mtb survival, hypothesizing its protective role in maintaining iron homeostasis and counteracting pathogen-induced cell death. In addition, we unveil a novel tripartite mechanism by which PCBP1 safeguards macrophages against ferroptosis: stabilizing GPX4, repressing PTGS2, and degrading HMOX1. By engineering mannosylated LNPs delivering PCBP1-targeting saRNAs, we can rescue iron homeostasis, quench lipid peroxidation, and enhance bacterial clearance in vivo. This work bridges the mechanistic discovery of PCBP1’s immunometabolic roles with translational saRNA nanotherapy, offering a strategy to circumvent drug resistance in TB.

## Methods

### Study subjects and ethics approval

A prospective cohort of 64 treatment-naive pulmonary TB patients (diagnosed per WHO guidelines with microbiological confirmation) and 63 age-/sex-matched healthy controls were enrolled at the Sixth People’s Hospital of Dongguan (Institutional Review Board (IRB) of Affiliated Hospital of Guangdong Medical University; ethical approval: YJYS2022039). The inclusion criteria were (1) met the WHO diagnostic criteria for TB and confirmed by microbiological examination, including positive sputum smear, positive sputum culture, or positive nucleic acid test (e.g., Xpert MTB/RIF), (2) age between 18 and 65 years, (3) never received tuberculosis treatment or received anti-tuberculosis treatment for less than 1 week, and (4) voluntary and able to provide written informed consent. The exclusion criteria were (1) suffering from other severe acute or chronic respiratory diseases (e.g., chronic obstructive pulmonary disease, lung cancer, interstitial lung disease, etc.), (2) severe liver or kidney dysfunction, (3) immunocompromised (e.g., HIV infection, receiving immunosuppressive treatment, etc.), (4) pregnant or lactating women, (5) unable to comply with study procedures or follow-up. All participants provided written informed consent under protocols approved by the institutional review boards of Guangdong Medical University and the Sixth People’s Hospital. Diagnostic verification followed Chinese TB diagnostic standards WS 196–2017 and WS 288–2017. Key clinical characteristics of the TB patient and healthy control cohorts are presented in Table 1.

**Table 1:** Comprehensive baseline characteristics of healthy controls and TB patients in the overall cohort

	HC group (*n* = 63)	TB group (*n* = 64)	*P* value
Age in years, Median (Q1, Q3)	36.22 (24.00, 46.00)	30.78 (25.25, 36.00)	0.077
Male sex, no. (%)	39 (61.9)	40 (62.5)	0.945
Sputum smear positive, n (%)	NA	31 (48.4)	NA
Sputum culture or NAAT positivity rate, n (%)	NA	58 (92.1)	NA
Bilateral prominent lung markings, n (%)	0 (0.0)	61 (95.3)	< 0.001
Comorbidities, n (%)	0 (0.0)	47 (73.4)	< 0.001
WBC (×10^9^/L), Median (Q1, Q3)	6.77 (5.41, 7.57)	9.54 (5.54, 13.56)	0.045
NEU (×10^9^/L), Median (Q1, Q3)	3.94 (2.83, 4.32)	6.82 (3.07,9.07)	0.003
LYM (×10^9^/L), means ± SD	2.21 ± 0.078	1.50 ± 0.081	< 0.001
MONO (×10^9^/L), Median (Q1, Q3)	0.40 (0.29, 0.49)	0.60 (0.38, 0.81)	< 0.001
NEU%, means ± SD	57.06 ± 1.196	68.45 ± 1.634	< 0.001
LYM%, Median (Q1, Q3)	33.63 (27.20, 39.10)	20.86 (10.30, 30.15)	< 0.001
MONO%, Median (Q1, Q3)	6.05 (4.80, 6.60)	6.98 (5.60, 8.00)	0.003
Hb (g/L), Median (Q1, Q3)	138.78 (128.00, 150.00)	115.23 (82.50, 141.00)	< 0.001
CRP (mg/L), Median (Q1, Q3)	0.91 (0.50, 1.26)	60.39 (0.58, 92.26)	< 0.001
AST (U/L), Median (Q1, Q3)	21.2 (18.60, 29.00)	30.6 (20.47, 30.90)	0.026
ALT (U/L), Median (Q1, Q3)	19.25 (11.00, 33.50)	27 (11.48, 31.00)	0.408
AST/ALT, Median (Q1, Q3)	1.89 (0.69, 2.17)	2.05 (1.31, 2.52)	0.025

Q1, First Quartile; Q3, Third Quartile; NA: Not available

SPF-grade male or female C57BL/6J mice (6–8 weeks old, 16–18 g) were provided by the Southern Medical University Laboratory Animal Center and housed in SPF facilities meeting national standards (Guangdong Medical University Experimental Animal Ethics Committee; ethical approval: GDY2202180). The specific housing conditions were a temperature of 20–26℃, relative humidity of 40%−60%, and a 12-h light/dark cycle was implemented. Mice had free access to sterilized food and water, and bedding was changed weekly to ensure a clean and stable living environment.

### Cell culture and preparation

Human primary macrophages were differentiated from peripheral blood mononuclear cells (PBMCs) obtained from healthy volunteers (aged 20–50 years). Donors were confirmed to be free of infectious diseases, immune disorders, and malignancies, and had not received recent immunomodulatory therapy. The study was approved by the Institutional Ethics Committee of Guangdong Medical University (approval: YJYS2022039), and written informed consent was obtained from all participants. PBMCs were isolated via Ficoll density gradient centrifugation and resuspended in RPMI 1640 medium supplemented with 10% fetal bovine serum (FBS) and 1% penicillin-streptomycin (referred to as complete medium). Cells were seeded in 12-well plates at a density of 1 × 10⁶ cells/mL. To induce differentiation into macrophages, recombinant human GM-CSF (40 ng/mL; Cat# 216-MC-025, R&D Systems) was added to the culture. The medium was replenished with fresh GM-CSF every 2 days. Differentiated macrophages displaying characteristic morphology were harvested on day 7 for subsequent experiments.

The THP-1 and RAW264.7 cell lines were purchased from Shanghai Gainney Co. and cultured in complete medium at 37 °C in a humidified 5% CO₂ atmosphere. THP-1 cells (passages 3–20) and RAW264.7 cells (passages 10–20) were maintained according to standard protocols. For differentiation, THP-1 cells were seeded at 1 × 10⁶ cells/mL and treated with 50 ng/mL phorbol 12-myristate 13-acetate (PMA; Cat# P8139, Sigma) for 24 h. Following incubation, non-adherent cells and residual PMA were removed by washing with PBS, and adherent macrophages were rested in fresh complete medium prior to infection.

### Mycobacterium strains and infection models

*Mycobacterium tuberculosis* strains H37Ra (ATCC 25617) and H37Rv (ATCC 27294) were obtained from Shanghai Pulmonary Hospital. Bacteria were cultured in Middlebrook 7H9 broth supplemented with oleic acid-albumin-dextrose-catalase (OADC) enrichment at 37 °C with shaking at 200 rpm. Cultures were grown to mid-log phase (OD₆₀₀ 0.8–1.0). For infection experiments, bacterial suspensions were washed with sterile saline and resuspended in PBS. The concentration was adjusted based on optical density measurements. All experiments involving *M. tuberculosis* were conducted in biosafety level 3 (BSL-3) facilities following approved biosafety and ethical guidelines.

For in vitro macrophage infection, cells were infected with virulent H37Rv at a multiplicity of infection (MOI) of 2 [44–46] or attenuated H37Ra at an MOI of 10 [47, 48]. The higher MOI for H37Ra was selected based on optimization experiments (Supplementary Fig. 2) to ensure sufficient intracellular bacterial burden and induction of ferroptosis phenotypes comparable to the virulent strain. After 6 h of infection, cells were washed three times with PBS to remove extracellular bacteria and incubated in fresh medium containing 10% FBS for the indicated time points.

For in vivo studies, C57BL/6J mice were housed under specific pathogen-free conditions. For the H37Rv infection model, mice were anesthetized with diethyl ether and infected intranasally with 10⁶ CFU of bacteria in 50 µL of suspension. For the H37Ra infection model, mice were anesthetized via intraperitoneal injection of sodium pentobarbital (150 µL/10 g body weight). The trachea was surgically exposed, and 2.5 × 10⁵ CFU of H37Ra (50 µL) was administered via intratracheal instillation. Post-infection, animals were monitored for recovery and returned to housing. Physiological parameters including body weight and general health status were monitored on days 1, 7, 14, and 21 post-infection.

### siRNA Transfection

For transfection, cells were plated at appropriate densities. Lipofectamine^™^ 3000 (Thermo Fisher, catalog: L3000008) and Opti-MEM I (Thermo Fisher, catalog: 31985062) were used to prepare transfection complexes, which were incubated and added to cells. After 12 h, cells were washed and cultured in complete medium for 48 h post-transfection. The siRNA sequences used in this study are listed in Table 2. The final concentration of siRNA was 1 µg/mL.

**Table 2:** siRNA sequences

Name	Sequence (5’→ 3’)
siNC	sense: UUCUCCGAACGUGUCACGUTT antisense: ACGUGACACGUUCGGAGAATT
Human-siPCBP1-1	sense: GAAGGTCGGAGTCAACGGATT antisense: UAAGUUAUUUGGAAUGGUGAG
Human-siPCBP1-2	sense: GAACCAGGUGGCAAGACAATT antisense: UUGUCUUGCCACCUGGUUCAG
Human-siTrim21-4	sense: UCAUUGUCAAGCGUGCUGCTT antisense: GCAGCACGCUUGACAAUGATT
Human-siTrim21-5	sense: GCAGCACGCUUGACAAUGATT antisense: UCAUUGUCAAGCGUGCUGCTT
Mouse-siPCBP1-1	sense: AAAGAUGGCAUUGGUAGGCCCAGUC antisense: GACUGGGCCUACCAAUGCCAUCUUU
Mouse-siPCBP1-2	sense: GCUCCAUGACCAACAGUAC antisense: GUACUGUUGGUCAUGGAGC
Mouse-siTrim21-1	sense: GGAGCCUAUGAGUAUCGAATT antisense: UUCGAUACUCAUAGGCUCCTT
Mouse-siTrim21-2	sense: CCAAUAGACAUAUAGCCAATT antisense: UUGUUAGGUGAGAAGUGGGTT

For THP-1-derived macrophages, the seeding density was 1 × 10⁶ cells/ml. For RAW264.7 macrophages, the seeding density was 5 × 10⁵ cells/ml. Cell suspensions were seeded into 12-well or 6-well plates, respectively, and cultured overnight in a 37℃, 5% CO₂ incubator to allow cells to adhere and reach a suitable growth state, ready for transfection experiments.

For THP-1-derived macrophages, it was ensured that cell confluence reached 70%−90%, while for RAW264.7 macrophages, cell confluence was controlled at 30%−50%. The day before transfection, the cells were seeded into appropriate culture vessels, ensuring good cell growth state and viability > 90%. Lipofectamine^™^ 3000 transfection reagent (Thermo, Catalog No.: L3000008) and Opti-MEM I Reduced Serum Medium (Thermo, Catalog No.: 31985062) were used. For 12-well plates, under sterile conditions, two sets of mixtures were prepared separately (mixture 1: 50 µL Opti-MEM I medium and 3 µL Lipofectamine^™^ 3000 reagent to a 1.5 mL centrifuge tube; Mixture 2: 50 µL Opti-MEM I medium and 3 µL siRNA to another centrifuge tube). The mixtures were incubated at room temperature for 5 min. Subsequently, Mixture 2 was added to Mixture 1 and mixed gently. They were incubated at room temperature for 15 min to allow complex formation. The prepared Lipofectamine-siRNA complex (100 µL) was added to the cells to be transfected, and the culture vessel was gently rocked to ensure even distribution of the complex. The cells were incubated in a 37℃, 5% CO₂ incubator for 12 h. After incubation, the cells were washed three times with PBS to remove unbound complexes. Culture was continued in complete medium for up to 48 h for subsequent experiments.

### Adenovirus construction and infection

In this study, the adenovirus vector based on human adenovirus type 5 (Ad5) was constructed, packaged, and quality-controlled by Hanheng Biotechnology (Shanghai). With features like wide infection range and high efficiency, four adenoviruses were used: HBAD-EGFP (overexpression control), HBAD-Adeasy-h-PCBP1-3xflag-EGFP, HBAD-Adeasy-h-PCBP1ΔRNA-3xflag-EGFP (ΔRNA: R40A/R124A/R306A), and HBAD-Adeasy-h-PCBP1ΔFe-3xflag-EGFP (ΔFe: D82A/E168A/E350A). THP-1-derived macrophages were infected with adenoviruses at an MOI of 1000. Infection efficiency was validated by fluorescence microscopy and Western blot (WB).

The titers of the adenoviruses used in this study were as follows: HBAD-EGFP at 4.0 × 10¹⁰ PFU/ml; HBAD-Adeasy-h-PCBP1-3xflag-EGFP at 1.58 × 10¹⁰ PFU/ml; HBAD-Adeasy-h-PCBP1ΔRNA-3xflag-EGFP (ΔRNA: R40A/R124A/R306A) at 2.51 × 10¹⁰ PFU/ml; and HBAD-Adeasy-h-PCBP1ΔFe-3xflag-EGFP (ΔFe: D82A/E168A/E350A) at 1.26 × 10¹⁰ PFU/ml.

The adenovirus infection was performed on THP-1-derived macrophages. Before infection, THP-1 cells had been differentiated into macrophages via PMA induction and cultured stably for 24 h to ensure cell state stability and macrophage characteristics and function.

The determination of MOI = 1000 was based on preliminary optimization experiments. Small-scale infection experiments were conducted under different MOI conditions (100, 500, 1000, 2000). Transduction efficiency and cytotoxicity were evaluated by observing GFP expression via fluorescence microscopy and detecting PCBP1 protein expression levels via Western blot. The experimental results showed that at MOI = 1000, the adenovirus achieved efficient gene transduction while cell viability remained high, with no obvious cytotoxicity or adverse reactions. Therefore, MOI = 1000 was chosen for subsequent adenovirus infection experiments.

The day before infection, THP-1 cells were seeded into 12-well plates at an appropriate density, approximately 1 × 10⁶ cells per well, and 1 ml of complete medium containing PMA (with 10% FBS) was added, so that cells reached approximately 70% − 80% confluence at the time of infection, ensuring good cell state. On the day of infection, an appropriate amount of virus stock was taken from −80℃ storage and diluted to the desired MOI = 1000 concentration using complete RPMI 1640 medium. Add 1 ml of the diluted adenovirus solution to each well, and gently shake the culture plate to distribute the virus solution evenly over the cell surface. The culture plates were placed in a 37℃, 5% CO₂ incubator for incubation. At 24 h post-infection, the medium was changed to fresh complete RPMI 1640 medium (without antibiotics) to remove unadsorbed virus particles and cell debris, reduce potential cytotoxicity, and promote cell recovery and stable gene expression. Subsequent experiments, such as Western blot, RT-PCR, and immunofluorescence, were mainly performed at 48 h post-infection.

### RNA extraction and RT-qPCR

Total RNA was isolated using TRIzol reagent (Invitrogen), and reverse transcription was performed using the PrimeScript RT Reagent Kit (Takara). Quantitative reverse transcription-polymerase chain reaction (RT-qPCR) was then performed using the SYBR PrimeScript RT-PCR Kit (Takara) to analyze gene expression. The 2^−△△CT^ method was employed for quantification, with GAPDH used as an internal control. The PCR primer pairs were synthesized by Sangon Biotech in Shanghai, China (sequences provided in Tables 3 and 4).

**Table 3:** Forward and reverse primer sequences of human and mouse for RT-qPCR

Name	Sequence (5’→ 3’)
Human-GAPDH	F: GAAGGTCGGAGTCAACGGATT R: CCTGGAAGATGGTGATGGGATT
Human-PCBP1	F: CTGGCGCTCAGCCATACAG R: CGCACTTATACTGGTCAAATCCC
Human-Trim21	F: CCATGTGCCAGGGCTGAAGAAG R: AGGTATGCTCTGCTGGGTGTCTC
Human-PTGS2	F: GTTCCACCCGCAGTACAGAA R: AGGGCTTCAGCATAAAGCGT
Human-GPX4	F: GCCTTCCCGTGTAACCAGT R: GCGAACTCTTTGATCTCTTCGT
Human-HMOX-1	F: AAGACTGCGTTCCTGCTCAAC R: AAAGCCCTACAGCAACTGTCG
Human-FTH1	F: TCCTACGTTTACCTGTCCATGT R: GTTTGTGCAGTTCCAGTAGTGA
Human-SRC	F: GAGCGGCTCCAGATTGTCAA R: CTGGGGATGTAGCCTGTCTGT
Human-VEGFA	F: AGGGCAGAATCATCACGAAGT R: AGGGTCTCGATTGGATGGCA
Human-DAZAP1	F: AGAAGTTCGGAGTGGTCACG R: ACTGATTGTTCGTCCTCGAAAG
Human-SLC3A2	F: TGGGTTCCAGGTTCGGGACATAG R: TCTGCTGAAGGTCGGAGGAGTTAG
Human-CXCL2	F: CCCAAACCGAAGTCATGCCACA R: GATTTTCTTAACCATGGGCGATGCG
Mouse-GAPDH	F: AGGTCGGTGTGAACGGATTTG R: TGTAGACCATGTAGTTGAGGTCA
Mouse-PCBP1	F: GACGCCGGTGTGACTGAAA R: GTCAGCGTGATGATCCTCTCC
Mouse-Trim21	F: GGAGAAGCCTAGTCCCCTG R: CCGTGGGCATAGGAAGAGT
Mouse-GPX4	F: TGTGCATCCCGCGATGATT R: CCCTGTACTTATCCAGGCAGA

F, Forward Sequence; R, Reverse Sequence

**Table 4:** Primer sequences for RNA immunoprecipitation (RIP)

Name	Sequence (5’→ 3’)
Human-GPX4(RIP)	F: CCCGATACGCTGAGTGTGGTTTG
	R: TCTTCGTTACTCCCTGGCTCCTG
Human-PTGS2(RIP)	F: ACTGCAGGCCTGGTACTCAG R: ACTTTTGCAATGTGATATGGACTGC
Human-HMOX1(RIP)	F: ACAAGGAGAGCCCAGTCTTCGC R: TGCTCCAGGGCAGCCTTGC
Human-SLC3A2(RIP)	F: ACTCCTCCGACCTTCAGCAGATC
	R: TGCTCCCCAGTAGAACCAGAATCAG
Human-FTH1(RIP)	F: GTACCCTGAGAATGCTCCCTCCTAG
	R: GATCACACAGGCTGGCAGTCTTG
Human-CXCL2(RIP)	F: CCTGCAGGGAATTCACCTCAA
	R: TATGACTTCGGTTTGGGCGC

For cell samples, the starting material quantity for each RNA extraction was 1 × 10⁶ cells. For mouse tissue samples, the starting material quantity for each RNA extraction was approximately 50 mg of tissue.

The template amount for reverse transcription was 500 ng of total RNA, which was used as template per reaction system (20 µl). The template amount for qPCR reaction was 2 µl of 10-fold diluted cDNA, which was used as template per qPCR system (10 µl). Amplification was performed using a two-step program: Pre-denaturation (1 cycle) at 95℃ for 30 s → PCR reaction (40 cycles): 95℃ for 5 s; 60℃ for 30 s → Dissociation curve analysis. To ensure that the primer amplification efficiency met the requirements for the 2-△△CT method, we performed standard curve analysis for all primer pairs. Using 5 serial dilutions of cDNA with known concentrations, three technical replicates were performed for each dilution, and then qPCR amplification was conducted under standard conditions. By analyzing the amplification curves and Ct values, standard curves were generated. Amplification efficiency was calculated using the formula [10^(−1/slope) − 1] × 100. The ideal range is 90% − 110%, and R² > 0.98. All primers passed this validation, ensuring the reliability of the experimental results.

### WB analysis

The starting material amount for each protein extraction was 1–2 × 10⁶ cells. For mouse tissue samples, approximately 100 mg of tissue was used for protein extraction. PMSF (MCE, catalog: HY-B0496) and phosphatase inhibitors (Applygen, catalog: P1260) were added to the RIPA buffer used. Protein concentration was quantified using the BCA Protein Assay Kit (Solarbio, catalog: PC0020) to ensure that the loading amount per lane was normalized by protein concentration. The loading amount per lane was 10–30 µg of total protein. SDS-PAGE was performed on a 10% polyacrylamide gel. Electrophoresis conditions were a constant voltage of 80 V during stacking for 30 min, followed by a constant voltage of 120 V until the bromophenol blue dye front reached the bottom of the gel. Transfer was performed using a PVDF membrane (Millipore, catalog: ISEQ00010) at a constant current of 250 mA for 90 min. Then, 5% non-fat dry milk (Solarbio, catalog: D8340) dissolved in TBST (Tris Buffered Saline with Tween-20) was used as the blocking solution, incubating at room temperature for 1 h. The antibodies: anti-PCBP1 (1:1000, MBL, RN024P), anti-GPX4 (1:1000, abcam, ab125066), anti-COX-2 (1:2000, Proteintech, 27308-1-AP), anti-HMOX-1 (1:1000, abcam, ab52947), anti-4-HNE (1:1000, Sigma-Aldrich, AB5605), anti-Tubulin (1:5000, Proteintech, 11224-1-AP), anti-β-actin (1:5000, Proteintech, 66009-1-Ig) and anti-GAPDH (1:5000, Proteintech, 60004-1-Ig). The secondary antibodies used were Goat Anti-Rabbit IgG H&L (HRP) (abcam, catalog: ab6721), Goat Anti-Mouse IgG H&L (HRP) (abcam, catalog: ab205719), and Donkey Anti-Goat IgG H&L (HRP) (abcam, catalog: ab6885). After primary antibody incubation, the membranes were washed with TBST 3 times, 10 min each time, with gentle shaking to remove unbound primary antibodies. All secondary antibodies were diluted at 1:10,000. After secondary antibody incubation, the membranes were washed with TBST 3 times, 10 min each time, to remove unbound secondary antibodies. This was followed by ECL detection. The ImageJ software version used was ImageJ 1.53t (NIH, USA, https://imagej.nih.gov/ij/).

### Co-immunoprecipitation (Co-IP)

In the Co-IP experiment, the starting cells were THP-1-derived macrophages, using 1 × 10⁸ cells per experimental group. The components of the IP buffer were 20 mM Tris (pH 7.4), 150 mM NaCl, 1 mM EDTA, 1 mM EGTA, 1% Triton X-100, and 1 mM β-glycerophosphate. PMSF (MCE, catalog: HY-B0496) and phosphatase inhibitors (Applygen, catalog: P1260) were added to the Co-IP buffer a few minutes before cell lysis to prevent protein degradation and dephosphorylation. In the Co-IP experiment, the anti-PCBP1 antibody (MBL, catalog: RN024P) and the anti-GPX4 antibody (antibodies-online, catalog: ABIN3015729) were both used at 5 µg/reaction. The magnetic bead type was Protein A/G magnetic beads (Thermo Fisher, catalog: 88802), used at 50 µl/reaction. The composition of the washing buffer was 50 mM Tris-HCl (pH 7.4), 150 mM NaCl, 1% NP-40, and 0.5% Na-deoxycholate, with the addition of protease inhibitors and phosphatase inhibitors. The washing details were as follows: 200 µL of washing buffer was added to gently resuspend the antigen-bead-antibody complex, then the mixture was placed on the magnetic separation rack and centrifuged to remove the supernatant. Repeat this washing step 6 times, removing the supernatant after each wash. For the final wash, the antigen-bead-antibody complex was gently resuspended in 100 µL of washing buffer and transferred to a new pre-cooled EP tube. The components of the Elution buffer were 50 mM Tris-HCl (pH 6.8) and 1% SDS. The EP tube was placed on the magnetic separation rack, and the supernatant was removed. Then, 20 µL of elution buffer and 10 µL of protein loading buffer were added to gently resuspend the complex. The EP tube was placed in a boiling water bath and boiled for 5 min to elute the antigen from the bead-antibody complex. The eluted sample was stored in a −20℃ or −80℃ freezer for subsequent detection, such as WB.

### RNA immunoprecipitation (RIP)

- RIP assays were performed using THP-1-derived macrophages. For each experimental group, 1 × 10⁸ cells were used. RIP experiments were conducted using the EZ-Magna RIP Kit (Merck Millipore, Cat. No. 17–701) according to the manufacturer’s instructions with minor modifications.
- Cells were washed twice with pre-cooled PBS, gently scraped, collected into nuclease-free tubes, and pelleted by centrifugation at 1,500 rpm, 4 °C for 5 min. The cell pellets were resuspended in an equal volume of complete RIP lysis buffer (Merck, CS203176) and incubated on ice for 5 min to ensure complete lysis. Lysates were aliquoted (~200 µL per tube) and stored at −80 °C until use.
- Magnetic Beads Protein A/G (Merck, Cat. No. CS203178) were resuspended thoroughly, and 50 µL beads per reaction were washed twice with RIP Wash Buffer. Beads were then incubated with 5 µg of anti-PCBP1 antibody (MBL, RN024P) or Normal Rabbit IgG (Merck, PP64B) at room temperature for 40 min with gentle rotation to allow antibody conjugation. After washing to remove unbound antibodies, beads were resuspended in RIP Wash Buffer and kept on ice.
- For immunoprecipitation, cell lysates were thawed on ice and clarified by centrifugation at 14,000 rpm, 4 °C for 10 min. 100 µL of supernatant was added to 900 µL RIP Immunoprecipitation Buffer containing the antibody–bead complexes (final volume 1 mL). An additional 10 µL of lysate was reserved as the Input control and stored at −80 °C. Samples were incubated at 4 °C for 3 h to overnight with rotation.
- Following incubation, beads were washed five times with pre-cooled RIP Wash Buffer to remove non-specifically bound material. Prior to the final wash, 50 µL of bead suspension was collected for Western blot analysis to confirm immunoprecipitation efficiency.
- RNA was extracted from the immunoprecipitated complexes using TRIzol^™^ LS Reagent (Invitrogen, 10296010CN). Bead–RNA–protein complexes and Input samples were treated with Proteinase K buffer and incubated at 55 °C for 30 min with shaking to digest proteins. After adjustment to a final volume of 250 µL, 750 µL TRIzol^™^ LS was added, and RNA extraction was performed as described in Sect. 2.6. Purified RNA was subjected to RT-qPCR, and primer sequences are listed in Table 3.

### Subcellular fractionation

Subcellular fractionation was performed using human primary macrophages, THP-1-derived macrophages, and RAW264.7 cells, with 6 × 10⁶ cells per group, using the Nuclear and Cytoplasmic Protein Extraction Kit (Beyotime, P0027) according to the manufacturer’s protocol.

Cytoplasmic and nuclear extraction reagents were thawed, placed on ice, and supplemented with freshly prepared PMSF (1 mmol/L) prior to use. Cells were washed once with 1× PBS, scraped, collected by centrifugation at 800 rpm for 5 min, and the supernatant was removed completely.

Cell pellets were resuspended in Cytoplasmic Protein Extraction Reagent A containing PMSF (200 µL per 20 µL pellet), vortexed vigorously for 5 s, and incubated on ice for 12 min. Cytoplasmic Protein Extraction Reagent B (10 µL) was then added, followed by vortexing and incubation on ice for 1 min. Samples were centrifuged at 16,000 × g, 4 °C for 5 min, and the supernatants were collected as the cytoplasmic fraction.

For nuclear protein extraction, residual supernatant was removed, and the pellet was resuspended in Nuclear Protein Extraction Reagent containing PMSF. Samples were vortexed vigorously every 1–2 min during a 30 min incubation on ice. After centrifugation at 16,000 × g, 4 °C for 10 min, the supernatant was collected as the nuclear fraction.

Equal volumes of 5× loading buffer were added to cytoplasmic and nuclear extracts, boiled for 5 min, and used for subsequent Western blot analysis.

### Ferroptosis and functional assays

#### GPX4 activity quantification

The GPX4 enzyme activity was indirectly detected using Beyotime Biotechnology’s Cellular Glutathione Peroxidase Assay Kit (Beyotime, S0056). The principle is that glutathione peroxidase (GPx) catalyzes the generation of GSSG from GSH, and glutathione reductase uses NADPH to reduce GSSG to GSH. The decrease in NADPH at A340 is measured to calculate GPx activity, which shows a linear relationship. The cell lysis buffer used was RIPA Lysis Buffer (Western and IP Cell Lysis Buffer, Beyotime, Cat# P0013); the specific composition is proprietary and not disclosed by the supplier. To inhibit proteolysis and dephosphorylation, PMSF (Protease Inhibitor) and Phosphatase Inhibitor Cocktail (specific catalog numbers provided previously) were added to the lysis buffer immediately before use. After lysis, protein concentration was determined using the BCA Protein Assay Kit (Solarbio, catalog: PC0020). Based on the measured concentration, samples were diluted as necessary with lysis buffer to ensure equal protein concentration across all samples for downstream analysis. The peroxide reagent used was tert-Butyl hydroperoxide (t-Bu-OOH). In this study, the control group (blank) was used as a reference. The absorbance at 340 nm (A340) for each group at different time points was plotted against time. The slope of this curve represents the rate of NADPH consumption, which is used to indirectly determine GPX4 enzyme activity.

#### Iron and redox homeostasis

Fe²⁺: Using Dojindo’s FerroOrange reagent to detect Intracellular Fe²⁺. FerroOrange is a novel fluorescent probe for Fe²⁺ imaging in live cells. It specifically detects unstable Fe²⁺, localizes in the endoplasmic reticulum, and generates an irreversible fluorescent product (Ex=543 nm, Em=580 nm) upon reaction with Fe²⁺. Neither Fe³⁺ nor other divalent metal ions enhance its fluorescence. THP-1 cells were used at 6 × 10^5^ cells/well and RAW264.7 cells at 3 × 10^5^ cells/well. PMA was added (final concentration of 50 ng/ml) to the cell culture medium to induce differentiation of THP-1 cells into macrophages over a period of 24 h. During this induction, the cells gradually adhered to the surface and changed in morphology from suspended monocytes to adherent macrophages. After induction, the cells were washed twice with RPMI 1640 medium and then add complete medium without PMA. Prior to staining, the cells were washed 2–3 times with pre-warmed serum-free medium. Serum-free medium containing 1 µmol/L FerroOrange was added and incubated at 37 °C with 5% CO₂ for 30 min. No washing is required after incubation (as changing the medium could lead to leakage of FerroOrange out of the cells). Finally, an appropriate amount of pre-warmed serum-free medium was supplemented, and the cells were immediately observed under a fluorescence microscope or flow cytometer.

ROS: DCFH-DA is a cell-permeable fluorescent dye that, upon entering the cell, is hydrolyzed by intracellular esterases to DCFH, which cannot pass through the cell membrane and thus accumulates inside the cell. Under the action of ROS, DCFH is oxidized to the fluorescent product DCF, whose fluorescence intensity can be detected by a fluorescence microscope or flow cytometer (Ex = 488 nm, Em = 525 nm). Procedure: Wash the cells 2–3 times with 1×PBS, add 2.5µmol/L DCFH-DA prepared in serum-free medium, and incubate for 30 min at 37 °C in a 5% CO₂ incubator while avoiding light. Then, wash 2–3 times with 1×PBS to remove excess probe, add fresh 1×PBS, and observe under a fluorescence microscope (green fluorescence at 488 nm) or collect cells via trypsin digestion for analysis using a flow cytometer.

Lipid Peroxidation: Lipid peroxidation in live cells can be dynamically monitored using two distinct fluorescent probes: Liperfluo (a water-soluble perylene derivative modified with a tetraethylene glycol group) and C11-Bodipy (BODIPY^™^ 581/591 C11). Liperfluo specifically reacts with lipid peroxides in hydrophobic environments, exhibiting enhanced fluorescence at excitation/emission wavelengths of 524/535 nm upon oxidation, with minimal aqueous background interference. In contrast, C11-Bodipy undergoes a spectral shift from red (591 nm) to green (581 nm) emission upon interaction with lipid radicals, enabling real-time tracking of peroxidation progression. Liperfluo is supplied by Dojindo (catalog: L-248) with a final concentration of 0.83 µg/mL. C11-Bodipy is also supplied by Dojindo (catalog: L-267), prepared according to the reagent instructions by diluting 1000-fold for use. For both assays, cells are washed twice with pre-warmed serum-free medium or 1×HBSS, followed by probe loading: Liperfluo (10 µM in serum-free medium, 37 °C, 30 min) or C11-Bodipy (manufacturer-recommended concentration in RPMI 1640, 37 °C, 30 min). Post-incubation, cells are washed twice, resuspended in fresh buffer, and analyzed via fluorescence microscopy or flow cytometry.

The flow cytometers used werfe BD FACSCelesta (BD Biosciences) and NovoCyte Opteon (Agilent). The gates were first set based on forward scatter (FSC) and side scatter (SSC) signals to exclude debris and doublets, selecting single-cell populations. For fluorescence channels, Fe²⁺ detection used Ex = 543 nm, Em = 580 nm channel; ROS and Liperfluo detection used Ex = 488 nm, Em = 525 nm channel; C11-Bodipy detection (green fluorescence: FITC, red fluorescence: Cy3). Thresholds for positive cells were established using unstained controls and fluorescence compensation. When calculating mean fluorescence intensity (MFI), FlowJo software was used to statistically analyze the target cell population; when calculating the percentage of cells, they were divided into positive and negative groups based on established thresholds, with the software automatically tallying the percentage of each group relative to the total number of cells.

#### Cell viability and death

THP-1 cells were seeded at 1 × 10⁴ cells/well (96-well plate); RAW264.7 cells were seeded at 5 × 10³ cells/well (96-well plate), using 100 µl of culture medium. The CCK-8 kit was purchased from Dojindo (catalog: CK04). First, cells were harvested and counted, adjusting the cell concentration to 1 × 10⁶ cells/ml. Take 100 µl of the cell suspension and add it to flow cytometry tubes, add 0.1 µl of Ghost Dye^™^ Red 780 stain (Cytek Biosciences, 13–0865), mix gently, and incubate in the dark at 4℃ for 30 min. After incubation, wash cells twice with 1× PBS for 5 min each time, centrifuging at 1500 rpm. Then, resuspend cells using 1×PBS and analyze by flow cytometry (excitation = 488 nm, emission = 525 nm channel, i.e., APC-Cy7 channel). For analysis, gating was first performed based on forward scatter (FSC) and side scatter (SSC) signals to exclude debris and doublets and select the single cell population. The threshold for positive cells was determined using unstained controls. When calculating the percentage of cell death, cells were divided into positive and negative groups based on the set threshold, and the software automatically calculated the percentage of cells in each group relative to the total cell count.

### Intracellular protein flow cytometry detection

Human primary macrophages, THP-1-derived macrophages, and RAW264.7 cells were used. The number of cells per experimental group was 1 × 10⁶. The cell processing conditions before flow cytometry analysis were as follows: After cells underwent different experimental treatments (e.g., infection, drug treatment, etc.), they were washed twice with ice-cold 1× PBS and resuspended in an appropriate volume of 1× PBS at a density of 1 × 10⁶ cells/ml, ready for intracellular protein flow cytometry analysis. The permeabilization reagent used was BD Cytofix/Cytoperm^™^ Fixation and Permeabilization Solution (BD, catalog: 554722), prepared and used according to the manufacturer’s instructions. The permeabilization washing buffer was BD Perm/Wash^™^ Perm/Wash Buffer (BD, catalog: 554723). The primary antibody information was Anti-PCBP1 antibody (Boster, catalog: A02636-1, dilution 1:100, unconjugated) and anti-GPX4 antibody (Abcam, catalog: ab125066, dilution 1:400, unconjugated). The secondary antibody used was Goat anti-rabbit IgG H&L (Alexa Fluor^®^ 488) (abcam, ab150077), dilution 1:3000. For flow cytometry analysis, the fluorescent label was Alexa Fluor 488, corresponding to the FITC channel (Ex = 495 nm, Em = 519 nm). The cell pellet was resuspended in 200 µL of 4% paraformaldehyde (Beyotime, catalog: P0099) and fixed at room temperature for 15 min. After fixation, cells were immediately analyzed by flow cytometry or stored at 4℃ for later use. The flow cytometer model was BD FACSCelesta (BD Biosciences). For analysis, gating was first performed based on forward scatter (FSC) and side scatter (SSC) signals to exclude debris and doublets and select the single cell population. The threshold for positive cells was determined using unstained controls. Statistical analysis of the target cell population, including the calculation of the Mean Fluorescence Intensity (MFI) for protein expression, was performed using FlowJo software

### Intracellular Mycobacterial Survival Assay

This assay assessed intracellular bacterial survival by lysing cells and plating diluted lysates. THP-1 differentiated macrophages and RAW264.7 macrophages were infected with Mtb H37Ra (ATCC 25617) at an MOI of 10. Cells were collected at various time points post-infection (e.g., 6, 12, 24, and 48 h), followed by bactericidal assays. After collecting extracellular medium into an EP tube and washing cells three times with 1×PBS, cells are lysed with sterile 0.01% SDS solution at 37 °C in a 5% CO₂ incubator for 15 min. The lysate is then transferred to a 15 mL centrifuge tube, diluted with sterile PBS to appropriate concentrations, and plated onto 7H11 agar plates. After incubation at 37 ℃ for 2–4 weeks, colony-forming units (CFU) are counted to determine bacterial concentration.

### LNP synthesis and characterization

#### LNP preparation and saRNA encapsulation

Lipid nanoparticles (LNPs) were formulated through optimization of lipid composition to achieve tissue- and cell-specific delivery. The ionizable lipid DLin-MC3-DMA (hereafter referred to as MC3), DOPC, cholesterol (Chol), DMG-PEG or mannose-DMG-PEG, and DOTAP were mixed at a molar ratio of 50:10:38.5:1.5:100, corresponding to a final 50% molar fraction of DOTAP within the total lipid composition [43, 49]. The 50% DOTAP molar ratio was selected based on the SORT (selective organ targeting) strategy [49], which maximizes pulmonary accumulation while reducing hepatic uptake. Mannose modification was introduced to target mannose receptors (CD206) on alveolar macrophages, as previously validated [42].

For preparation of empty LNPs or mannose-modified LNPs (MLNPs), 5 mg total lipids were dissolved in absolute ethanol and dried by rotary evaporation at 52 °C to form a uniform lipid film. The film was hydrated with 500 µL nuclease-free water at the same temperature, followed by sonication at 100 W (5 s on/5 s off) for 10 min. The resulting liposomes were sequentially extruded through 200 nm and 100 nm polycarbonate membranes (10 passes each) at 37 °C to ensure homogeneous particle size. Prepared LNPs/MLNPs were stored at 4 °C prior to use.

Encapsulation of saRNA was performed using a post-insertion incubation method under acidic conditions. LNPs were mixed with saRNA in 0.1 M trisodium citrate buffer (pH 4.5) (Acmec, Cat. No. 68-04-2) at a volume ratio of 1:3 (LNPs: saRNA) and a mass ratio of 20:1 (total lipid mass: saRNA mass). The total lipid concentration was 10 mg/mL, and the initial saRNA concentration was 0.264 mg/mL. The mixture was incubated at room temperature or 37 °C for 15 min to facilitate encapsulation. Subsequently, LNP-saRNA complexes were pelleted by centrifugation at 12,000 rpm for 10 min, washed with nuclease-free water to remove unencapsulated saRNA, and resuspended for downstream applications.

The saPCBP1 formulation used in functional and in vivo experiments consisted of a mixture of four saRNAs (mmu-PCBP1-Sa1, -Sa2, -Sa3, and -Sa4), with sequences listed in Table 5. For in vitro experiments, LNP-saRNA complexes were freshly prepared at required concentrations. For in vivo studies, the final injection volume was 100 µL per mouse, and formulations were used immediately after preparation without storage.

**Table 5:** saRNA sequence information

Name	Sequence (5’→ 3’)
NC-sa	sense: UUCUCCGAACGUGUCACGUTT antisense: ACGUGACACGUUCGGAGAATT
mmu-PCBP1-Sa1	sense: UUUGUUAACCCUAGCGGCUTT antisense: AGCCGCUAGGGUUAACAAATT
mmu-PCBP1-Sa2	sense: UUCGAUGGCGGAGCGAUACTT antisense: GUAUCGCUCCGCCAUCGAATT
mmu-PCBP1-Sa3	sense: UGAGAGGGAGCACGUGUUUTT antisense: AAACACGUGCUCCCUCUCATT
mmu-PCBP1-Sa4	sense: UCCGCCCAAGGAUUCGAUGTT antisense: CAUCGAAUCCUUGGGCGGATT

#### Physicochemical characterization

Particle size, polydispersity index (PDI), and zeta potential were measured using a Brookhaven NanoBrook 90Plus analyzer. LNPs were diluted to 0.1–1 mg/mL in RNase-free deionized water and briefly sonicated if necessary. Each sample was measured at least three times, and PDI values < 0.3 were considered indicative of narrow size distribution.

Morphology was examined by transmission electron microscopy (TEM). Diluted nanoparticle suspensions were applied (10–15 µL) onto carbon-coated copper grids and adsorbed for 1 min, followed by negative staining with 2% phosphotungstic acid (YEASEN, Cat. No. 60414ES60). Excess stain was removed, and grids were air-dried prior to imaging.

#### Encapsulation efficiency and in vitro release

Encapsulation efficiency (EE) of saRNA was quantified using the Quant-iT^™^ RiboGreen^®^ RNA assay (Thermo Fisher Scientific). Free saRNA was measured in the supernatant following nanoparticle separation, while total saRNA was determined after nanoparticle lysis with 1% Triton X-100. EE was calculated as: *EE (%) = (Total saRNA − Free saRNA)/Total saRNA ×100%.* In vitro release kinetics were assessed using a dialysis method (MWCO 50 kDa). Nanoparticles were dialyzed at 37 °C against PBS (pH 7.4) supplemented with 50% heat-inactivated FBS, 200 U/mL murine RNase inhibitor, and 5 mM EDTA. After 8 h, dialysis bags were transferred to acetate buffer (pH 5.5) containing identical supplements to mimic endosomal conditions. Samples were collected at predefined time points (0–72 h), and released saRNA was quantified by RiboGreen assay. Cumulative release was calculated relative to total saRNA determined from lysed 0-h controls.

#### In Vivo delivery and bioluminescence imaging

Luciferase mRNA (Luc-mRNA; GenScript) was encapsulated into LNPs/MLNPs using the same post-insertion acidic incubation method described above. The lipid: Luc-mRNA mass ratio was 20:1, with final lipid and mRNA concentrations of 10 mg/mL and 1 mg/mL, respectively.

For in vivo studies, LNP-saRNA formulations were administered via tail vein injection at a dose of 0.3 mg/kg (saRNA). Bioluminescence imaging was performed using a Caliper Life Sciences IVIS system, with parameters set as follows: camera temperature −90 °C, stage temperature 37 °C, exposure time 60 s, medium binning, and f/stop = 1. Images were analyzed using Living Image 4.3.1, and average radiance within regions of interest (ROI) was quantified.

### Histology and imaging

#### TEM and immunofluorescence

For TEM analysis, mice were euthanized by cervical dislocation, and lung and spleen tissues were excised and fixed in 2.5% glutaraldehyde for 2 h, followed by 1% osmium tetroxide post-fixation. Samples were dehydrated through graded ethanol, embedded in resin, polymerized, sectioned, and imaged by TEM following standard protocols. For immunofluorescence, cells were seeded on coverslips (~5 × 10⁴ cells/well), fixed with 4% paraformaldehyde, permeabilized with −20 °C methanol, blocked with 5% BSA, and incubated overnight at 4 °C with primary antibodies. Alexa Fluor-conjugated secondary antibodies were applied for 1 h, nuclei were counterstained with DAPI, and images were acquired using a Nikon Ti2-U microscope.

The primary antibody information were anti-PCBP1 antibody (Boster, catalog: A02636-1, dilution 1:200, unconjugated), anti-PCBP1 antibody (Santa Cruz Biotechnology, catalog: sc-137249, dilution 1:200, unconjugated), anti-GPX4 antibody (abcam, catalog: ab125066, dilution 1:200, unconjugated), anti-Trim21 antibody (BosterBio, catalog: A02079-2, dilution 1:200, unconjugated), and anti-TFR-1 antibody (BosterBio, catalog: PB9233, dilution 1:100, unconjugated). The secondary antibodies were Alexa Fluor 488-labeled Goat anti-rabbit IgG (abcam, catalog: ab50077, dilution 1:500), Alexa Fluor 555-labeled Goat anti-rabbit IgG (abcam, catalog: ab150078, dilution 1:500), Alexa Fluor 488-labeled Goat anti-mouse IgG (abcam, catalog: ab150113, dilution 1:500), and Alexa Fluor 555-labeled Goat anti-mouse IgG (abcam, catalog: ab150118, dilution 1:500).

#### Histopathology

Freshly harvested mouse lung, liver, kidney, and spleen tissues were immediately immersed in 4% paraformaldehyde (Sigma-Aldrich, Cat. No. P6148) and fixed for 24–48 h at room temperature. The volume of fixative was maintained at 10–20 times the tissue volume to ensure adequate fixation. After fixation, tissues were thoroughly rinsed under running tap water for 15–30 min to remove residual fixative.

The fixed tissues were subsequently dehydrated through a graded ethanol series consisting of 70%, 85%, 95%, and 100% ethanol, with each dehydration step lasting 30 min, and the 100% ethanol step performed twice to ensure complete dehydration. Dehydrated tissues were then cleared in xylene, with the xylene solution replaced every 10 min for a total of three changes, followed by paraffin infiltration and embedding using a tissue processing system with the paraffin temperature maintained at approximately 60 °C. During embedding, tissues were placed into paraffin molds, filled with molten paraffin, and allowed to solidify, followed by further hardening at 37 °C and subsequently at 4 °C for 1–2 h to ensure complete paraffin solidification.

Paraffin-embedded tissue blocks were sectioned at a thickness of 3.5 μm using a Leica RM2255 microtome, and the sections were mounted onto glass slides for subsequent histological and immunohistochemical analyses.

For histological evaluation, hematoxylin and eosin (H&E) staining was performed. Harris Hematoxylin (Sigma-Aldrich, Cat. No. MHS16) and 0.5% acidic Eosin Y (Sigma-Aldrich, Cat. No. E4382) were prepared and applied according to the manufacturers’ instructions. For immunohistochemical staining, a commercial DAB chromogenic kit (Boster, Cat. No. 34572) was used following the manufacturer’s protocol, and hematoxylin was applied for nuclear counterstaining, consistent with that used in H&E staining. For immunohistochemical staining, paraffin sections were subjected to antigen retrieval, followed by incubation with primary antibodies against GPX4 and 4-hydroxynonenal (4-HNE). The primary antibodies used were anti-GPX4 (abcam, Cat. No. ab125066, 1:100 dilution, unconjugated) and anti-4HNE (Proteintech, Cat. No. 27308-1-AP, 1:200 dilution, unconjugated). After incubation with primary antibodies, sections were incubated with HRP-conjugated secondary antibodies, including goat anti-rabbit IgG (Thermo Fisher Scientific, Cat. No. 31460, 1:500 dilution) or goat anti-mouse IgG (Thermo Fisher Scientific, Cat. No. 31460, 1:500 dilution), as appropriate. Signal development was performed using a commercial DAB chromogenic kit, followed by hematoxylin counterstaining.

Histopathological evaluation of lung tissues was performed on H&E-stained sections using a blinded semi-quantitative scoring system to assess pulmonary injury. Three pathological parameters, including inflammatory cell infiltration, alveolar structural damage, and granuloma formation, were evaluated. Each parameter was scored on a scale from 0 (none/normal) to 3 (severe) according to predefined criteria (Supplementary Table 1). For each animal, five randomly selected fields per lung section were examined at 200× magnification by two independent investigators in a blinded manner. The scores obtained from all fields were averaged to generate a mean score for each parameter per mouse, and a total pathology score (range 0–9) was calculated and used for subsequent inter-group statistical comparisons.

### Detection of pro-inflammatory cytokines in organ homogenates

To assess whether nanoparticles elicited a local immune response in major off-target organs, levels of key pro-inflammatory cytokines were measured in tissue homogenates using enzyme-linked immunosorbent assay (ELISA).

Following euthanasia, lung, liver, spleen, and kidney tissues were rapidly harvested, weighed, and homogenized in ice-cold PBS containing protease inhibitors. The homogenates were centrifuged at 12,000 × g for 15 min at 4℃. The supernatants were collected and stored at −80℃ for subsequent analysis.

ELISA was performed strictly according to the manufacturer’s instructions of a commercial ELISA kit (Mouse TNF-α/IL-6/IL-1β ELISA Kit, purchased from Servicebio Technology Co., Ltd.). Briefly, standards and samples were added to antibody-precoated microwells. After incubation and plate washing, biotinylated detection antibodies were added, followed by another incubation and wash step. Streptavidin-horseradish peroxidase (HRP) conjugate was then added, followed by tetramethylbenzidine (TMB) substrate for color development. The reaction was terminated with stop solution. Absorbance was measured immediately at 450 nm with a reference wavelength of 630 nm using a microplate reader. Cytokine concentrations were determined by interpolation from a standard curve generated from known concentrations of standards. Final results are expressed as picograms of cytokine per milligram of protein (pg/mg protein) or as relative values for intergroup comparison.

### Hematological analysis in mice

This procedure outlines the process of conducting a complete blood count (CBC) in mice to assess their health, diagnose diseases, and monitor experimental interventions by measuring key blood parameters. The process involves anesthetizing the mouse with isoflurane, performing a retrobulbar bleeding procedure by disinfecting the eye area, and then using forceps and scissors to expose and remove the eyeball to collect blood into an EDTA-K_2_-coated tube. The blood sample is then analyzed using a blood analyzer to obtain parameters such as white blood cell count, red blood cell count, hemoglobin concentration, platelet count, hematocrit, mean corpuscular volume, and mean corpuscular hemoglobin content. The supplier and model of the 5-part hematology analyzer used for animals was Dymind, DF55 Vet. The blood routine parameters analyzed in this study included white blood cell count (WBC), neutrophil count, lymphocyte count, monocyte count, neutrophil percentage (%), lymphocyte percentage (%), and monocyte percentage (%).

### Preparation of lung tissue single-cell suspension

The groups were C57BL/6J mice infected with Mtb H37Ra: Control group (mice that received tail vein injections of enzyme-free sterile water), non-specific activation group 1 (mice injected with saNC@LNP via tail vein), non-specific activation group 2 (mice injected with saNC@MLNP via tail vein), specific activation group 1 (mice treated with saPCBP1@LNPs through tail vein injection), and specific activation group 2 (mice treated with saPCBP1@MLNPs after infection with H37Ra). The enzyme digestion solution contained collagenase type I (biosharp, catalog: BS163), at a concentration of 1 mg/mL, deoxyribonuclease I (DNase I, biosharp, catalog: BS137), at a concentration of 0.2 mg/mL, and hyaluronidase (biosharp, catalog: BS171), at a concentration of 0.2 mg/mL. Digestion was performed at 37 °C and 150 rpm for 1 to 1.5 h. The lung tissue was cut into pieces of 1–2 mm, which were placed in the enzyme digestion solution and incubated on a thermostatic shaker. After incubation, they were gently pipetted up and down using a 1 mL pipette at 4 °C until the tissue was completely dispersed. The cells were counted using a hemocytometer (counting chamber volume of 0.1 mm³) under an optical microscope. Cell viability was tested using the Trypan blue exclusion assay. The cell suspension was mixed equally with 0.4% trypan blue solution (Solarbio, catalog: C0040), incubated for 3–5 min, and observe under a microscope to count the stained (dead cells) and unstained (live cells). Cell viability was calculated (cell viability = number of live cells/total number of cells ×100%). The red blood cell lysis buffer was a commercial buffer (Thermo Fisher, catalog: 00–4333-53). The final cell suspension was resuspended in flow cytometry detection buffer (1×PBS containing 2% FBS), adjusted to 1 × 10^6^−5 × 10^6^ cells/ml. The total volume was determined based on experimental needs, approximately 100–500 µL. The prepared single-cell suspension was used for flow cytometric analysis to detect intracellular PCBP1 expression levels and evaluate ferroptosis-related indicators.

### Transcriptomics and bioinformatics

- Transcriptomic analysis was performed using THP-1-derived macrophages under different experimental conditions. The experimental groups included uninfected control cells, H37Rv-infected cells, PCBP1 knockdown cells (KO^−/+^), PCBP1 overexpression cells (OE), and their corresponding infection and vector control groups, including WT-Rv, KO^−/+^-Rv, Vec, Vec-Rv, and OE-Rv. Each group consisted of three independent biological replicates. Total RNA was extracted from cell samples using TRIzol reagent according to the manufacturer’s instructions.
- RNA integrity and purity were evaluated using an Agilent 2100 Bioanalyzer (Agilent Technologies). Only RNA samples with an RNA Integrity Number (RIN) ≥ 7.0, an A260/280 ratio between 1.8 and 2.2, and an A260/230 ratio between 2.0 and 2.5 were used for library construction. Libraries were prepared using the Illumina TruSeq Stranded mRNA Library Prep Kit (Illumina, Cat. No. RS-122–2103) and sequenced on an Illumina HiSeq X Ten platform using paired-end sequencing (150 bp). Approximately 30 million reads per sample were generated to ensure sufficient sequencing depth.
- Raw sequencing reads were processed by removing adapter sequences using Cutadapt (v2.10). Read quality was assessed using FastQC (v0.11.9), and low-quality reads (reads with > 20% bases having a quality score < 20) as well as short reads (< 36 bp) were filtered out using Trimmomatic (v0.39). Clean reads were aligned to the human reference genome GRCh38 using HISAT2 (v2.2.1) with parameters set to *-k 2* and *--threads 4*. Gene expression levels were quantified using StringTie (v2.1.3) and expressed as FPKM (Fragments Per Kilobase of transcript per Million mapped reads). For analyses involving mouse-derived data, the GRCm38 reference genome was used.
- Gene Ontology (GO), Kyoto Encyclopedia of Genes and Genomes (KEGG) pathway enrichment, and Gene Set Enrichment Analysis (GSEA) were performed using an integrated bioinformatics analysis platform based on R language packages, including clusterProfiler. Enrichment significance was evaluated using a p-value threshold of 0.05, and false discovery rate (FDR) correction was applied to control for multiple testing, with an FDR < 0.2 considered statistically significant. GO enrichment analyses covered the three major ontology categories: Biological Process, Cellular Component, and Molecular Function.

### Statistical and bioinformatics data analysis

In this study, we performed bioinformatics and statistical analyses to ensure the reliability of our experimental results. For bulk RNA-seq and RIP-seq data analysis, we used R (version 4.1.2) with the DESeq2 package to standardize data and identify differentially expressed genes. Flow cytometry data from the BD FACSVerse instrument were analyzed using FlowJo software, with compensation adjustments and precise gating to quantify cell phenotype changes. WB results were analyzed using ImageJ for band quantification and normalization to loading controls. Statistical analysis and graphing were performed using GraphPad Prism 10.0, with unpaired t-tests for two-group comparisons and one-way ANOVA with multiple comparisons correction for multi-group analyses. The version of FlowJo was v10.8.1. The version of ImageJ was 1.53t (NIH, https://imagej.nih.gov/ij/). A threshold of *P* < 0.05 was used for statistical significance across all analyses.

## Results

### Mtb drives ferroptosis in macrophages across clinical and experimental models

**Fig. 1:**
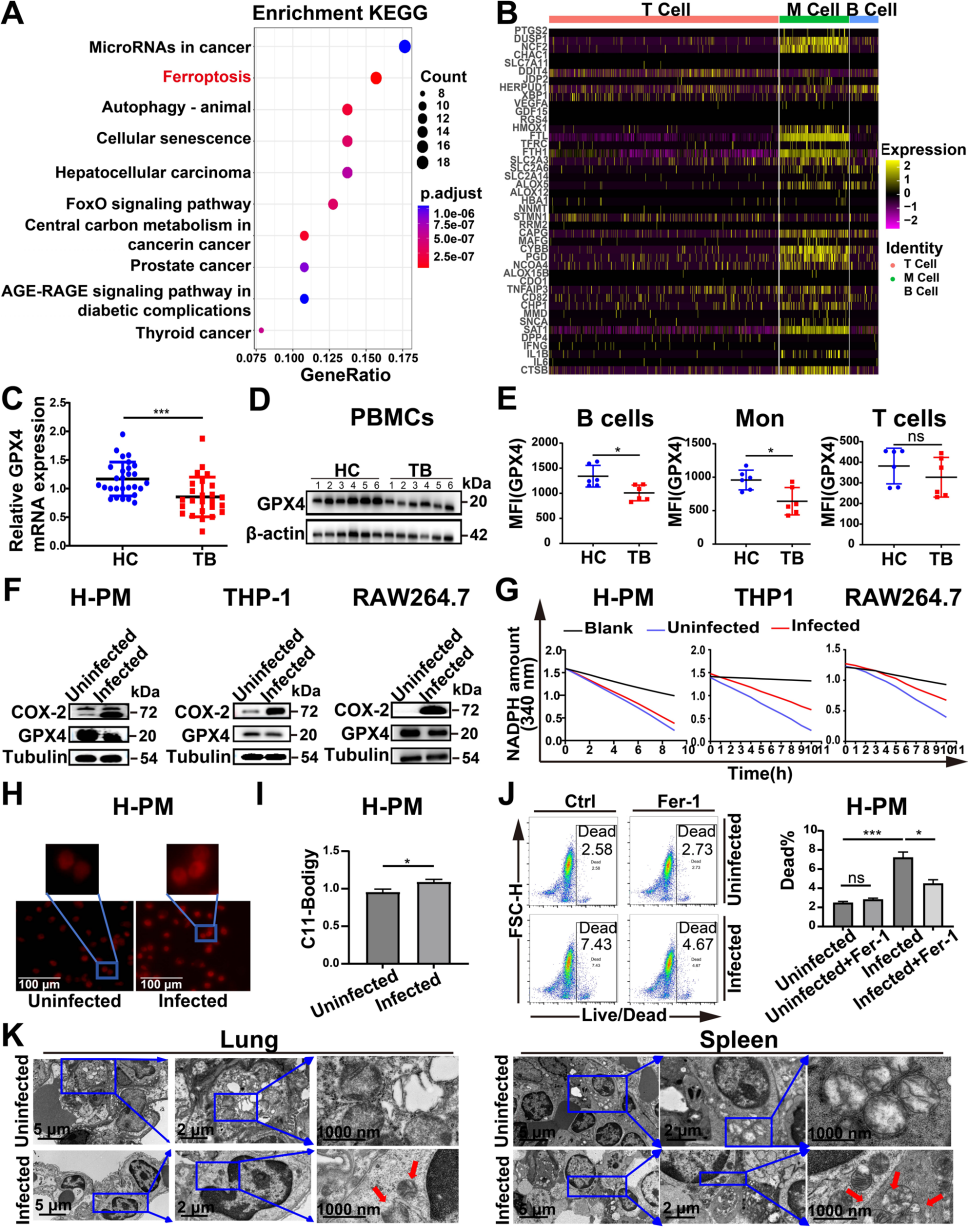
Ferroptosis is activated in myeloid cells during Mtb infection. (**A**) KEGG pathway analysis of DEGs showing significant enrichment of the ferroptosis pathway. Key nodes include HMOX1, SLC7A11, and lipid peroxidation-related genes. (**B**) Single-cell RNA-seq analysis of PBMCs from tuberculosis patients reveals myeloid-specific ferroptosis marker upregulation. (**C**) RT-qPCR analysis of GPX4 mRNA levels in PBMCs from active TB patients (*n* = 28) and healthy controls (*n* = 25). (**D**) WB quantification of GPX4 protein expression in PBMCs from TB patients (*n* = 6) and controls (*n* = 6). (**E**) Flow cytometry (FCM) analysis revealing lower GPX4 protein levels in B cells and monocytes from TB patients. (**F-G**) WB analysis of COX-2 and GPX4 protein expression (**F**) and measurement of GPX4 enzymatic activity (**G**) in Mtb-infected human primary macrophages, THP-1-derived macrophages, and RAW264.7 cells. (**H**) Fluorescence microscopy images displaying Fe²⁺ accumulation (red fluorescence) in Mtb-infected human primary macrophages. Scale bar, 100 μm. (**I**) FCM quantification of lipid peroxidation (BODIPY 581/591 C11 staining) in infected macrophages. (**J**) Cell death rates in Mtb-infected macrophages with or without Fer-1 treatment (10 µM). (**K**) TEM images of lung tissue from H37Rv-infected mice, highlighting mitochondrial shrinkage and cristae loss (indicated by red arrows). Scale bar, 5 μm (left); 2 μm (middle); 1000 nm (right). Data are presented as mean ± SD. Statistical significance was determined by Student’s t-test or ANOVA (*p < 0.05, *** *p* < 0.001)

This study used two Mtb strains: H37Ra (attenuated; suitable for in vitro mechanistic studies at high MOI, as well as for ethically compliant in vivo treatment models) and H37Rv (virulent; used to simulate the pathogenic mechanisms of clinical infection and for key phenotypic validation). The results obtained from the two Mtb strains complement each other, collectively confirming that PCBP1 is a core regulatory target in tuberculosis-induced ferroptosis. Considering that PCBP1 plays key roles in iron homeostasis and ferroptosis, PCBP1 emerged as a potential candidate as an orchestrator of Mtb immune escape for the subsequent experiments [18, 19]. In this study, we performed transcriptomic sequencing on THP-1-derived macrophages infected with the H37Rv strain. Gene Ontology (GO) enrichment analysis showed a significant association of differentially expressed genes (DEGs) with oxidative stress response and peroxisome function (Supplementary Fig. 1A). The Kyoto Encyclopedia of Genes and Genomes (KEGG) pathway analysis revealed pronounced enrichment in the ferroptosis pathway (Fig. 1A), corroborated by Gene Set Enrichment Analysis (GSEA) showing activated ferroptosis signaling (Supplementary Fig. 1B). Single-cell RNA sequencing of peripheral blood mononuclear cells (PBMCs) from TB patients identified four distinct populations: T cells (CD3+), B cells (CD79A+), myeloid cells (LYZ+), and an uncharacterized cluster. Cell-type-specific differential expression analysis demonstrated significant upregulation of core ferroptosis markers (PTGS2, HMOX1, SLC7A11, and ACSL4) specifically in the myeloid compartment compared to healthy controls (Fig. 1B). This myeloid-selective ferroptosis signature was maintained after rigorous batch effect correction and cellular composition adjustment using Harmony (v0.1.1). The upregulation of PTGS2, HMOX1, SLC7A11, and ACSL4 indicates the activation of the ferroptosis pathway.

RT-qPCR analysis of PBMCs from 28 active TB patients and 25 healthy controls revealed significantly lower mRNA levels of GPX4 in TB patients compared to controls (Fig. 1C), with concordant downregulation trends observed for CXCL2, FTH1, and JUN (Supplementary Figs. 1C-E). No significant differences were detected in SLC7A11, SLC3A2, VEGFA, DAZAP1, or SRC expression (Supplementary Figs. 1 F-J). Subsequent western blot (WB) and flow cytometry analyses of six paired clinical samples confirmed markedly reduced GPX4 protein levels in TB patient PBMCs (Fig. 1D, Supplementary Fig. 9A), with this downregulation predominantly localized to B-cell and monocyte subsets (Fig. 1E). GPX4 is a central suppressor of ferroptosis, and its downregulation represents another crucial manifestation of ferroptosis activation [50].

To investigate the direct link between Mtb infection and ferroptosis, we established infection models using human primary macrophages (H-PMs), THP-1-derived macrophages, and RAW264.7 macrophages. The MOI of 10 for the attenuated H37Ra strain was selected based on preliminary optimization experiments (Supplementary Fig. 2), which demonstrated that this dosage was required to ensure sufficient intracellular bacterial burden and induce ferroptosis phenotypes comparable to the virulent strain. WB demonstrated that Mtb infection consistently upregulated the ferroptosis marker COX-2 while downregulating GPX4 protein expression and enzymatic activity across all models (Figs. 1F-G, Supplementary Fig. 9B). WB analysis demonstrated consistent upregulation of COX-2 and downregulation of GPX4 protein expression across all macrophage models (Fig. 1F). Furthermore, GPX4 enzymatic activity was significantly reduced upon Mtb infection (Fig. 1G). Fluorescence microscopy and flow cytometry further revealed iron overload (Fig. 1H, Supplementary Figs. 1K-L) and elevated lipid peroxidation (Fig. 1I, Supplementary Figs. 1 M-N) in infected macrophages. These phenotypes were effectively reversed by the ferroptosis inhibitor Ferrostatin-1 (Fer-1) (Supplementary Figs. 1 M-N). Cell viability assays confirmed increased macrophage death post-infection (Fig. 1J, Supplementary Fig. 1O), which was attenuated by Fer-1 treatment. In H37Rv-infected mice, transmission electron microscopy of lung and spleen tissues exhibited hallmark ferroptotic morphological alterations, including mitochondrial shrinkage, membrane rupture, and cristae reduction (Fig. 1K).

Integrated analyses establish ferroptosis as a pathognomonic feature of Mtb-infected macrophages, driven by GPX4 axis suppression, iron-dependent lipid peroxidation, and mitochondrial cristae dissolution. This conserved mechanism, validated through in vitro (human/murine macrophages), in vivo (murine models), and clinical (PBMC scRNA-seq/RT-qPCR) tiers, reveals ferroptosis as a druggable vulnerability in TB pathogenesis.

### PCBP1 downregulation in mtb-infected macrophages and its association with ferroptosis

**Fig. 2:**
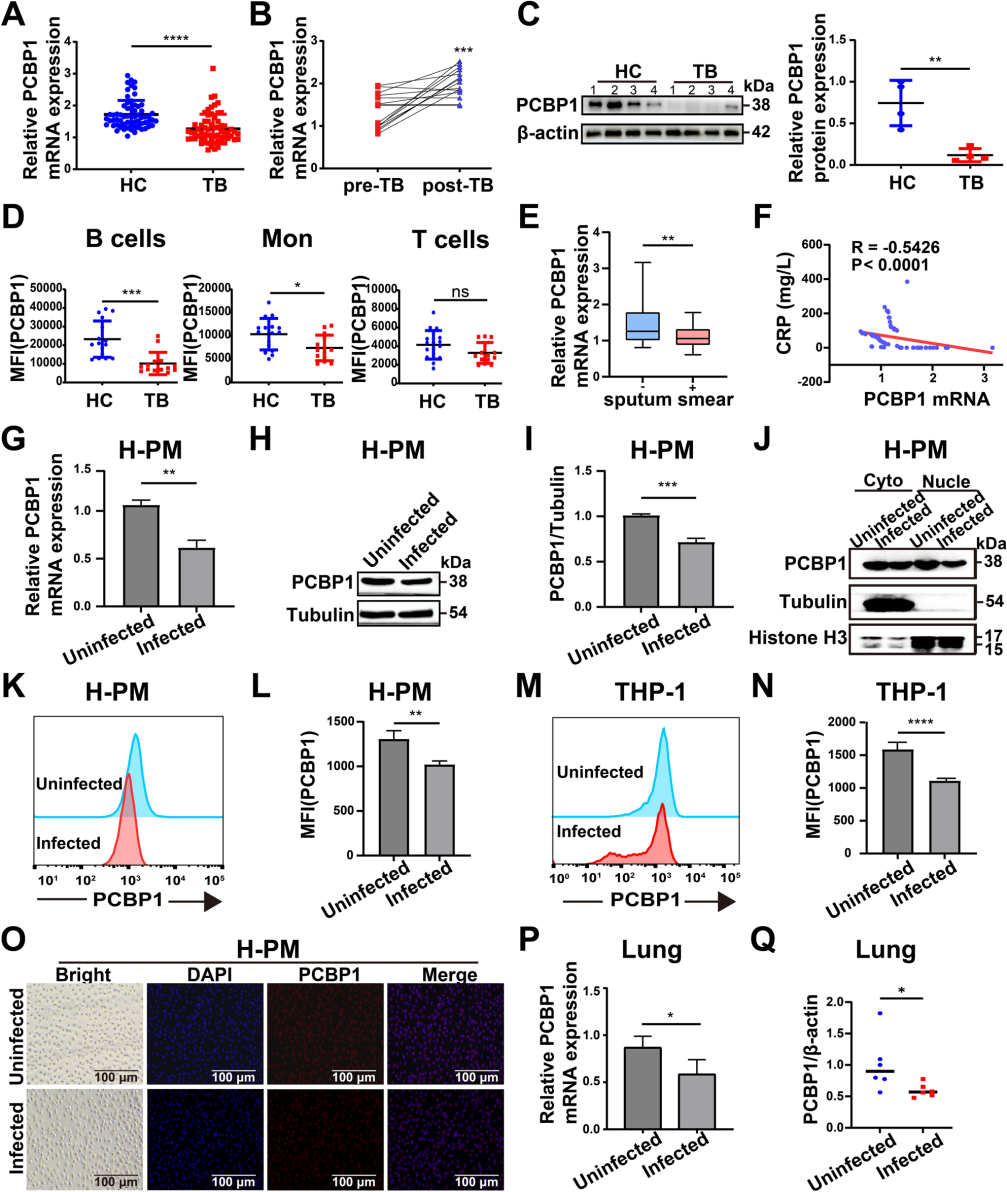
PCBP1 downregulation in Mtb infection: Evidence from clinical samples and experimental models. (**A**) RT-qPCR analysis showing significantly reduced PCBP1 mRNA levels in PBMCs from 64 active TB patients compared to 63 healthy controls. (**B**) PCBP1 mRNA expression in PBMCs from 15 TB patients exhibits significant upregulation after 1 month of standardized anti-TB therapy. (**C**) WB analysis revealing decreased PCBP1 protein expression in PBMCs from 4 active TB patients versus 4 healthy controls, with β-actin as a loading control. (**D**) Flow cytometry analysis demonstrating downregulated PCBP1 protein levels in B cells and monocytes from TB patients. (**E**) Comparison of PCBP1 mRNA levels in PBMCs from sputum smear-positive (*n* = 31) versus smear-negative (*n* = 33) pulmonary TB patients, as determined by RT-qPCR. Smear-positive status indicates higher mycobacterial burden. (**F**) Scatter plot showing a significant negative correlation between PCBP1 mRNA levels in PBMCs and serum CRP concentration in active TB patients (*n* = 64). CRP is a clinical marker of systemic inflammation and disease severity. The correlation coefficient (r) and P value are indicated (Pearson correlation). (**G**) RT-qPCR analysis confirming reduced PCBP1 mRNA expression in Mtb-infected macrophages. (**H-I**) WB analysis confirming reduced PCBP1 protein expression in Mtb-infected macrophages, with Tubulin as a loading control. (**J**) Nuclear-cytoplasmic fractionation assays showing decreased PCBP1 protein levels in both cytoplasmic and nuclear extracts of infected macrophages, using Histone H3 and Tubulin as markers. (**K-N**) Flow cytometry quantification of PCBP1 protein expression in Mtb-infected macrophages, presenting mean fluorescence intensity (MFI) values. (**O**) Fluorescence microscopy images depicting reduced PCBP1 protein (red) in Mtb-infected macrophages compared to uninfected cells, with nuclei stained by DAPI (blue). Scale bar, 100 μm. (P-Q) PCBP1 mRNA and protein expression analysis in lung tissues of Mtb-infected mice (*n* = 6) versus uninfected controls (*n* = 6), showing significant downregulation in infected mice. Data are presented as mean ± SD. Statistical significance was determined by Student’s t-test (*p < 0.05, **p < 0.01, ***p < 0.001, ****p < 0.0001)

A prospective cohort of 64 treatment-naive pulmonary TB patients and 63 age-/sex-matched healthy controls was enrolled (Table 1). The cohorts were comparable in age and sex distribution, while TB patients exhibited expected clinical markers of infection and inflammation, including elevated white cell counts, neutrophil percentages, and C-reactive protein (CRP) levels. After analyzing the GSE203261 dataset, we found significantly downregulated PCBP1 mRNA levels (Supplementary Fig. 3A). Though PCBP1’s role in ferroptosis is known, its involvement in Mtb infection remains unclear. Therefore, we detected PCBP1 mRNA expression in PBMCs from 64 active TB patients and 63 healthy individuals using RT-qPCR, finding lower expression in TB patients (Fig. 2A). Notably, in 15 TB patients after 1 month of standard treatment, PCBP1 mRNA levels in PBMCs significantly increased (Fig. 2B). WB also found decreased PCBP1 protein expression in PBMCs from four active TB patients compared with healthy individuals (Fig. 2C). Flow cytometry analysis of PBMCs from 16 active TB patients and 14 healthy individuals revealed PCBP1 down-regulation mainly in monocytes, but also in B cells (Fig. 2D). To further evaluate the clinical relevance of PCBP1 downregulation, we performed correlation analyses between PCBP1 mRNA levels in patient PBMCs and key clinical parameters. First, patients with sputum smear-positive status, indicative of a higher mycobacterial burden, exhibited significantly lower PCBP1 mRNA levels compared to smear-negative patients (Fig. 2E). Second, a significant negative correlation was observed between PCBP1 mRNA levels and serum CRP concentration, a well-established marker of systemic inflammation and disease severity (Fig. 2F). Given that sputum smear-positive status is a direct clinical indicator of higher mycobacterial burden, these findings suggest that reduced PCBP1 expression is not merely associated with TB infection but may also reflect the intensity of bacterial load and the ensuing inflammatory response.

To explore the impact of Mtb infection on PCBP1 expression, we established macrophage infection models using H-PMs, PMA-differentiated THP-1 macrophages, and RAW264.7 murine macrophages, infected with the attenuated Mtb H37Ra strain at an MOI of 10 for 24 h. RT-qPCR showed significant PCBP1 mRNA down-regulation in infected H-PMs, THP-1-derived macrophages, and RAW264.7 macrophages (Fig. 2G, Supplementary Fig. 3B). WB analysis also indicated decreased PCBP1 protein expression in the infected group (Fig. 2H, Supplementary Figs. 3 C & 9 C). Both cytoplasmic and nuclear PCBP1 protein expression levels were significantly down-regulated (Fig. 2I-J, Supplementary Fig. 3D-E). Flow cytometry and fluorescence microscopy results confirmed this, showing reduced PCBP1 protein expression and fluorescence in infected cells (Fig. 2K-O, Supplementary Fig. 3F-I).

In a murine TB model, Mtb-infected mice showed significant PCBP1 mRNA and protein downregulation in lung tissues (Fig. 2P-Q), while spleen PCBP1 expression remained unchanged (Supplementary Fig. 3J-K). Overall, these data demonstrate that Mtb infection suppresses PCBP1 expression in macrophages across clinical, cellular, and animal models, implicating PCBP1 as a potential TB pathogenesis regulator.

### PCBP1 enhances macrophage bactericidal activity against Mtb by inhibiting ferroptosis

Previous studies have identified PCBP1 as a negative regulator of ferroptosis in other disease models, but whether PCBP1 modulates Mtb-induced ferroptosis remains unknown. Given the significant downregulation of PCBP1 in Mtb-infected macrophages (Fig. 2), we hypothesized that PCBP1 deficiency exacerbates ferroptosis to impair host defense. To test this, we knocked down PCBP1 in THP-1-derived and RAW264.7 macrophages using siRNA, and overexpressed PCBP1 using adenoviral vectors. Successful knockdown and overexpression were confirmed by RT-qPCR and WB (Supplementary Fig. 4A-D).

**Fig. 3:**
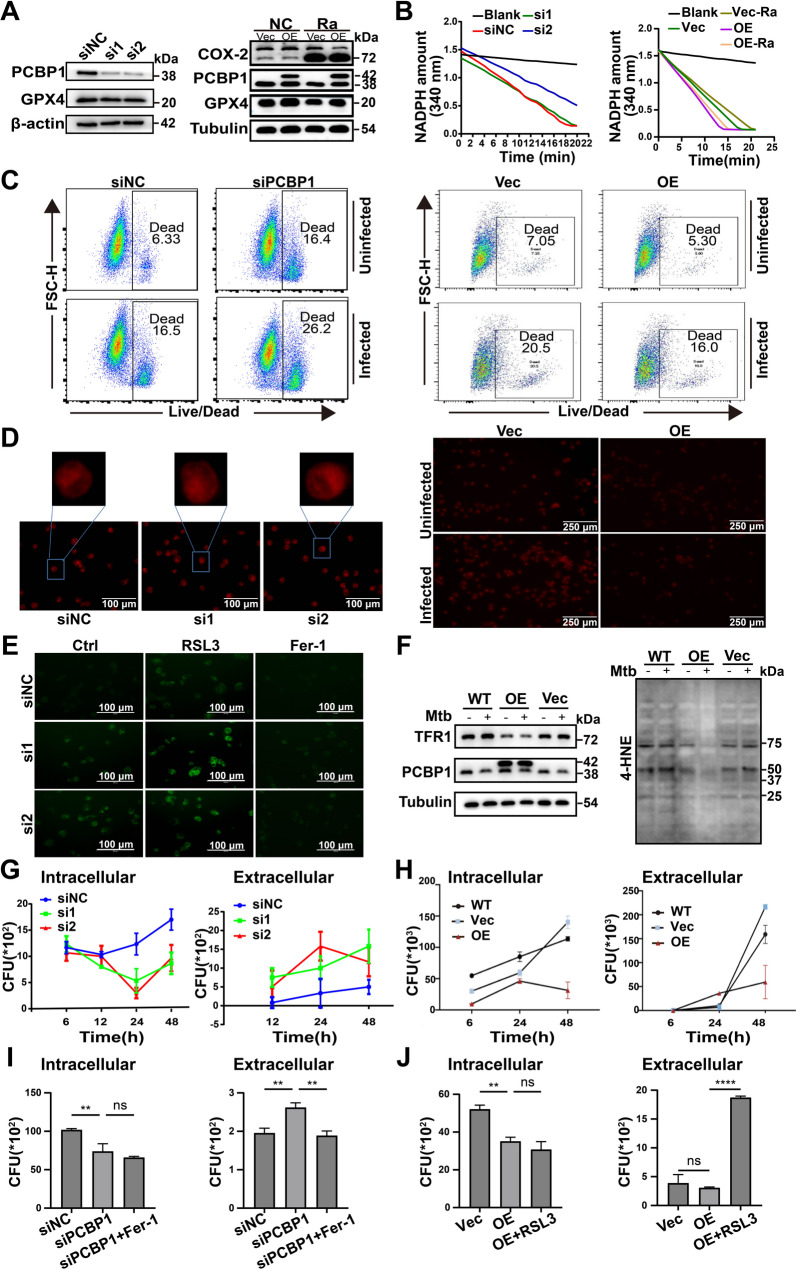
PCBP1 suppresses Mtb-induced ferroptosis to enhance macrophage antimycobacterial activity. (**A**) PCBP1 knockdown reduced GPX4 protein levels, while PCBP1 overexpression significantly upregulated GPX4 and reduced COX-2 protein levels. (B) PCBP1 knockdown significantly reduced GPX4 enzymatic activity, whereas PCBP1 overexpression enhanced GPX4 activity, indicating a positive regulatory role of PCBP1 on GPX4 function. (**C**) Flow cytometry analysis revealed increased cell death in Mtb-infected macrophages following PCBP1 knockdown, while PCBP1 overexpression significantly reduced cell death, suggesting a protective role of PCBP1 in maintaining macrophage viability. (**D**) PCBP1 knockdown significantly elevated intracellular Fe²⁺ levels in THP-1 macrophages, whereas PCBP1 overexpression suppressed Mtb-induced Fe²⁺ accumulation. Scale bar, 100 μm (left); 250 μm (right). (**E**) Lipid peroxidation (BODIPY 581/591 C11 assay). PCBP1 knockdown increased peroxidation, exacerbated by RSL3 (10 µM, 24 h) and rescued by Ferrostatin-1 (Fer-1, 10 µM, 24 h). Scale bar, 100 μm. (**F**) WB of transferrin receptor 1 (TFR1) and 4-HNE. PCBP1 overexpression downregulated both markers. (G-H) Intracellular and extracellular Mtb CFU in PCBP1-modulated macrophages (MOI = 10, 6, 12, 24, and 48 h). Knockdown reduced intracellular CFU but increased extracellular release (**G**), while overexpression suppressed both (**H**). (**I**) CFU assays showing intracellular and extracellular bacterial loads in control (siNC) or PCBP1-knockdown (siPCBP1) THP-1-derived macrophages infected with H37Ra (MOI = 10, 24 h), with or without co-treatment with Fer-1 (10 µM). (**J**) CFU assays in macrophages overexpressing empty vector (Vec) or PCBP1 (OE-PCBP1) and infected with H37Ra, with or without co-treatment with the ferroptosis inducer RSL3 (10 µM). Data are presented as mean ± SD. Statistical significance was determined by ANOVA (**p < 0.01, ****p < 0.0001

In PCBP1-knockdown THP-1 and RAW264.7 macrophages, GPX4 expression exhibited a downward trend (Fig. 3A, Supplementary Fig. 4E), accompanied by a significant reduction in its enzymatic activity (Fig. 3B, Supplementary Fig. 4F). In contrast, PCBP1 overexpression effectively attenuated Mtb-induced upregulation of COX-2 protein expression while enhancing GPX4 protein levels (Fig. 3A, Supplementary Fig. 9D) and enzymatic activity (Fig. 3B). Flow cytometry analysis revealed that PCBP1 knockdown increased Mtb-induced macrophage cell death, whereas PCBP1 overexpression reduced cell death rates (Fig. 3C).

Further analysis indicated that PCBP1 knockdown significantly elevated Fe²⁺ levels in THP-1 and RAW264.7 macrophages (Fig. 3D, Supplementary Fig. 4G), while PCBP1 overexpression suppressed Mtb-induced Fe²⁺ accumulation (Fig. 3D). In addition, PCBP1 knockdown led to a marked increase in intracellular ROS levels (Supplementary Figs. 4 H-I). PCBP1 knockdown significantly elevated lipid peroxidation levels in both cell lines, an effect that was further exacerbated by the ferroptosis inducer RSL3 (Fig. 3E, Supplementary Figs. 4 J). The RSL3 concentrations employed (10 µM for THP-1, 0.1 µM for RAW264.7) were optimized based on dose-response relationships established in preliminary experiments (Supplementary Figs. 4 K-L). However, treatment with the ferroptosis inhibitor Fer-1 effectively attenuated lipid peroxidation induced by PCBP1 knockdown. WB and immunofluorescence analyses further showed that PCBP1 overexpression significantly downregulated 4-HNE and TFR1 protein levels (Fig. 3F, Supplementary Figs. 4M & 9E).

Given the role of ferroptosis in Mtb immune evasion, we hypothesized that PCBP1 enhances macrophage bactericidal activity by suppressing ferroptosis. The results showed that PCBP1 knockdown, which promoted cell death (Fig. 3C), led to a decrease in intracellular Mtb colony-forming units (CFUs), likely due to cell lysis, accompanied by a significant increase in extracellular Mtb release (Fig. 3G, Supplementary Fig. 4N). PCBP1 overexpression suppressed cell death and significantly reduced both intracellular and extracellular Mtb CFUs, indicating enhanced host control over bacterial replication and survival (Fig. 3H). Sequencing analysis also revealed that differentially expressed genes were significantly enriched in the ferroptosis pathway (Supplementary Figs. 4O-P, Supplementary Table 2).

To establish the causal role of ferroptosis in PCBP1-mediated host defense, we conducted a series of rescue experiments integrating genetic manipulation and pharmacological modulation of the ferroptosis pathway.

It is important to note that intracellular CFU measures bacteria inside viable host cells, whereas extracellular CFU measures bacteria released from dead or ruptured cells, reflecting dissemination. A decrease in intracellular CFU with a concurrent increase in extracellular CFU (as seen in PCBP1 knockdown) indicates host cell lysis rather than enhanced killing.

First, CFU assay revealed that PCBP1 knockdown impaired the control of intracellular Mtb growth and increased extracellular bacterial release. Notably, concurrent treatment with the ferroptosis inhibitor Fer-1 completely rescued this phenotype, restoring both intracellular containment and limiting extracellular dissemination to control levels (Fig. 3I). Conversely, the enhanced bacterial clearance conferred by PCBP1 overexpression was significantly attenuated upon co-treatment with the ferroptosis inducer RSL3 (Fig. 3J).

Second, analysis of cell fate confirmed that the increased macrophage death induced by PCBP1 knockdown during Mtb infection was specifically abrogated by Fer-1 (Supplementary Fig. 4Q). Correspondingly, the protective effect of PCBP1 overexpression against infection-induced death was largely reversed by RSL3 (Supplementary Fig. 4R), directly linking the survival benefit to the suppression of ferroptosis.

Finally, examination of the core drivers of ferroptosis provided the mechanistic underpinning for these functional outcomes. WB analysis revealed that the elevated levels of 4-hydroxynonenal (4-HNE), a terminal metabolite of lipid peroxidation resulting from PCBP1 knockdown, were normalized to baseline by Fer-1 treatment (Supplementary Fig. 4S). Correspondingly, RSL3 treatment re-induced the increase of 4-HNE in PCBP1-overexpressing cells, effectively abolishing the antioxidant and iron-chelating benefits conferred by PCBP1 restoration (Supplementary Fig. 4T).

In summary, Mtb infection promotes ferroptosis in macrophages by downregulating PCBP1, thereby evading intracellular killing. Conversely, PCBP1 overexpression suppresses Mtb-induced ferroptosis and enhances macrophage bactericidal activity. Importantly, after PCBP1 knockdown, intracellular Mtb decreases while extracellular Mtb increases due to cell lysis, which illustrates the benefit of ferroptosis to Mtb: by killing the host cell, Mtb escapes from the cells and can disseminate to other cells, promoting infection. These findings highlight the critical role of PCBP1 in Mtb infection and identify it as a potential therapeutic target for tuberculosis.

### PCBP1 suppresses ferroptosis via its fe-binding domain

 Building on our findings that PCBP1 suppresses Mtb-induced ferroptosis to enhance macrophage bactericidal activity (Sect. 3.3), we next investigated the molecular basis of its iron chaperone function by targeting its Fe-binding domain. PCBP1, known to interact with ferritin and facilitate Fe²⁺ oxidation to Fe³⁺ for storage, is critical for iron homeostasis. To dissect the molecular basis of PCBP1’s iron chaperone function, we generated a PCBP1 mutant (D82A/E168A/E350A) predicted to disrupt Fe²⁺ coordination, according to a previous structural study [51]. This mutant was cloned into an adenoviral vector (HBAD-Adeasy-h-PCBP1ΔFe-3xflag-EGFP) for overexpression studies.

**Fig. 4:**
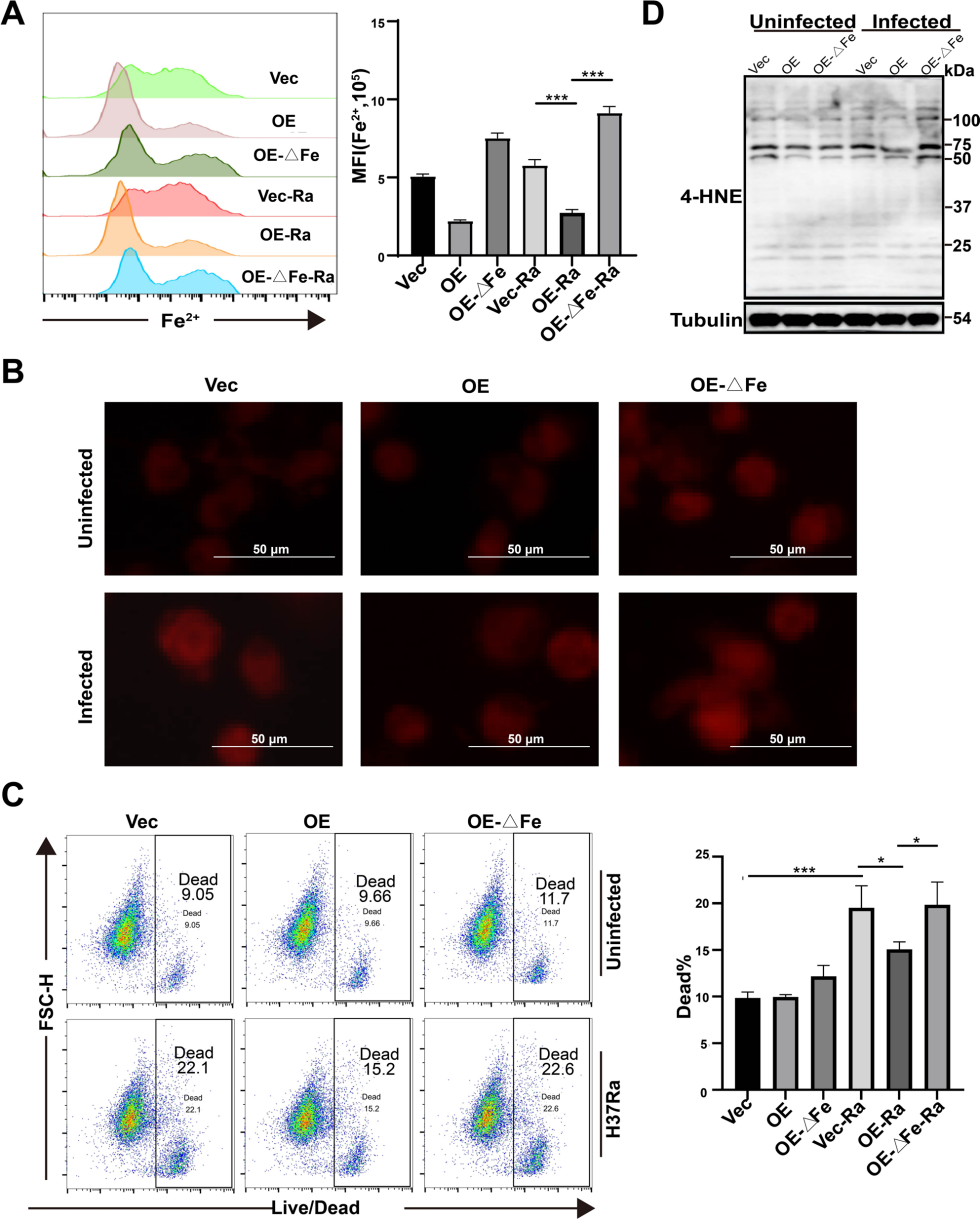
PCBP1 requires its Fe-binding domain to suppress ferroptosis in Mtb-infected macrophages. (**A**) FCM analysis of intracellular Fe²⁺ levels in THP-1-derived macrophages overexpressing wild-type or Fe-binding site-mutant PCBP1. Cells were infected with H37Ra (MOI = 10, 24 h). Wild-type PCBP1 overexpression significantly suppresses Mtb-induced Fe²⁺ elevation, whereas the Fe-binding site-mutant PCBP1 loses this inhibitory effect. (**B**) Fluorescence microscopy images of Fe²⁺ (red) in macrophages. Wild-type PCBP1 suppressed Mtb-induced Fe²⁺ elevation, while Fe-binding site-mutant PCBP1 failed to rescue this phenotype. Nuclei stained with DAPI (blue). Images representative of three independent experiments. Scale bar, 50 μm. (**C**) Cell death assays indicate that wild-type PCBP1 overexpression markedly decreases Mtb-induced macrophage death, an effect absent with the Fe-binding site-mutant PCBP1. (**D**) WB analysis reveals that wild-type PCBP1 overexpression significantly lowers the lipid peroxidation marker 4-HNE, an effect not observed with the Fe-binding site-mutant PCBP1. Data are presented as mean ± SD. Statistical significance was determined by ANOVA (*p < 0.05, ***p < 0.001, ****p < 0.0001)

Flow cytometry (Fig. 4A) and fluorescence microscopy (Fig. 4B) demonstrated that overexpression of wild-type PCBP1 significantly inhibited Mtb infection-induced Fe²⁺ elevation in THP-1 macrophages, whereas the PCBP1 mutant with an altered Fe-binding site lost this inhibitory effect. Similarly, cell death assays showed that overexpressing wild-type PCBP1 significantly reduced macrophage cell death, while the PCBP1 mutant was ineffective (Fig. 4C). Furthermore, WB results indicated that overexpressing wild-type PCBP1 significantly reduces the expression of the lipid peroxidation end product 4-HNE, an effect not observed with the PCBP1 mutant (Fig. 4D, Supplementary Fig. 9F).

These results highlight that PCBP1’s Fe-binding domain is essential for maintaining iron homeostasis and counteracting Mtb-driven ferroptosis. This discovery not only clarifies PCBP1’s mechanism in infection but also identifies its Fe-binding pocket as a potential therapeutic target. Future studies could explore small molecules that enhance the iron chaperone activity of PCBP1 to reinforce ferroptosis resistance, even though it remains unknown whether such stabilization would directly prevent Trim21-mediated PCBP1 degradation. Regardless, boosting the function of residual PCBP1 represents a complementary strategy to augment host defense against Mtb.

### PCBP1 orchestrates macrophage ferroptosis via GPX4, PTGS2, and HMOX1 modulation

**Fig. 5:**
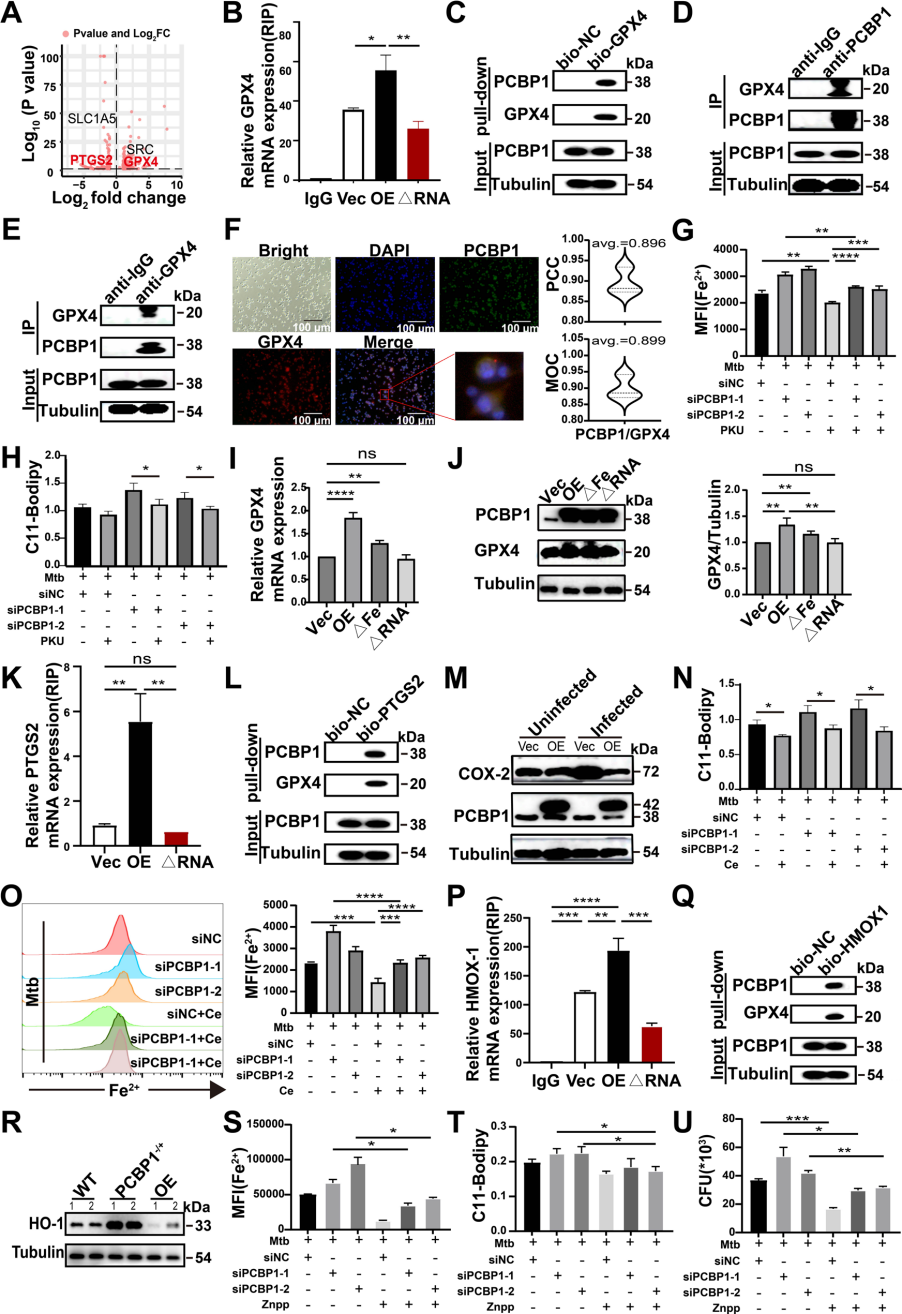
PCBP1 gates macrophage ferroptosis via tripartite control of GPX4, PTGS2, and HMOX1. (**A**) Volcano plot showing differentially expressed ferroptosis-related genes (*P* < 0.05) identified by PCBP1 RIP-Seq in Mtb-infected THP-1 macrophages, including 16 ferroptosis marker genes, eight ferroptosis-promoting genes, and 19 ferroptosis-inhibiting genes. (**B**) RIP assay showing that wild-type PCBP1 enhances binding to GPX4 mRNA, whereas the RNA-binding mutant (ΔRNA) reduces this interaction. (**C**) RNA pulldown assay demonstrating that biotinylated GPX4 mRNA probes specifically precipitate PCBP1 protein. (**D-E**) Co-IP assays showing direct interaction between PCBP1 and GPX4 protein. (**F**) Colocalization of PCBP1 and GPX4 within the cell. Scale bar, 100 μm. (G-H) GPX4 activator (PKU) treatment significantly reduces Fe²⁺ and lipid peroxidation levels in Mtb-infected macrophages, whereas PCBP1 knockdown attenuates this protective effect. (**I**) RT-qPCR analysis of GPX4 mRNA levels in THP-1-derived macrophages infected with H37Ra (MOI = 10, 24 h) and transfected with the indicated constructs: Empty vector (Vec), wild-type PCBP1 (WT), RNA-binding-deficient PCBP1 mutant (ΔRNA), or iron-binding-deficient PCBP1 mutant (ΔFe). (**J**) WB analysis of GPX4 protein expression under the same conditions described in (I). Tubulin served as a loading control. Blots are representative of three independent experiments. (**K**) RIP assay showing that wild-type PCBP1 enhances binding to PTGS2 mRNA, whereas the mutant PCBP1 reduces this interaction. (**L**) RNA pulldown assay confirming direct binding between PCBP1 and PTGS2 mRNA. (**M**) PCBP1 overexpression significantly reduces COX-2 expression in Mtb-infected macrophages. (**N-O**) COX-2 inhibitor (Ce) treatment significantly reduces Fe²⁺ and lipid peroxidation levels, whereas PCBP1 knockdown diminishes this protective effect. (**P-Q**) RIP and RNA pulldown assays confirming direct binding between PCBP1 and HMOX1 mRNA. (**R**) Inverse correlation between PCBP1 overexpression and HO-1 expression at both RNA and protein levels. (**S-U**) Znpp (HO-1 inhibitor) treatment alleviates elevated Fe²⁺ and lipid peroxidation levels in PCBP1-knockdown macrophages post-infection and reduces intracellular H37Ra bacterial load. Data are presented as mean ± SD. Statistical significance was determined by Student’s t-test or ANOVA (*p < 0.05, **p < 0.01, ***p < 0.001, ****p < 0.0001)

Using an integrated omics approach in Mtb-infected THP-1 macrophages, we identified PCBP1 as a central ferroptosis regulator. KEGG analysis of differentially expressed genes in PCBP1-overexpressing macrophages infected with H37Rv for 24 h highlighted ferroptosis pathway enrichment (Supplementary Fig. 4P, Supplementary Table 2). Subsequently, RIP-Seq revealed PCBP1 binding to numerous ferroptosis-related genes (*p* < 0.05), including key markers and regulators (Fig. 5A). These PCBP1-bound mRNAs were enriched in ROS responses and host antibacterial activities (Supplementary Fig. 5A, Supplementary Table 3).

By consolidating RIP, IP-MS, and transcriptomics data, we identified GPX4, PTGS2, and HMOX1 as the most likely direct downstream targets of PCBP1. RIP experiments confirmed PCBP1’s binding to the mRNAs of these targets (Supplementary Figs. 5B-E).

In our study (Sect. 3.3, Figs. 3A–B), we found that PCBP1 knockdown in THP-1 macrophages suppressed GPX4 expression and enzymatic activity, whereas PCBP1 overexpression enhanced them. RIP and IP-MS analyses revealed direct interactions between PCBP1 and GPX4 mRNA and protein. Constructing an RNA-binding site mutant of PCBP1 (ΔRNA: R40A/R124A/R306A) and performing RIP experiments showed that wild-type (WT) PCBP1 enhanced binding to GPX4 mRNA, whereas the mutant reduced this binding (Fig. 5B). RNA pulldown experiments confirmed that biotinylated GPX4 mRNA probes specifically precipitated PCBP1 protein (Fig. 5C). Co-IP experiments demonstrated a direct interaction between PCBP1 and GPX4 protein (Figs. 5D-E), with noticeable colocalization within the cell (Pearson’s correlation coefficient [PCC] = 0.896, Manders’ overlap coefficient [MOC] = 0.899; Fig. 5F).

To investigate PCBP1’s impact on GPX4 function, we treated Mtb-infected macrophages with a GPX4 activator (PKU). The results showed that PKU significantly reduced intracellular Fe²⁺ and lipid peroxidation levels (Figs. 5G-H, Supplementary Fig. 5F-G). However, this protective effect was markedly attenuated when PCBP1 was simultaneously knocked down (Figs. 5G-H). It indicates that PCBP1 modulates Mtb-induced ferroptosis by regulating GPX4 expression and function.

To delineate the molecular domain responsible for PCBP1-mediated GPX4 regulation, we employed structure-guided PCBP1 mutants defective in either RNA-binding or iron-binding. In Mtb-infected macrophages, overexpression of WT PCBP1 significantly elevated both GPX4 mRNA and protein levels, recapitulating its protective role. Strikingly, the RNA-binding-deficient mutant (ΔRNA) completely lost this ability to upregulate GPX4. Conversely, while the iron-binding-deficient mutant (ΔFe) also significantly enhanced GPX4 expression, its effect was slightly attenuated compared to WT PCBP1 (Figs. 5I-J). These results demonstrate that PCBP1 governs GPX4 expression primarily through its RNA-binding activity, likely by stabilizing GPX4 transcripts. The partial contribution of the iron-binding domain, albeit minor, suggests a potential auxiliary role, but it is dispensable for the core regulatory function observed here.

We found that Mtb infection significantly upregulates the expression of both PTGS2 mRNA and its encoded protein COX-2 in macrophages (Supplementary Figs. 5 H-I), implicating this pathway in infection-associated inflammation and lipid peroxidation. Given PCBP1’s established role in post-transcriptional regulation, we hypothesized that it might target PTGS2 for regulation. Initial RIP-seq data suggested an interaction, which was subsequently confirmed by targeted RIP assays: wild-type PCBP1 bound to PTGS2 mRNA, whereas an RNA-binding domain mutant (ΔRNA) exhibited markedly reduced binding (Fig. 5K). This direct interaction was further validated by RNA pulldown ((Fig. 5L). Functionally, PCBP1 overexpression effectively suppressed COX-2 protein levels (Fig. 5M).

To validate the role of PTGS2 in ferroptosis, we treated Mtb-infected macrophages with a COX-2 inhibitor (Ce). The results demonstrated that inhibiting COX-2 significantly reduced Fe²⁺ and lipid peroxidation levels (Figs. 5N-O, Supplementary Fig. 5J-K). However, when PCBP1 was simultaneously knocked down, the protective effects of Ce were diminished, with Fe²⁺ and lipid peroxidation levels increasing again (Figs. 5N-O). This indicates that PCBP1 regulates COX-2 expression by modulating the stability or translation of PTGS2 mRNA, thereby influencing ferroptosis.

HO-1, a key regulator of iron metabolism, is closely associated with ferroptosis [52]. RIP experiments and RNA pulldown assays confirmed direct binding between PCBP1 and HMOX1 mRNA (Figs. 5P-Q, Supplementary Fig. 5L). To investigate the role of HMOX1 in tuberculosis infection, we initially performed transcriptomic sequencing and found that HMOX1 mRNA levels were significantly upregulated in Mtb-infected macrophages (Supplementary Fig. 5M). This finding was corroborated in clinical samples: HMOX1 mRNA levels were also markedly elevated in peripheral blood mononuclear cells (PBMCs) from pulmonary tuberculosis patients compared to healthy controls (Supplementary Fig. 5N). To further confirm its functional relevance, we validated in the cellular model that the expression of its encoded protein, HO-1, was similarly induced by Mtb infection (Supplementary Figs. 5O-Q). Collectively, these data indicate that HMOX1/HO-1 is consistently activated in host cells during tuberculosis infection.

To further clarify the relationship between PCBP1 and HO-1, we examined HO-1 expression levels in PCBP1-overexpressing and PCBP1^−/+^ cells, finding an inverse correlation at both RNA and protein levels (Fig. 5R, Supplementary Figs. 5R-T). Rescue experiments demonstrated that treatment with Znpp (an HO-1 inhibitor) alleviated the increased Fe²⁺ and lipid peroxidation levels in PCBP1-knockdown macrophages following infection, while also reducing intracellular H37Ra bacterial load (Figs. 5S-U). These results suggest that PCBP1 suppresses HO-1-mediated ferroptosis by downregulating HO-1 expression, thereby enhancing macrophage bactericidal activity against Mtb.

In summary, PCBP1 inhibits ferroptosis induced by Mtb infection in macrophages through upregulating GPX4 expression and activity, while downregulating COX-2 and HO-1 expression. This study reveals the critical role of PCBP1 in ferroptosis regulation and provides a theoretical basis for its potential as a therapeutic target. The study uniquely demonstrated that PCBP1 interacts with both GPX4 mRNA and protein. This dual stabilization mechanism is a very distinctive finding that warrants deeper investigation.

### Trim21 impairs PCBP1-dependent ferroptosis restraint to facilitate Mtb survival in macrophages

**Fig. 6:**
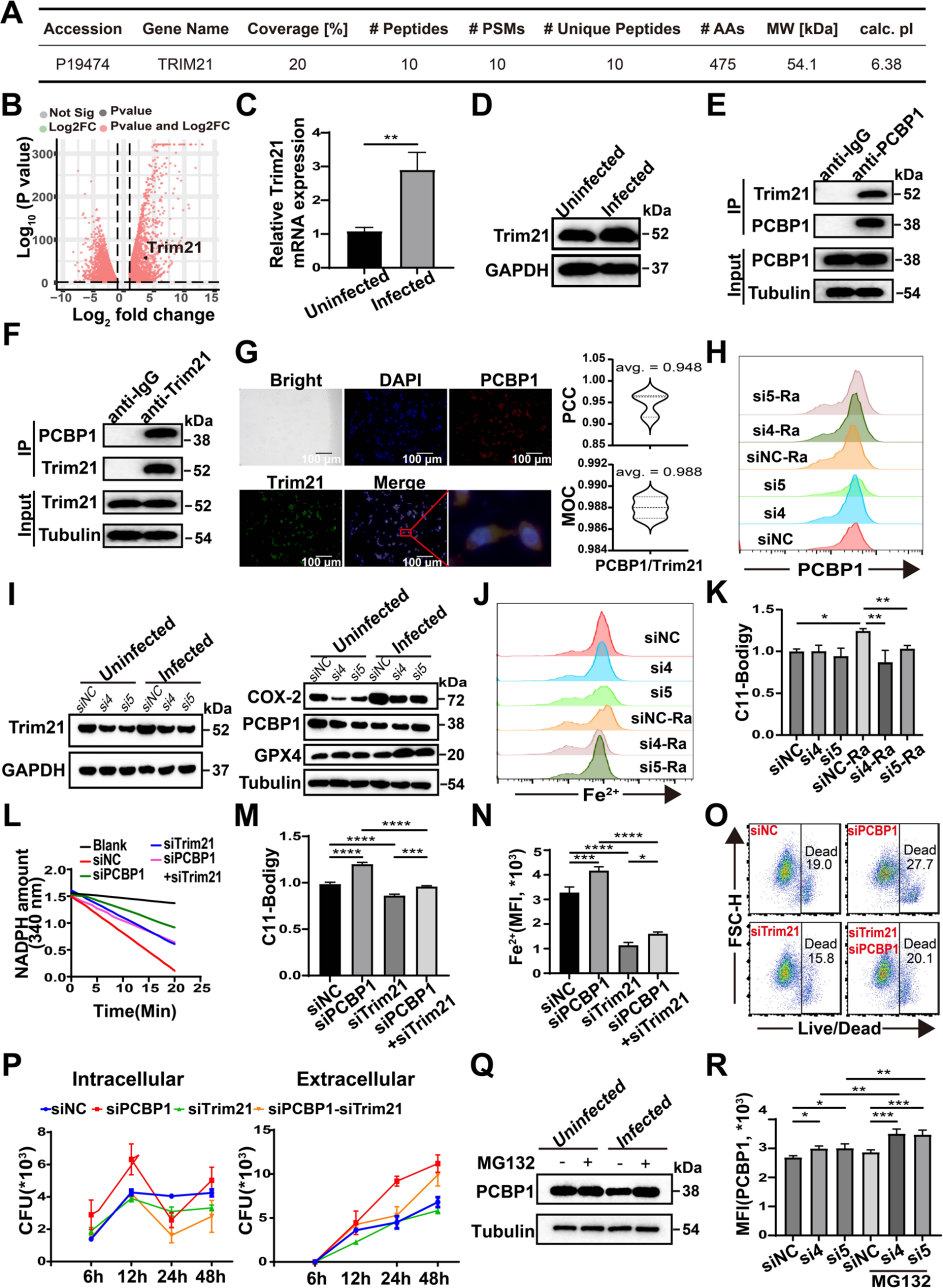
Trim21 Modulates Ferroptosis in Macrophages by Targeting PCBP1 During Mtb Infection. (**A**) Co-IP assay showing direct interaction between PCBP1 and Trim21 proteins in macrophages. (**B**) RNA-seq analysis reveals upregulation of Trim21 mRNA in Mtb-infected macrophages. (**C-D**) RT-qPCR and WB validation of Trim21 mRNA and protein upregulation in Mtb-infected macrophages. (**E-F**) Co-IP experiments confirming the direct interaction between Trim21 and PCBP1 proteins. (**G**) Confocal microscopy showing colocalization of Trim21 and PCBP1 within macrophages. Scale bar, 100 μm. (**H**) Trim21 knockdown increases PCBP1 protein levels in Mtb-infected macrophages. (**I**) Trim21 knockdown elevates GPX4 protein levels and reduces COX-2 expression in Mtb-infected macrophages. (**J-K**) Trim21 knockdown decreases Fe²⁺ (**J**) and lipid peroxidation levels (**K**) in Mtb-infected macrophages. (**L**) Trim21 knockdown rescues GPX4 activity reduction caused by PCBP1 knockdown. (**M-N**) Trim21 knockdown attenuates lipid peroxidation (**M**) and Fe²⁺ (**N**) levels elevated by PCBP1 knockdown. (**O**) Trim21 knockdown reduces macrophage cell death induced by PCBP1 knockdown. (**P**) Trim21 knockdown decreases intracellular and extracellular Mtb burden caused by PCBP1 knockdown, enhancing macrophage bactericidal activity. (**Q**) WB detection of PCBP1 protein levels in THP-1-differentiated macrophages under conditions of Mtb infection or non-infection, and MG132 treatment or not. (**R**) Flow cytometry analysis of PCBP1 protein expression in THP-1-derived macrophages with or without Trim21 knockdown and with or without MG132 treatment. Data are presented as mean ± SD. Statistical significance was determined by Student’s t-test or ANOVA (*p < 0.05, **p < 0.01, ***p < 0.001, ****p < 0.0001)

Previous studies have shown that PCBP1 expression is significantly downregulated in macrophages following Mtb infection [53–55], though the underlying mechanism remains unclear. IP-MS analysis revealed that PCBP1 directly interacts with Trim21 (Fig. 6A). RNA-seq analysis showed that Trim21 mRNA expression is significantly upregulated in Mtb-infected macrophages (Fig. 6B), which was confirmed by RT-qPCR and WB assays (Figs. 6C-D). Co-IP experiments demonstrated a direct interaction between Trim21 and PCBP1 proteins (Figs. 6E-F), and confocal microscopy showed noticeable colocalization of the two proteins within the cell (PCC = 0.948, MOC = 0.988; Fig. 6G). Trim21, an E3 ubiquitin ligase playing a critical role in the ubiquitin-proteasome protein degradation pathway, was hypothesized to regulate the downregulation of PCBP1 expression.

To explore Trim21’s role in PCBP1 downregulation in Mtb-infected macrophages, siRNA-based Trim21 knockdown models were established in THP-1-derived and RAW264.7 macrophages, and the efficiency was confirmed by RT-qPCR and WB (Supplementary Figs. 6 A-D). Experiments showed that Trim21 knockdown significantly increased PCBP1 expression (Fig. 6H) and GPX4 levels (Fig. 6I, Supplementary Fig. 9G) in Mtb-infected macrophages, while reducing COX-2 protein levels (Fig. 6I, Supplementary Fig. 9G), Fe²⁺ levels (Fig. 6J, Supplementary Figs. 6E-F), and lipid peroxidation (Fig. 6K, Supplementary Figs. 6G).

Further experiments indicated that PCBP1 knockdown decreases GPX4 activity and increases Fe²⁺ and lipid peroxidation levels, promoting ferroptosis. However, concurrent Trim21 knockdown attenuated the reduction in GPX4 activity caused by PCBP1 knockdown (Fig. 6L) and decreased the elevated levels of lipid peroxidation (Fig. 6M, Supplementary Fig. 6H) and Fe²⁺ (Fig. 6N, Supplementary Figs. 6I-J). Moreover, Trim21 knockdown reduced macrophage cell death induced by PCBP1 knockdown (Fig. 6O, Supplementary Figs. 6 K-L). Most importantly, Trim21 knockdown decreased the Mtb burden caused by PCBP1 knockdown (Fig. 6P, Supplementary Fig. 6M) and enhanced macrophage bactericidal activity. The proteasome inhibitor MG132 significantly reversed Mtb-induced downregulation of PCBP1 in THP-1-derived and RAW264.7 macrophages (Fig. 6Q, Supplementary Figs. 6N & 9 H). Critically, MG132 potentiates Trim21 knockdown-mediated upregulation of PCBP1 protein expression in THP-1-derived and RAW264.7 macrophages (Fig. 6R, Supplementary Fig. 6O).

In summary, Trim21 regulates ferroptosis in macrophages by modulating PCBP1 expression, and this study identifies Trim21 as a potential therapeutic target for TB.

### Optimized delivery and functional validation of saRNA in macrophages against Mtb

**Fig. 7:**
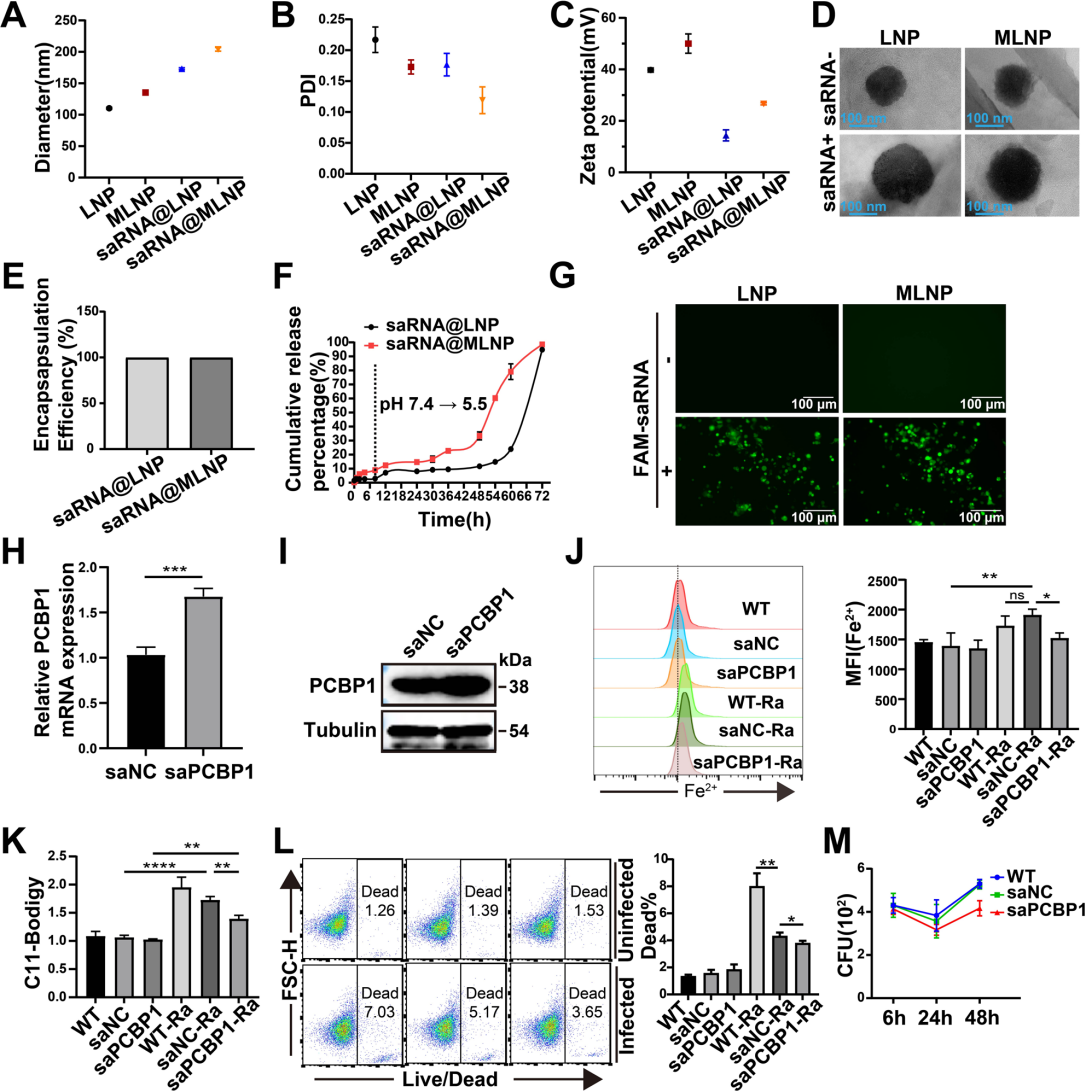
Characterization and functional evaluation of lipid nanoparticles in macrophages against Mtb. (**A**) Particle size analysis of MLNPs and unmodified LNPs, showing average diameters of 121.57 ± 2.954 nm and 131.1 ± 3.74 nm, respectively. (**B**) Polydispersity index (PDI) values of MLNPs and LNPs are both below 0.3, indicating uniform size distribution. (**C**) Zeta potential measurements of MLNPs and LNPs, showing values of 43.76 ± 2.59 mV and 39.92 ± 3.068 mV, respectively. (**D**) Electron microscopy images of MLNPs and LNPs, revealing spherical/elliptical morphology with smooth surfaces and average sizes of ~100 nm. (**E**) Encapsulation efficiency (EE) of saRNA in LNP and MLNP formulations, as determined by the Quant-iT^™^ RiboGreen^®^ RNA assay. (**F**) In vitro release kinetics of saRNA from LNP and MLNP in a pH-switch release assay. Nanoparticles were incubated in physiological buffer (pH 7.4) for 8 h, followed by transfer to acidic buffer (pH 5.5) to mimic the endo/lysosomal environment. Cumulative release was quantified using the RiboGreen^®^ assay. (**G**) Fluorescence microscopy images showing efficient cellular internalization of FAM-labeled saRNA@LNP and saRNA@MLNP by RAW264.7 macrophages after 24 h of incubation. Scale bar, 100 μm. (**H-I**) RT-qPCR and WB analysis showing upregulation of PCBP1 expression in RAW264.7 macrophages transfected with saPCBP1-loaded MLNPs. (J**-K**) saPCBP1 significantly inhibits Fe²⁺ levels and lipid peroxidation in infected RAW264.7 macrophages. (**L**) MLNP-delivered saPCBP1 reduces cell death in Mtb-infected RAW264.7 macrophages. (**M**) saPCBP1-loaded MLNPs reduce intracellular bacterial load and enhance macrophage antibacterial activity. Data are presented as mean ± SD. Statistical significance was determined by Student’s t-test or ANOVA (*p < 0.05, **p < 0.01, ***p < 0.001, ****p < 0.0001)

We successfully developed two cationic lipid nanoparticles (LNPs and mannose-modified MLNPs) using a modified film dispersion method. The lipid composition (DLin-MC3-DMA: DOPC: Chol: mannose-DMG-PEG/DMG-PEG = 50:38.5:10:1.5) included 50% DOTAP to enhance cationic properties. Both LNPs and MLNPs exhibited favorable physicochemical characteristics: MLNPs showed an average size of 121.57 ± 2.95 nm (LNPs: 131.1 ± 3.74 nm), PDI < 0.3, and zeta potentials of +43.76 ± 2.59 mV (MLNPs) and +39.92 ± 3.07 mV (LNPs), confirming stability and uniform morphology (Figs. 7A-C). The slightly more positive zeta potential of MLNPs is likely due to the presence of positively charged amine groups in the linker of the mannose-conjugated PEG lipid. Electron microscopy revealed spherical/elliptical structures with smooth surfaces (~100 nm, Fig. 7D).

For saRNA encapsulation, a 1:20 mass ratio and 3:1 volume ratio in citrate buffer yielded stable complexes. Post-loading, saRNA@MLNPs and saRNA@LNPs exhibited increased sizes (204.1 ± 1.69 nm and 169.74 ± 3.94 nm, respectively) with PDI < 0.2 and reduced zeta potentials (+26.65 ± 0.21 mV and +19.27 ± 2.63 mV, Figs. 7A–C). High-density internal regions observed via electron microscopy confirmed successful encapsulation (Fig. 7D).

Pharmaceutical characterization confirmed the excellent performance of our nanoparticles. The encapsulation efficiency of saRNA exceeded 99% for both LNP and MLNP formulations (Fig. 7E). In vitro release kinetics under physiological conditions (pH 7.4) demonstrated remarkable stability, with less than 10% cumulative release over 8 h. Upon shifting to an acidic environment (pH 5.5) mimicking endo/lysosomes, a rapid and sustained release was triggered, achieving near-complete release (> 90%) within 72 h (Fig. 7F). This pH-responsive behavior is mediated by the MC3. At neutral pH, MC3 remains largely neutral, allowing stable encapsulation of saRNA. After cellular uptake and exposure to the acidic endosomal pH (5.5–6.0), the tertiary amine groups of MC3 become protonated, generating a positive surface charge. This charge switch promotes electrostatic interaction with the negatively charged endosomal membrane, leading to lipid bilayer disruption and subsequent release of the saRNA cargo into the cytosol [56, 57]. This mechanism, known as charge-mediated membrane destabilization, is well established for MC3-based lipid nanoparticles. These data establish our nanoparticles as a stable, pH-responsive delivery platform capable of protecting and controllably releasing saRNA.

To validate the functionality of the nano-delivery system, we first confirmed via fluorescence microscopy that FAM-labeled saRNA@LNP/MLNP was efficiently internalized by RAW264.7 macrophages (Fig. 7G). Subsequently, we encapsulated saRNAs targeting murine PCBP1 into MLNPs and demonstrated that this strategy significantly upregulated PCBP1 expression at both transcriptional and translational levels, with co-encapsulation of multiple saRNA sequences yielding a superior effect (Figs. 7H-I, Supplementary Fig. 9I). Furthermore, in the Mtb infection model, saPCBP1@MLNP treatment exhibited multifaceted protective effects by effectively suppressing iron overload (Fig. 7J) and lipid peroxidation (Fig. 7K), and significantly reducing infection-induced cell death (Fig. 7L), indicating a restoration of cellular redox homeostasis. Ultimately, this series of beneficial modulations translated into potent antibacterial function, as evidenced by a significant reduction in intracellular bacterial load (Fig. 7M).

This study establishes a robust platform for saRNA delivery via cationic LNPs/MLNPs, demonstrating their utility in gene regulation and host-directed antimicrobial therapy. The optimized formulations exhibit excellent uniformity, stability, and bioactivity, supporting their potential for anti-tuberculosis applications.

### Lung-targeted lipid nanoparticles restore PCBP1 to inhibit ferroptosis and eradicate Mtb in vivo

**Fig. 8:**
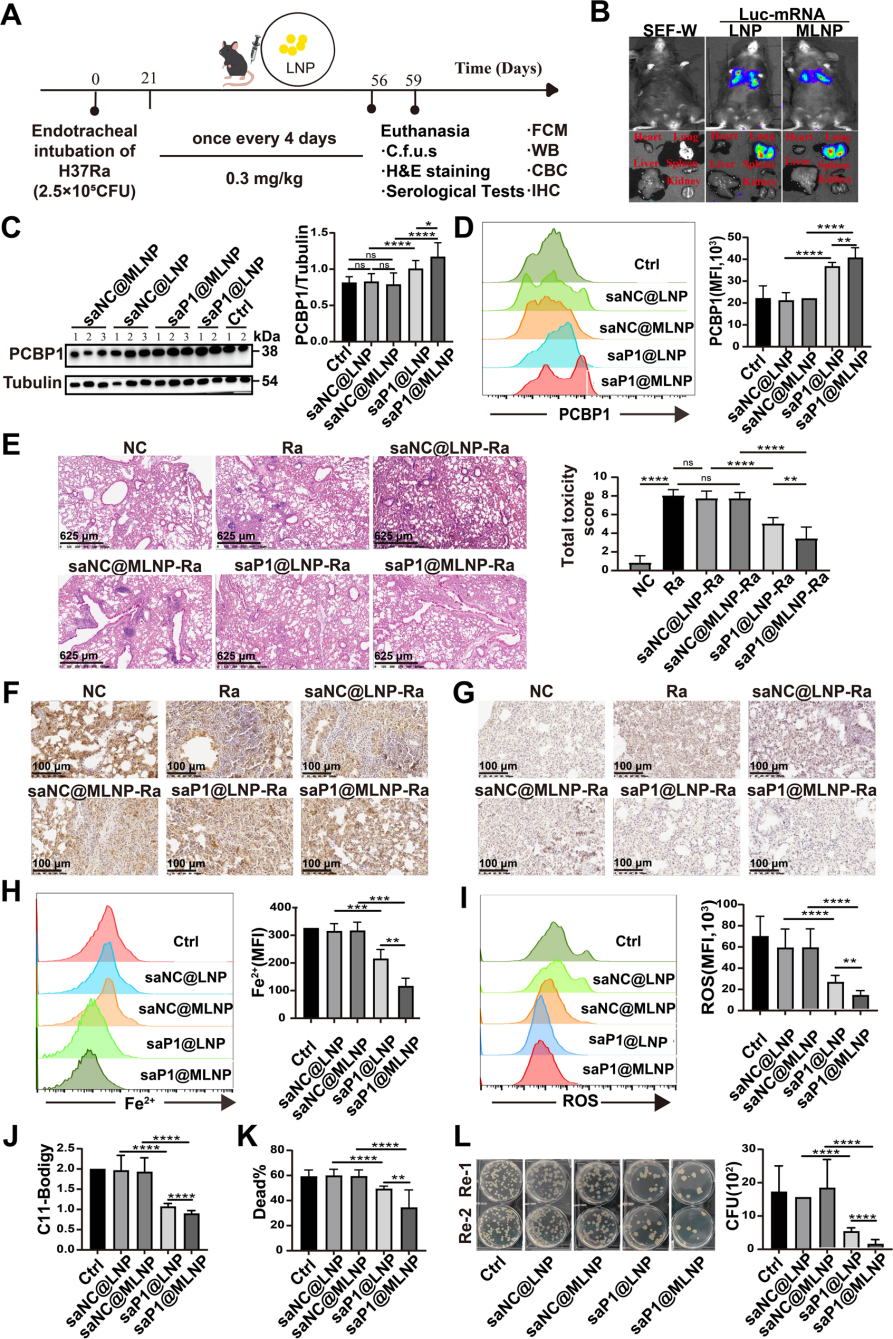
PCBP1-activating Lipid Nanoparticles Reduce Bacterial Load and Pathological Damage in a TB Mouse Model. (**A**) Experiment timeline. (**B**) In vivo imaging of Luc-mRNA-loaded LNPs and MLNPs in C57BL/6J mice, showing strong bioluminescent signals in the lungs 6 h post-injection. (**C**) WB analysis of PCBP1 protein expression in mouse lung tissues treated with saPCBP1@LNP or saPCBP1@MLNP. (**D**) Flow cytometry analysis of PCBP1 protein expression in mouse lung tissues, showing increased fluorescence intensity in saPCBP1-treated groups. (**E**) PCBP1-activating nanoparticles mitigate pulmonary histopathology in Mtb-infected mice. (Left) Representative H&E images. (Right) Semi-quantitative assessment of lung injury (scored 0–9 for inflammation, alveolar damage, and granulomas). (**F**) Immunohistochemistry showing GPX4 protein expression in mouse lung tissues. (**G**) Immunohistochemistry showing 4-HNE level in mouse lung tissues. (**H-K**) Analysis of Fe²⁺ levels (**H**), ROS levels (I), lipid peroxidation (**J**) and cell death (**K**) in mouse lung tissues. (**L**) CFU assay showing reduced bacterial load in lung tissues of saPCBP1-treated mice. Data are presented as mean ± SD. Statistical significance was determined by Student’s t-test or ANOVA (*p < 0.05, **p < 0.01, ***p < 0.001, ****p < 0.0001)

To evaluate the therapeutic potential of targeted PCBP1 activation in tuberculous pneumonia, we established the research system outlined in Fig. 8A. Mice were randomized into five groups (*n* = 10): sterile enzyme-free water (SEF-W), saNC@LNP, saNC@MLNP, saPCBP1@LNP, and saPCBP1@MLNP. C57BL/6J mice were infected with H37Ra (2.5 × 10⁵ CFU per mouse) by orotracheal instillation. Starting from day 21 post-infection, the mice were intravenously injected with lipid-nanoparticle-encapsulated saRNA (0.3 mg/kg) every 4 days. Treatment continued until day 56, after which the mice were sacrificed on day 59 for sample collection and subsequent experiments.

To assess the targeting efficiency of our delivery system, Luc-mRNA-loaded LNPs/MLNPs were intravenously administered to C57BL/6J mice. Non-invasive in vivo bioluminescence imaging at 6 h post-administration showed predominant lung accumulation of the nanocarriers. Meanwhile, bioluminescent signals were barely detectable in other major organs including the liver, spleen, kidney and heart, indicating minimal off-target distribution and strong lung-targeting capability of our delivery system (Fig. 8B). Safety assessment of the system supported its potential for translation. In healthy mice, administration of empty LNPs/MLNPs did not induce significant alterations in major organ-to-body weight ratios (Supplementary Figs. 7 A-E), serum liver function markers (Supplementary Figs. 7 F-I), or routine blood parameters (Supplementary Figs. 7 J-M). General morphology and histopathological examination of the lungs, liver, spleen, and kidneys revealed no toxicity-associated pathological changes (Supplementary Figs. 7 N-O). Furthermore, levels of the key pro-inflammatory cytokines (IL-1β, IL-6, and TNF-α) in homogenates of the lung, liver, spleen, and kidney remained unchanged following nanoparticle treatment (Supplementary Figs. 7P-R for respective data). Collectively, these data indicate that the therapeutic platform exhibits a favorable biocompatibility profile.

In the therapeutic model, WB and flow cytometry analyses showed that saPCBP1@LNP and saPCBP1@MLNP significantly activated PCBP1 protein expression compared to controls, with saPCBP1@MLNP showing the strongest effect (Figs. 8C-D). These findings demonstrate the ability of our targeted delivery system to achieve efficient functional activation of the target gene within the disease microenvironment.

Further analysis of organ indices (heart, lung, liver, spleen, and kidney) showed no significant differences between groups (Supplementary Figs. 8 A-E), indicating that saPCBP1-activating nanoparticles do not alter organ weight ratios. Serum biochemistry results showed no significant changes in AST and ALT levels in saPCBP1-treated mice compared with the non-specific activation groups (saNC@LNP and saNC@MLNP) (Supplementary Figs. 8 F-G), confirming no hepatic toxicity.

Blood analyses revealed no significant differences in white blood cell counts between groups (Supplementary Fig. 8H), but saPCBP1-treated mice showed significantly lower neutrophil and monocyte percentages, especially in the saPCBP1@MLNP group (Supplementary Figs. 8I-J), with a trend toward higher lymphocyte percentages (Supplementary Fig. 8K). These results suggest that PCBP1 activation may modulate immune cell distribution or function to mitigate Mtb-induced inflammation.

Morphological examination (Supplementary Fig. 8L) and H&E staining (Fig. 8E) of lung tissues revealed a marked therapeutic effect. Mice in non-specific activation groups (saNC@LNP and saNC@MLNP) exhibited typical tuberculous pathological alterations, including diffuse inflammatory cell infiltration, extensive disruption of alveolar architecture, and numerous granulomatous nodules. In contrast, these pathological changes were substantially alleviated in the saPCBP1-treated groups (Fig. 8E, left). Quantitative analysis based on a standardized scoring system demonstrated that saPCBP1@MLNP treatment significantly reduced the total pathological injury score, outperforming the saPCBP1@LNP formulation (Fig. 8E, right).

To elucidate the molecular mechanism of the therapeutic effect, we measured the indicators related to ferroptosis in lung tissue. Immunohistochemistry revealed that saPCBP1 treatment restored GPX4 expression (Fig. 8F) and reduced 4-HNE levels (Fig. 8G) in the lung tissues of Mtb-infected mice. What’s more, saPCBP1 activation lowered Fe²⁺, ROS, lipid peroxidation levels and cell death, with saPCBP1@MLNP showing the most potent effects (Figs. 8H-K). These findings delineate a coherent pathogenic cascade: restoration of PCBP1 leads to inhibition of ferroptosis, which in turn alleviates tissue damage.

Ultimately, the gold standard assay of CFU counting confirmed the potent antibacterial efficacy of the treatment. CFU assays showed that saPCBP1-activating nanoparticles significantly reduced bacterial loads in lung tissues, with saPCBP1@MLNP being the most effective (Fig. 8L). These results highlight the therapeutic potential of PCBP1-activating lipid nanoparticles, particularly mannosylated ones, in reducing bacterial burden and pathological damage in tuberculosis infection. saPCBP1@MLNPs showed better efficacy compared with saPCBP1@LNPs, which strongly supports the superiority of the mannose-targeting strategy and the success of the “active targeting” approach.

## Discussion

This study elucidates the pivotal role of PCBP1 in counteracting Mtb-induced macrophage ferroptosis and identifies its regulatory mechanisms, offering novel therapeutic avenues for TB. Our findings reveal that Mtb infection downregulates PCBP1 expression in macrophages via Trim21-mediated ubiquitin-proteasomal degradation, leading to iron overload, lipid peroxidation accumulation, and GPX4 suppression, ultimately triggering ferroptosis. These results align with prior studies demonstrating that pathogens exploit host iron metabolism to promote infection [58]. However, our work uniquely establishes PCBP1 as a critical checkpoint in Mtb pathogenesis by integrating iron homeostasis, redox balance, and ferroptosis regulation—a mechanism not previously reported in TB research.

PCBP1 stabilizes GPX4 mRNA and protein, enhancing GPX4’s enzyme activity to inhibit lipid peroxidation and ferroptosis, which is consistent with GPX4’s established role as a core ferroptosis suppressor [59, 60]. Structural analysis suggests that PCBP1 may bind GPX4 mRNA through its KH domains, particularly targeting the 3’ untranslated region (3’UTR) to prevent RNA degradation, akin to the role of HuR in stabilizing p21 mRNA [61, 62]. This interaction likely involves adenosine/uracil-rich elements (AREs) in GPX4 mRNA, a common feature of PCBP1-targeted transcripts. Notably, PCBP1 also physically interacts with the GPX4 protein, potentially shielding it from ubiquitination (Fig. 6D–F). This dual stabilization mechanism contrasts with the sole RNA-binding function of other ferroptosis suppressors like FSP1 [43], highlighting PCBP1’s unique multifunctionality. Beyond GPX4, PCBP1 binds to PTGS2 and HMOX1 mRNA, inhibiting COX-2 translation and possibly accelerating HMOX1 transcript degradation, possibly regulating iron metabolism and redox balance [60]. Still, the present study did not examine mRNA degradation; therefore, in the absence of direct evidence of mRNA degradation, the present study suggests that PCBP1 overexpression was associated with reduced HMOX1 mRNA levels, suggesting a role in promoting its degradation. The suppression of COX-2 aligns with recent evidence that prostaglandin E2 exacerbates Mtb survival by dampening autophagy [63], while HMOX1 downregulation prevents excessive heme-derived iron release—a key driver of ferroptosis [64]. This dual regulatory function makes PCBP1 a multifunctional ferroptosis inhibitor, contrasting with its previously reported roles in cancer models [18, 23]. These findings not only expand PCBP1’s role in ferroptosis regulation but also offer fresh insights into TB’s molecular mechanisms. The ability of PCBP1 to directly interact with and stabilize GPX4 mRNA while simultaneously regulating the translation of PTGS2 and degradation of HMOX1 mRNA underscores its significance in maintaining cellular redox balance and preventing ferroptosis. This multifaceted regulatory mechanism positions PCBP1 as a central node in the complex network of iron and redox homeostasis within macrophages during Mtb infection, highlighting its potential as a therapeutic target for TB.

Of note, the present study was not designed to determine how PCBP1 affected those three pathways. PCBP1 coordinates a network of post-transcriptional regulatory activities that together determine macrophage resistance to ferroptosis. Its effects on GPX4, PTGS2, and HMOX1 are mechanistically linked, working in concert to modulate the cell’s oxidative and iron homeostasis environment [65, 66]. GPX4 is essential for neutralizing lipid peroxides, directly halting the lipid-based oxidative damage that signals ferroptosis. PCBP1 stabilizes GPX4 mRNA or protein, maintaining sufficient GPX4 activity and robust antioxidant defenses [65–67]. PTGS2 is induced under oxidative stress and functions as both a pro-inflammatory enzyme and a cellular stress marker. PCBP1 may enhance PTGS2 expression, creating a feedback loop that signals the cell’s need to adapt to oxidative pressures and modulate immune responses [68–70]. HMOX1 liberates iron from heme, increasing the pool of potentially toxic free iron that potentiates ferroptosis. By promoting HMOX1 degradation or destabilization, PCBP1 keeps intracellular iron levels in check, reducing the substrate available for lipid peroxidation [65, 71]. By stabilizing GPX4, PCBP1 provides direct antioxidant protection. By suppressing PTGS2, it limits pro-inflammatory lipid mediator production. By degrading HMOX1, it preemptively removes the iron supply that propels lipid peroxidation. These pathways reinforce each other, each reducing the stress or toxic load that would otherwise challenge the others [65, 66, 71]. If PCBP1 is lost or diminished, GPX4 drops (reducing antioxidant defense), HMOX1 rises (increasing iron), and stress signaling via PTGS2 could be dysregulated. This creates a cellular environment primed for ferroptosis, highlighting why these actions are functionally interconnected [65, 66]. PCBP1 binds to the 3’-untranslated regions (3’-UTRs) of mRNAs encoding key proteins involved in ferroptosis defense, fine-tuning their stability and translation [66, 72]. Taken together, it can be summarized that activating PCBP1 stabilizes GPX4, inhibits PTGS2, and degrades HMOX1. Inhibiting HMOX1 reduces the availability of iron, while stabilizing GPX4 enhances antioxidant capacity. Therefore, activating PCBP1 limits the fuel and the environment, thereby suppressing ferroptosis. Future studies should investigate the interplay among these pathways in more detail.

Additionally, we discovered that Trim21, as an E3 ubiquitin ligase, drives PCBP1 degradation, enhancing our understanding of host-pathogen interactions. This indicates Mtb hijacks host ubiquitination mechanisms to disrupt iron metabolism, similar to its manipulation of ferritinophagy [15, 73]. Mtb upregulates TRIM21 in host macrophages primarily by activating the p38 and AKT1 signaling pathways. Upon infection, Mtb increases both the mRNA and protein levels of TRIM21 in a time-dependent manner. This upregulation has downstream effects: TRIM21 acts as an E3 ubiquitin ligase that mediates the proteasomal degradation of HERC2, a protein that normally limits the levels of NCOA4. With more TRIM21, HERC2 gets degraded, resulting in higher NCOA4 levels. The increase in NCOA4, in turn, promotes ferritinophagy, making iron more available to Mtb for its intracellular growth [15]. Mtb could also elevate Trim21 expression potentially through TLR2/4-mediated NF-κB activation [15], mirroring Salmonella’s exploitation of host ubiquitination to disrupt iron transporters [74]. Targeting this axis with proteasome inhibitors (e.g., bortezomib) [75] could synergize with PCBP1 activation strategies, offering a dual-pronged approach against Mtb’s immune evasion. Trim21’s identification not only broadens our comprehension of host-pathogen interactions but also presents a potential TB therapeutic target. The role of Trim21 in targeting PCBP1 for degradation reveals a novel strategy employed by Mtb to evade host immune defenses and promote its own survival. By modulating the host’s ubiquitin-proteasome system to degrade key regulatory proteins like PCBP1, Mtb can effectively dysregulate iron homeostasis and redox balance within macrophages, creating a more favorable environment for its intracellular replication. This discovery adds to the growing body of evidence demonstrating how pathogens can exploit host cellular processes for their benefit, and it opens new avenues for the development of therapeutic interventions aimed at preserving PCBP1 function and preventing ferroptosis during Mtb infection. Nevertheless, it must be highlighted that although our data show that Trim21 interacts with PCBP1 and that Trim21 knockdown reverses PCBP1 downregulation, given that Trim21 is a known E3 ubiquitin ligase, these findings strongly suggest that Trim21 may mediate PCBP1 degradation. Further experiments (such as the detection of PCBP1 ubiquitination) are required to directly confirm the involvement of the ubiquitin-proteasome pathway in this process.

In addition to the mechanistic insights discussed above, we observed an interesting organ-specific pattern of PCBP1 expression. While PCBP1 expression was significantly downregulated in the lungs of Mtb-infected mice, it remained unchanged in the spleen. This organ-specific difference likely reflects distinct pathophysiological microenvironments and infection dynamics. First, the lung is the primary site of Mtb infection and pathology, where bacterial burden and host–pathogen interactions are most intense. Sustained exposure of alveolar and interstitial macrophages to Mtb drives robust Trim21 upregulation and subsequent PCBP1 degradation. In contrast, the spleen is a secondary lymphoid organ that receives a significantly lower and more intermittent bacterial load via hematogenous dissemination, particularly in the H37Ra infection model used for our therapeutic studies. Second, macrophage subsets exhibit profound heterogeneity across organs: lung macrophages are potentially more permissive to Mtb-induced manipulation of the ubiquitin–proteasome system, whereas splenic macrophages may be less susceptible due to differences in receptor expression (e.g., mannose receptor, TLR2) or intracellular signaling cascades. Third, the kinetics of bacterial dissemination and immune activation differ; at the time points examined, the spleen may not have reached the threshold of infection intensity required to trigger a detectable PCBP1 reduction. Future studies could systematically compare the temporal and spatial dynamics of PCBP1 degradation across multiple organs to fully elucidate this tissue-specific regulation.

Another critical question that arises from our study is why we chose to activate PCBP1 rather than inhibit its upstream E3 ligase Trim21. Several considerations guided this decision. First, PCBP1 is the central effector of the anti-ferroptosis program; restoring its expression directly reinstates the entire protective networks. In contrast, Trim21 may have multiple substrates beyond PCBP1, and systemically inhibiting it could cause unpredictable off-target effects in host immunity. Second, since Mtb infection causes a loss of PCBP1 function, employing a gain-of-function restoration (via RNAa) is a more direct and precise strategy than relying on the loss-of-function inhibition of Trim21. Third, RNAa-based therapies have demonstrated clinical feasibility (e.g., MTL-CEBPA), whereas developing specific small-molecule inhibitors for E3 ligases remains pharmacologically challenging. Nonetheless, targeting Trim21 could serve as a valuable complementary approach, and future studies may explore combination strategies.

Furthermore, saRNA was selected over mRNA because it activates endogenous transcription, providing a more stable and sustained PCBP1 expression which is uniquely suitable for managing chronic diseases like TB, whereas mRNA typically offers only transient protein synthesis. Moreover, saRNA has established clinical precedents: MTL-CEBPA has entered human trials for liver cancer [76], and RAG-01 recently received FDA Fast Track designation [77]. This translational feasibility supports our choice of saRNA for developing the therapeutic platform. We developed an MLNP-based saRNA delivery system, demonstrating PCBP1 activation’s therapeutic potential. This system achieved lung-targeted delivery. By restoring PCBP1 expression, it restored macrophages’ bactericidal ability, which is the first successful application of PCBP1-based therapy to improve the fight against Mtb infection. Furthermore, it overcame conventional TB therapy limitations like hepatic sequestration and poor macrophage targeting [78, 79]. A 50% DOTAP formulation boosted pulmonary accumulation, while mannosylation ensured macrophage specificity, synergistically reducing bacterial load and inflammation. These findings build on emerging saRNA-based therapies [29, 33] and lipid nanoparticle technologies [80, 81], but they are the first to apply saRNA to TB treatment. The successful application of MLNP-based saRNA delivery system represents a significant advancement in the development of host-directed therapies for TB. This approach not only addresses the limitations of traditional TB treatments but also leverages the precision and specificity of nucleic acid-based therapies to enhance the host’s innate immune response against Mtb. The ability to specifically target macrophages within the lungs ensures that the therapeutic agent is delivered directly to the site of infection, maximizing its efficacy while minimizing potential side effects. Furthermore, this study highlights the promise of combining advanced delivery systems with gene regulatory strategies to develop innovative treatments for infectious diseases, potentially revolutionizing how we approach TB and other complex infections.

Although our peripheral blood analysis revealed that saPCBP1@MLNP treatment significantly reduced neutrophil and monocyte percentages (Supplementary Figs. 8I–J) and showed a trend toward higher lymphocyte percentages (Supplementary Fig. 8K), we acknowledge that the present study did not comprehensively characterize local immune responses in the lung microenvironment. Nevertheless, based on the established role of ferroptosis in promoting inflammation through damage-associated molecular pattern (DAMP) release [82, 83], we hypothesize that targeted PCBP1 activation limits the efflux of pro-inflammatory DAMPs from ferroptotic macrophages. Consequently, this mechanism would dampen excessive pulmonary inflammation and preserve the local functional niche for T cells. This hypothesis is consistent with our observations of reduced lung pathology (Fig. 8E) and the trend of increased lymphocytes in the peripheral blood (Supplementary Fig. 8K), which together indicate a shift from a hyper-inflammatory state to an effective antimicrobial immune response. A comprehensive analysis of the dynamic shifts within the lung immune compartment—specifically the repolarization of alveolar macrophages and the infiltration of effector T cell subsets—alongside local cytokine networks will be a critical focus of our future studies. These prospective investigations will be essential to fully elucidate the broader immunomodulatory consequences of this PCBP1-based host-directed therapy against tuberculosis.

Despite revealing PCBP1’s significant role in TB, further research is needed to explore its interactions with other ferroptosis regulators and its functions in different host cells. Optimizing nanoparticle formulations to enhance delivery efficiency and biocompatibility will also be crucial for future research [84]. These efforts will solidify the theoretical foundation for precision TB therapy. Future studies should also consider the potential impact of host genetic variability on PCBP1 expression and function, as well as the influence of different Mtb strains on this regulatory axis. Additionally, investigating the role of PCBP1 in the context of latent TB infection and reactivation could provide valuable insights into the long-term host-pathogen dynamics and inform strategies for preventing disease progression. Furthermore, exploring the combination of PCBP1 activation with existing anti-TB drugs may reveal synergistic effects that could enhance treatment outcomes and shorten therapy duration. saRNA therapy is highly innovative, but its long-term application in infectious diseases may face challenges. saRNA vaccines can trigger strong innate and adaptive immune responses, leading to side effects or reduced efficacy upon repeated administration. The immunogenic profile is influenced by saRNA design, any double-stranded RNA contaminants, and the delivery platform [85–87]. Rapid degradation and strong immune activation may necessitate frequent dosing to maintain therapeutic effect, which can increase risks and reduce compliance [85–87]. saRNA is more fragile than standard mRNA, making it susceptible to degradation before reaching the target cells. Effective protection and delivery systems are thus essential for sustained activity [88, 89]. Future studies need to address potential challenges of saRNA-based therapies, such as immune responses upon prolonged use, and the adaptability of different administration routes (e.g., inhalation). Furthermore, although the present study was performed primarily in macrophages, the bioinformatics analysis indicated a potential involvement of B cells, which should be investigated in future studies.

## Conclusion

In summary, our study uncovers an unrecognized axis where Mtb degrades PCBP1 via Trim21 to drive ferroptosis and evade immunity. By clarifying PCBP1’s dual role in stabilizing GPX4 and inhibiting COX-2/HMOX1, we provide insights into iron metabolism dysregulation in TB. Our work breaks new ground by identifying the degradation of PCBP1 by Mtb as a key driver of ferroptosis and immune evasion. The activation of PCBP1 via MLNP-delivered saRNA establishes a proof-of-concept for RNA-based host-directed therapy. Future research should investigate PCBP1’s interactions with other ferroptosis regulators and refine nanoparticle formulations for clinical translation. These discoveries not only enhance our understanding of TB pathogenesis but also pave the way for innovative host-directed therapies against drug-resistant infections.

## Electronic Supplementary Material

Supplementary Material 1

Supplementary Material 2

Supplementary Material 3

## Data Availability

The data for this study [SRR11038989-SRR11038995] are available in the NCBI Sequence Read Archive (SRA). GSE203261 can be found in the GEO dataset. Any additional data supporting the manuscript can be obtained from the corresponding author upon request.

## Abbreviations

Mtb: Mycobacterium tuberculosis
TB: Tuberculosis
PCBP1: Poly(C)-binding protein 1
RNAa: RNA activation
MLNPs: Mannosylated lipid nanoparticles
saRNAs: Small activating RNAs
WHO: World Health Organization
LNPs: Lipid nanoparticles
IRB: Institutional Review Board
FBS: Fetal bovine serum
MOI: Multiplicity of infection
Ad5: Adenovirus type 5
WB: Western blot
Co-IP: Co-immunoprecipitation
RIP: RNA immunoprecipitation
GPx: Glutathione peroxidase
FSC: Forward scatter
SSC: Side scatter
MFI: Mean fluorescence intensity
CFU: Colony-forming units (CFU)
DOTAP: 1, 2-dioleoyl-3-trimethylammonium-propane
TEM: Transmission electron microscopy
TBS: Tris-buffered saline
CBC: Complete blood count
WBC: White blood cell count
WT: Wild-type
RIN: RNA integrity number
GO: Gene ontology
DEGs: Differentially expressed genes
KEGG: Kyoto encyclopedia of genes and genomes
GSEA: Gene set enrichment analysis
PBMCs: Peripheral blood mononuclear cells
H-PMs: Human primary macrophages
3'-UTRs: 3’-untranslated regions
