# Supplementary material for: Mycobacterium tuberculosis-induced PCBP1 degradation drives macrophage ferroptosis to promote infection: a lung-macrophage-targeted RNAa nanotherapy
Source: J Nanobiotechnology. 2026 May 21;24:668. doi: 10.1186/s12951-026-04569-x (PMC13377778; doi:10.1186/s12951-026-04569-x)
Supplement: Supplementary file 1 — Supplementary Material 1 [file 12951_2026_4569_MOESM1_ESM.docx]

# Supplementary materials

**Supplementary Table 1. Criteria for semi-quantitative histopathological scoring of lung tissues.**

| **Score** | **Inflammatory Cell**  **Infiltration** | **Alveolar Structural**  **Damage** | **Granuloma**  **Formation** |
| --- | --- | --- | --- |
| 0 | None or minimal | Normal, intact architecture | None |
| 1 | Mild, focal (<25% of field) | Mild septal thickening/  rare fusion | 1-2 small, ill-defined |
| 2 | Moderate, multifocal (25-50%) | Obvious thickening & fusion/  collapse | 3-5 well-defined |
| 3 | Severe, diffuse (>50%) | Severe distortion,  consolidation | Numerous or large, confluent |

**Supplementary Table 2. KEGG pathway enrichment analysis of differentially expressed genes (DEGs) in PCBP1-overexpressing macrophages infected with H37Rv.**

**Supplementary Table 3. Gene Ontology (GO) enrichment analysis of PCBP1-bound mRNAs identified by RIP-Seq in H37Rv-infected macrophages.**

**
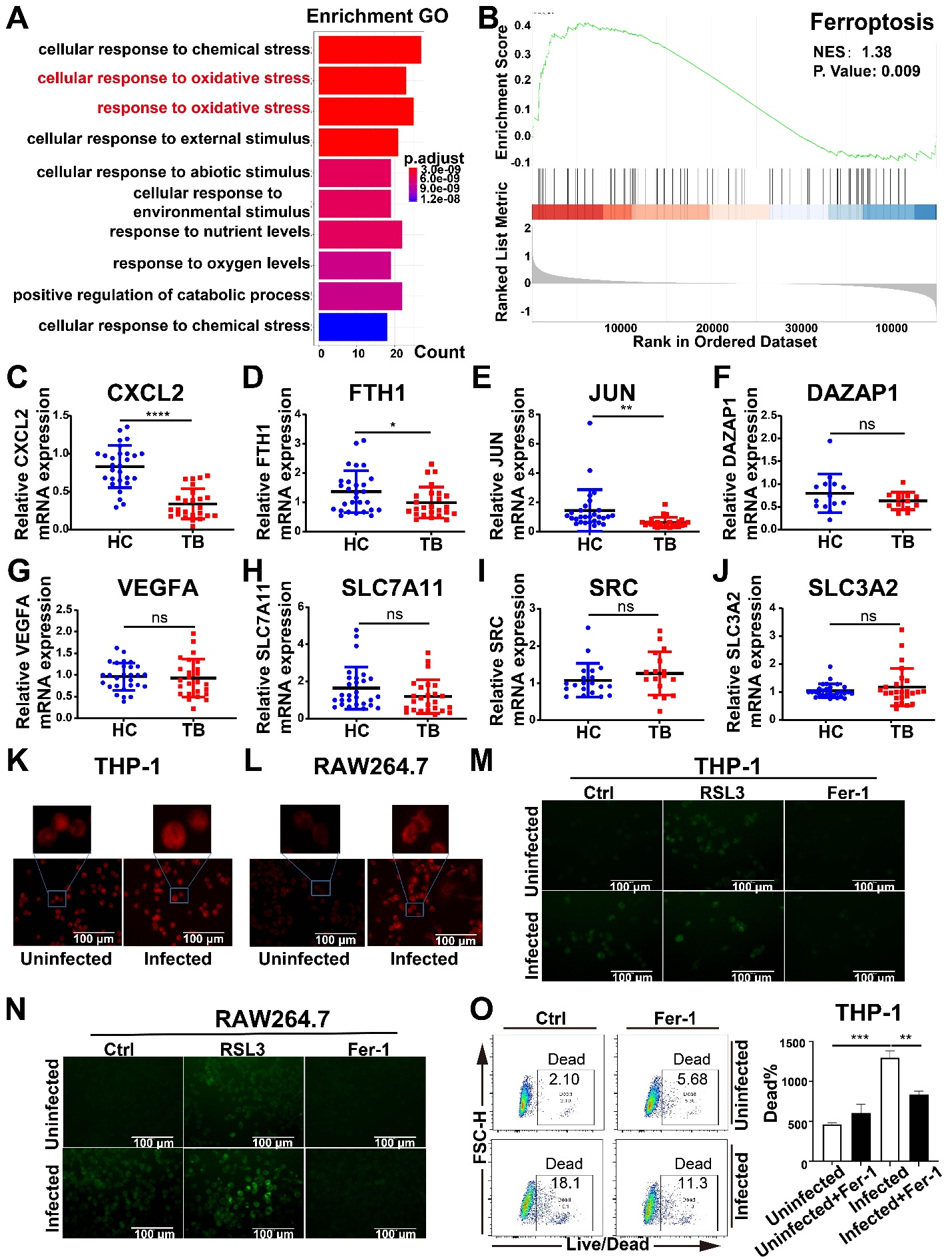
**

**Supplementary Figure 1. Validation of ferroptosis-related molecules and phenotypes in Mtb-infected PBMCs and macrophages.**

1. GO enrichment analysis of DEGs derived from bulk RNA-seq of H37Rv-infected macrophages, highlighting associations with oxidative stress response and peroxisome function. (B) GSEA confirms activation of ferroptosis signaling in infected macrophages (Normalized Enrichment Score [NES]=1.38, false discovery rate [FDR] q < 0.01). Leading-edge genes driving the enrichment are highlighted. (C-J) RT-qPCR analysis of CXCL2, FTH1, JUN, VEGFA, SLC7A11, SRC, and SLC3A2 mRNA levels in PBMCs from TB patients (n=28) and healthy controls (n=25). (K-L) Fluorescence microscopy images showing Fe²⁺ accumulation (red) in Mtb-infected (MOI=10, 24 h) THP-1-derived macrophages and RAW264.7 macrophages. Images are representative of three independent experiments. Scale bar, 100 μm. (M-N) Flow cytometry quantification of lipid peroxidation (BODIPY 581/591 C11 staining) in infected macrophages with or without ferroptosis inhibitor Ferrostatin-1 (Fer-1, 10 μM). Scale bar, 100 μm. (O) Cell viability assay showing cell death rates in THP-1-derived macrophages. Statistical significance was determined by Student’s t-test or ANOVA (**p* < 0.05, ***p* < 0.01, ****p* < 0.001, *****p* < 0.0001). Data are presented as mean ± SD.
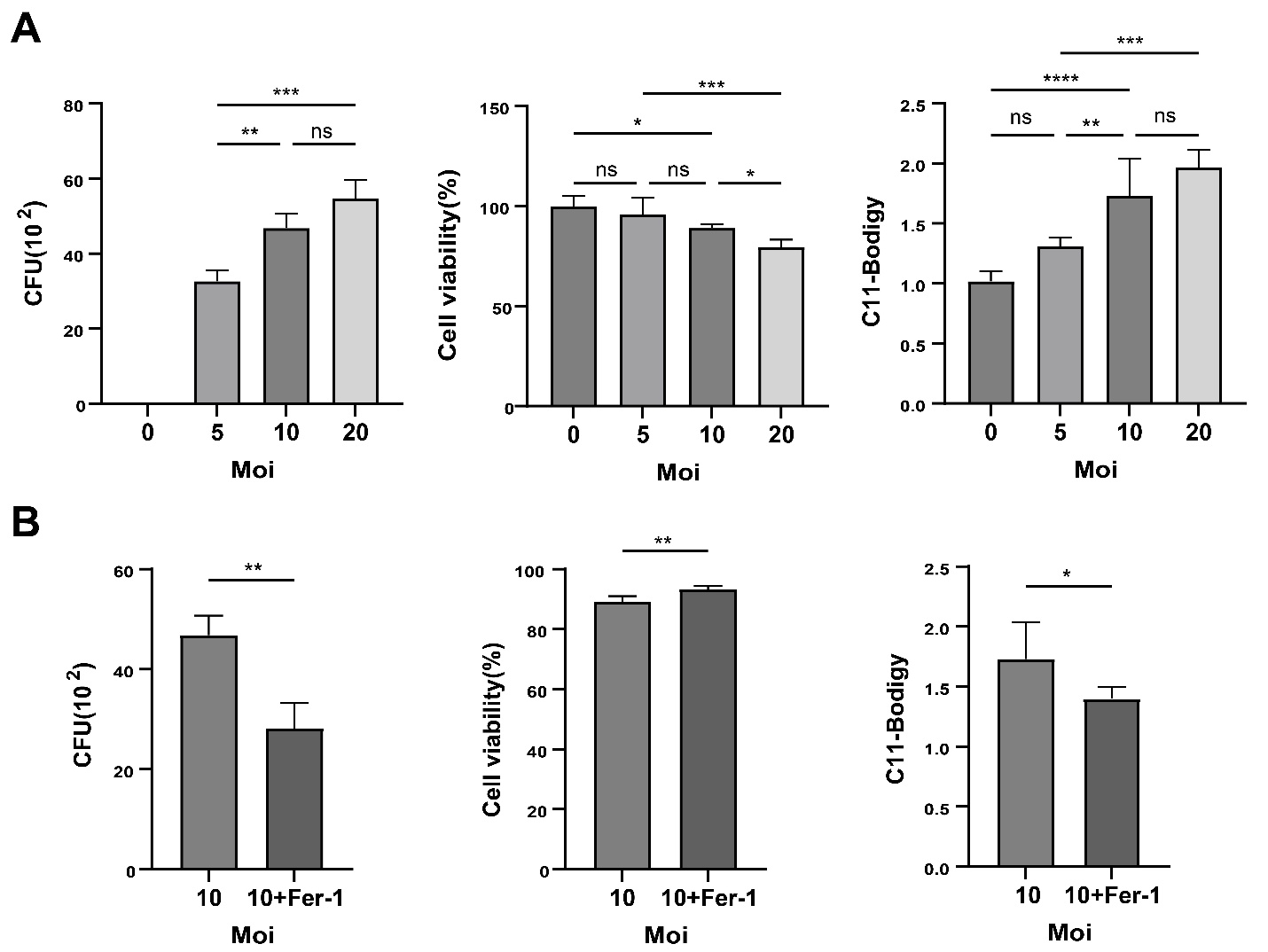


**Supplementary Figure 2. Optimization and validation of the H37Ra infection model for studying ferroptosis in macrophages.**

(A) Determination of the optimal multiplicity of infection (MOI). THP-1-derived macrophages were infected with the attenuated Mtb strain H37Ra at the indicated MOIs for 24 hours. (Left) Intracellular bacterial load assessed by CFU assay. (Middle) Corresponding macrophage viability measured by CCK-8 assay. (Right) Induction of lipid peroxidation (relative C11-BODIPY signal). The MOI of 10 was selected for subsequent experiments as it achieved a high infection burden while maintaining sufficient cell viability and eliciting a strong ferroptosis-associated phenotype. (B) Validation of ferroptosis as the predominant cell death modality at the selected MOI. Macrophages were infected with H37Ra (MOI=10, 24 h) in the presence of the specific ferroptosis inhibitor Fer-1 (10 µM). (Left) Intracellular bacterial load assessed by CFU assay. (Middle) Corresponding macrophage viability measured by CCK-8 assay. (Right) Lipid peroxidation levels measured by flow cytometry using the C11-BODIPY 581/591 probe (green fluorescence shift). Data are presented as mean ± SD. Statistical significance was determined by Student's t-test or ANOVA (**p* < 0.05, ***p* < 0.01, ****p* < 0.001, *****p* < 0.0001).
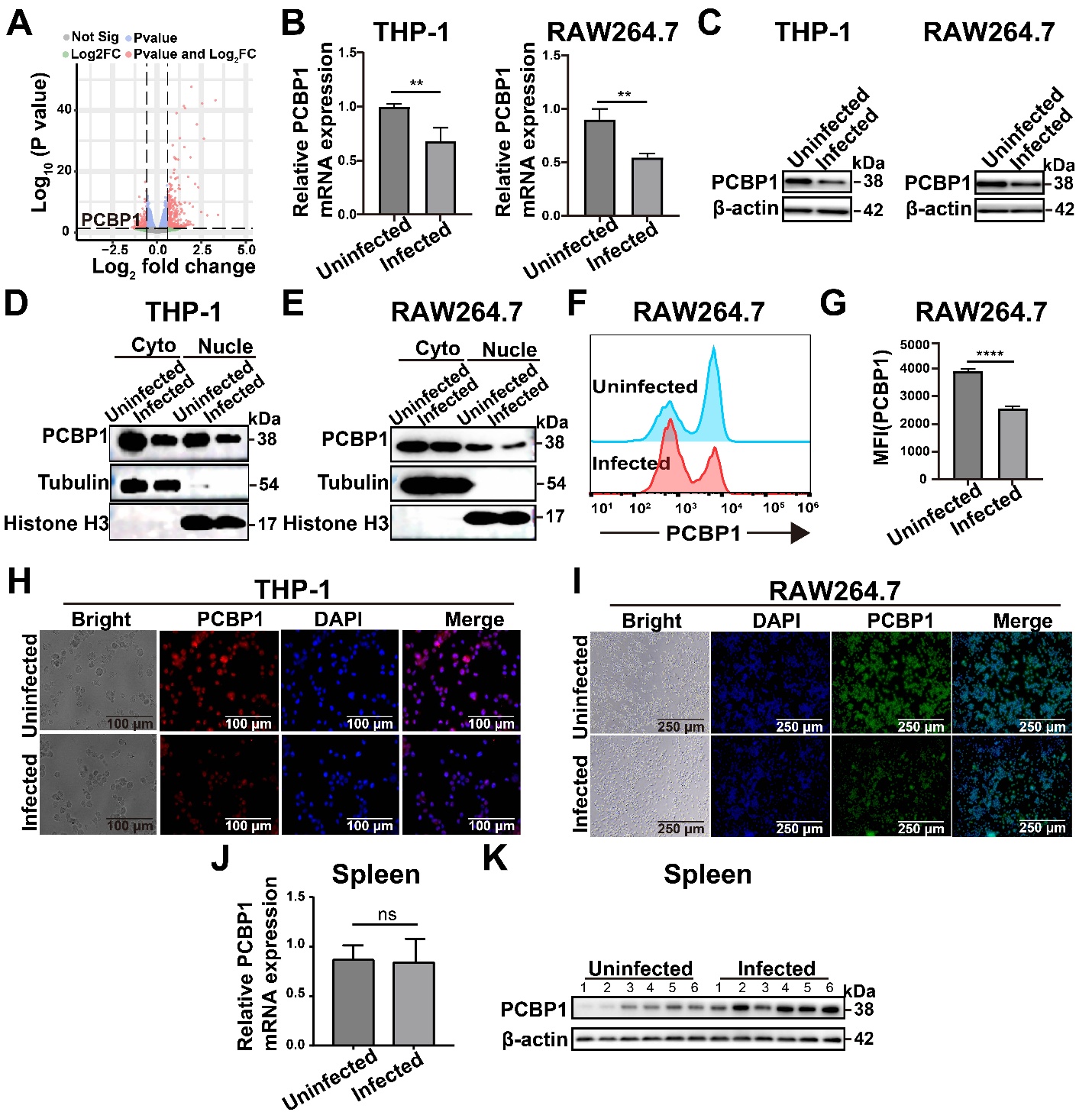


**Supplementary Figure 3. Comprehensive validation of PCBP1 downregulation in Mtb-infected models.**

(A) Analysis of PCBP1 mRNA expression in TB-related samples from the GEO dataset GSE203261. (B-C) RT-qPCR and WB validation of PCBP1 expression in Mtb-infected macrophage models. (D-E) Nuclear-cytoplasmic fractionation assays in Mtb-infected RAW264.7 cells. (F-G) Flow cytometry histograms and MFI quantification of PCBP1 in infected macrophages. (H-I) Fluorescence microscopy images of PCBP1 in Mtb-infected THP-1 macrophages. Scale bar, 100 μm for (K); 250 μm for (I). (J-K) PCBP1 mRNA and protein levels in the spleen tissues of Mtb-infected mice (no significant changes observed). Data are presented as mean ± SD. Statistical significance was determined by Student's t-test (***p* < 0.01, *****p* < 0.0001).
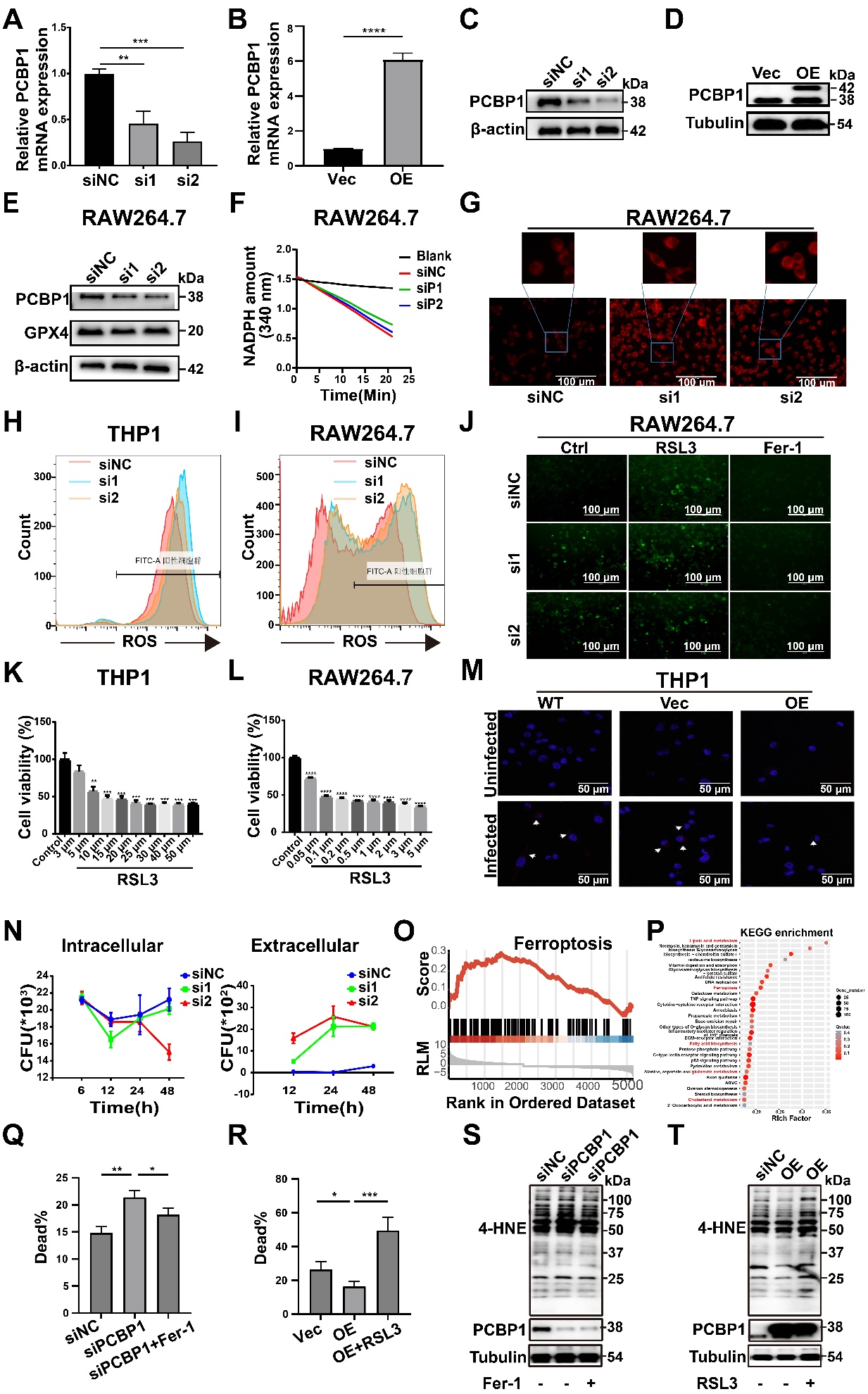


**Supplementary Figure 4. Validation of PCBP1-modulated ferroptosis phenotypes and antibacterial activity in macrophages**

(A-D) Validation of PCBP1 knockdown and overexpression models in THP-1 and RAW264.7, confirming model establishment. (E-F) Evaluation of GPX4 protein expression and enzymatic activity in PCBP1-modulated macrophages, highlighting PCBP1's role in GPX4 regulation. (G) Quantification of intracellular Fe²⁺ levels in PCBP1-altered macrophages, linking PCBP1 to iron homeostasis. Scale bar, 100 μm. (H-I) Assessment of intracellular ROS levels post-PCBP1 knockdown, indicating oxidative stress changes. (J-K) Determination of RSL3 IC50 values in THP-1 and RAW264.7 macrophages, revealing cell line-specific ferroptosis inducer sensitivity. Scale bar, 100 μm. (L) Analysis of lipid peroxidation levels in PCBP1-manipulated macrophages with or without RSL3 treatment, demonstrating PCBP1’s impact on ferroptosis progression. (M) WB and immunofluorescence analysis of 4-HNE and TFR1 protein levels in PCBP1-overexpressing macrophages, underscoring PCBP1's role in mitigating ferroptosis via metabolic regulation. Scale bar, 50 μm. (N) Quantification of intracellular and extracellular bacterial loads in PCBP1-modulated macrophages post-Mtb infection, highlighting PCBP1's influence on bacterial containment. (O-P) RNA sequencing analysis of differentially expressed genes in PCBP1-overexpressing Mtb-infected macrophages, revealing ferroptosis pathway enrichment. (Q-R) Cell death rates quantified by flow cytometry using Ghost Dye™ Red 780 in the indicated genetic and pharmacological conditions post‑infection. (Q) PCBP1‑knockdown and Fer‑1 rescue. (R) PCBP1‑overexpression and RSL3 reversal. (S-T) WB analysis of 4‑HNE, a terminal lipid peroxidation product. (S) Fer‑1 rescues elevated 4‑HNE levels induced by PCBP1 knockdown. (T) RSL3 reverses the suppression of 4‑HNE by PCBP1 overexpression. Statistical significance was determined by Student's t-test or ANOVA (***p* < 0.01, ****p* < 0.001, *****p* < 0.0001)

**
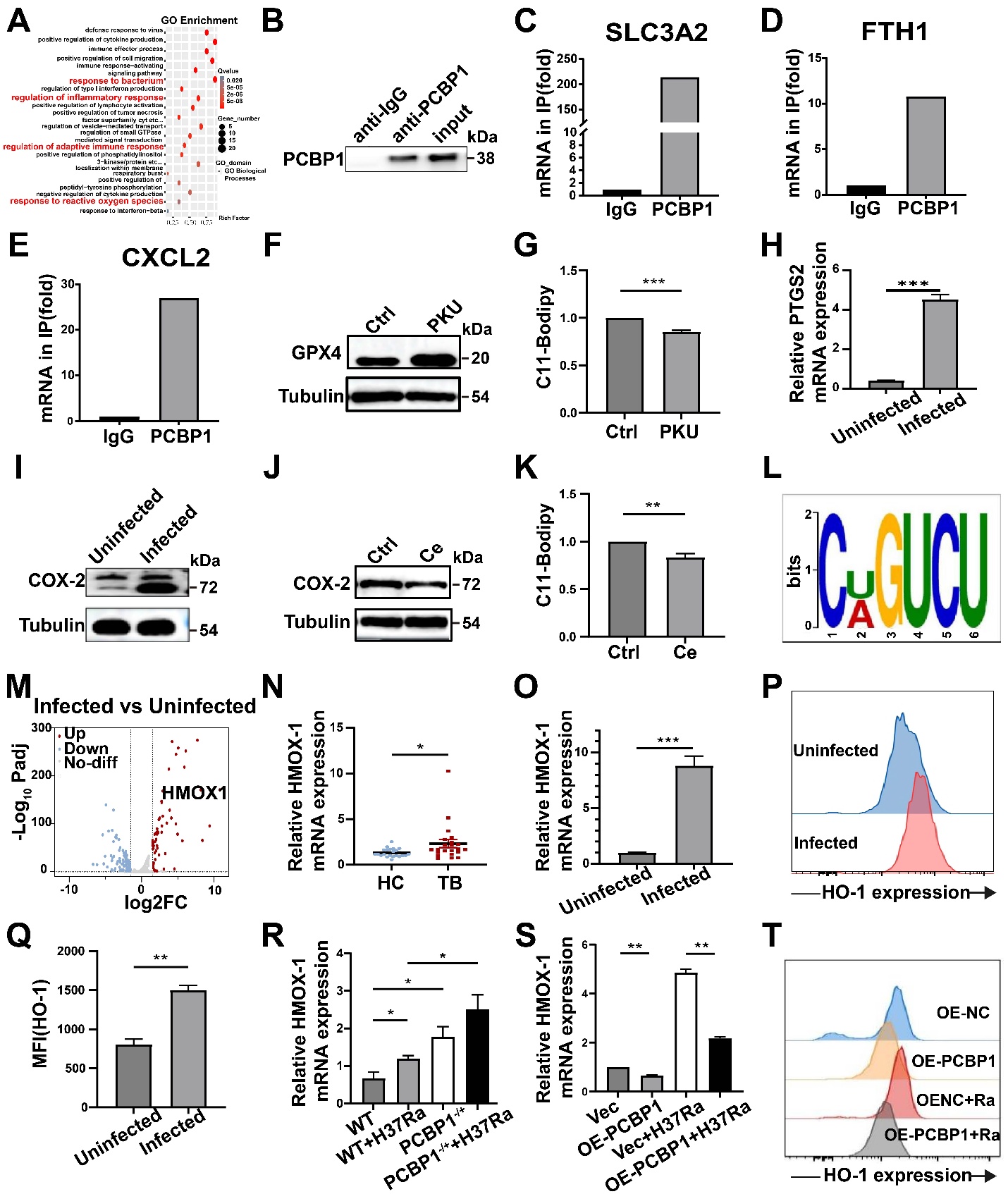
**

**Supplementary Figure 5. PCBP1 Regulates Ferroptosis in Macrophages via Modulating GPX4, PTGS2, and HMOX1.**

1. KEGG pathway analysis of PCBP1-bound mRNAs, highlighting their involvement in ferroptosis-related pathways. (B-E) RIP experiments showing PCBP1 binding to the mRNAs of SLC3A2, FTH1, and CXCL2. (F) Validation of the GPX4-activating effect of PKU. (G) Effects of the GPX4 activator (PKU) on lipid peroxidation levels in Mtb-infected macrophages. (H-I) PTGS2 mRNA and COX-2 protein levels following Mtb infection. (J) Validation of the COX-2 inhibitory effect of Ce. (K) Effects of the COX-2 inhibitor (Ce) on lipid peroxidation levels in Mtb-infected macrophages. (L) Predicted PCBP1 binding motifs in HMOX1 mRNA. (M) HMOX1 mRNA levels from transcriptomic sequencing of Mtb‑infected vs. uninfected macrophages. (N) HMOX1 mRNA levels in PBMCs from pulmonary TB patients (n=23) and healthy controls (n=23). (O) HMOX1 mRNA levels in macrophages at indicated time points post‑H37Ra infection (MOI=10), as determined by RT‑qPCR. (P) Representative flow cytometry histograms showing intracellular HO‑1 protein expression in uninfected and Mtb‑infected (24 h) macrophages. (Q) Quantitative analysis of mean fluorescence intensity (MFI) for HO‑1 protein from the flow cytometry data shown in (P). (R-T) Inverse correlation between PCBP1 and HO‑1 expression. (R-S) HMOX1 mRNA levels in PCBP1‑modulated cells. (T) HO‑1 protein levels by FCM in PCBP1‑overexpressing macrophages. Data are presented as mean ± SD. Statistical significance was determined by Student's t-test or ANOVA (**p* < 0.05, ***p* < 0.01, ****p* < 0.001).
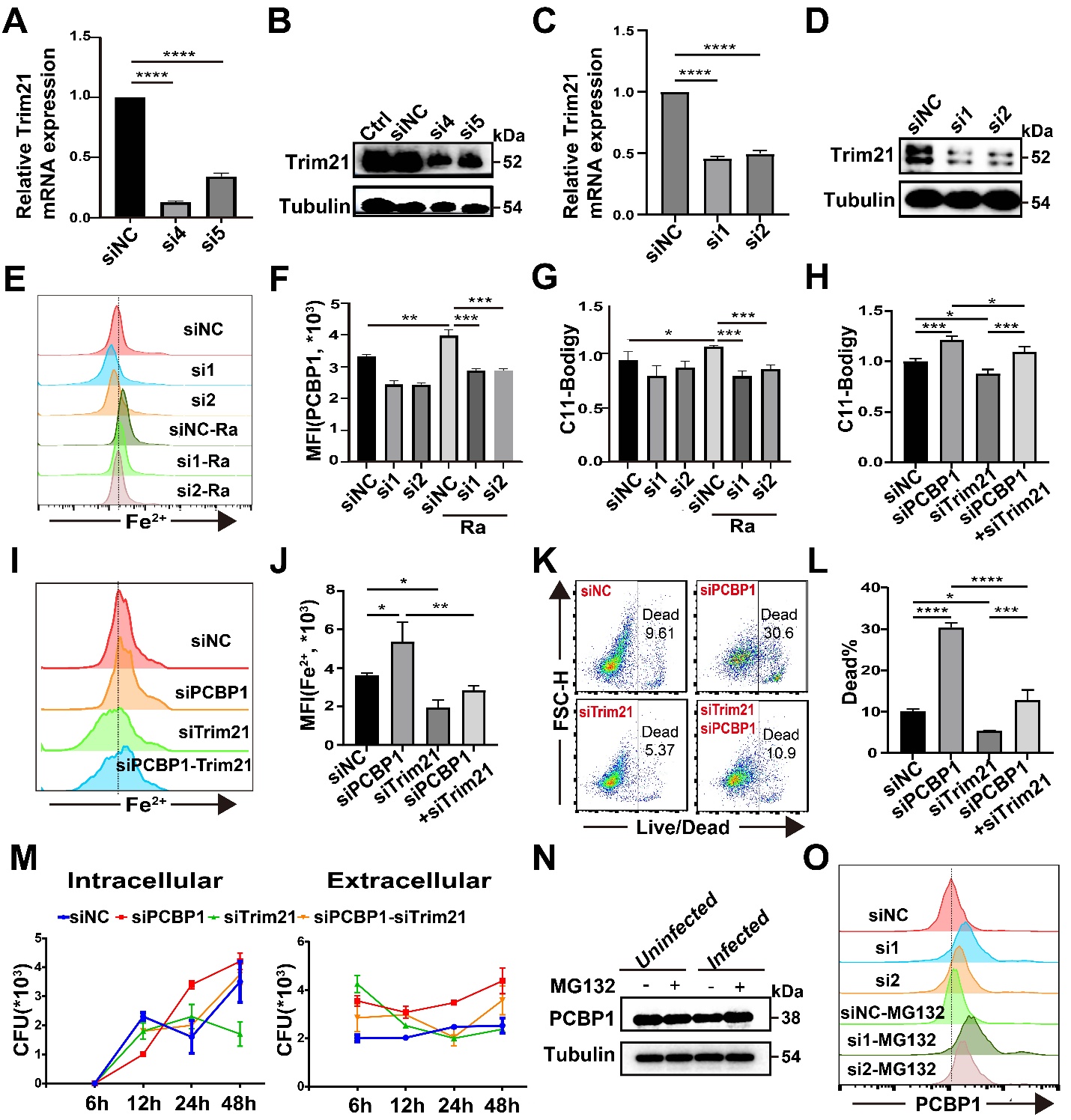


**Supplementary Figure 6. Impact of Trim21 on PCBP1-Mediated Ferroptosis in Mtb-Infected Macrophages**

1. D) Validation of Trim21 knockdown efficiency in THP-1 and RAW264.7 macrophages using siRNA. (E-F) Intracellular Fe²⁺ levels in Trim21-knockdown Mtb-infected macrophages. (G) Lipid peroxidation levels in Trim21-knockdown Mtb-infected RAW264.7 macrophages. (H) Lipid peroxidation levels in PCBP1-knockdown macrophages with concurrent Trim21 knockdown. (I-J) Fe²⁺ levels in PCBP1-knockdown macrophages with Trim21 knockdown. (K-L) Macrophage cell death rates in PCBP1-knockdown macrophages with Trim21 knockdown. (M) Analysis of intracellular and extracellular Mtb burden in PCBP1-knockdown macrophages with Trim21 knockdown. (N) WB detection of PCBP1 protein levels in RAW264.7 macrophages under conditions of Mtb infection or non-infection, and MG132 treatment or no treatment. (O) Flow cytometry analysis of PCBP1 protein expression in RAW264.7 macrophages with or without Trim21 knockdown and with or without MG132 treatment. Data are presented as mean ± SD. Statistical significance was determined by Student's t-test or ANOVA (**p* < 0.05, ***p* < 0.01, ****p* < 0.001, ****p* < 0.0001).
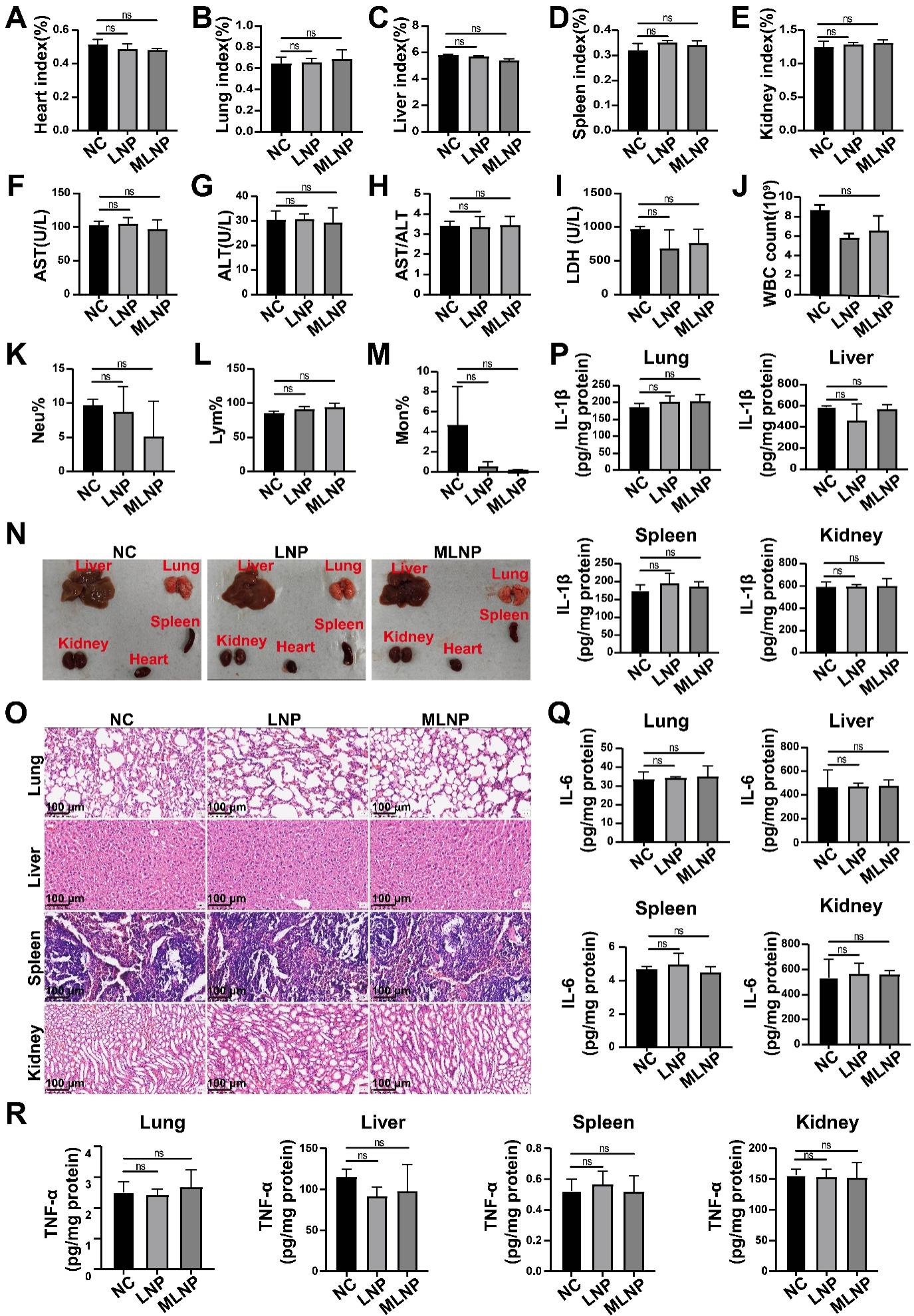


**Supplementary Figure 7. Comprehensive safety and biocompatibility assessment of empty LNP/MLNP nanoparticles in healthy mice.**

(A–E) Major organ‑to‑body weight ratios (heart, lung, liver, spleen, and kidney) of mice treated with sterile enzyme‑free water (Control), empty LNPs, or empty MLNPs. (F–I) Serum levels of liver function markers: AST (F), ALT (G), AST/ALT(H), and LDH (I). (J–M) Routine blood analysis showing white blood cell count (WBC, J), neutrophil percentage (Neu%, K), lymphocyte percentage (Lym%, L), and monocyte percentage (Mon%, M). (N–O) Representative macroscopic photographs (N) and H&E‑stained sections (O) of major organs (lung, liver, spleen, kidney) from treated mice, demonstrating no evident morphological or histopathological damage. Scale bar, 100 μm. (P–R) Levels of key pro‑inflammatory cytokines (IL‑1β, IL‑6, and TNF‑α) in tissue homogenates of the lung , liver , spleen and kidney from mice in the indicated treatment groups, as determined by ELISA. Statistical significance was determined by ANOVA (ns, not significant).

**
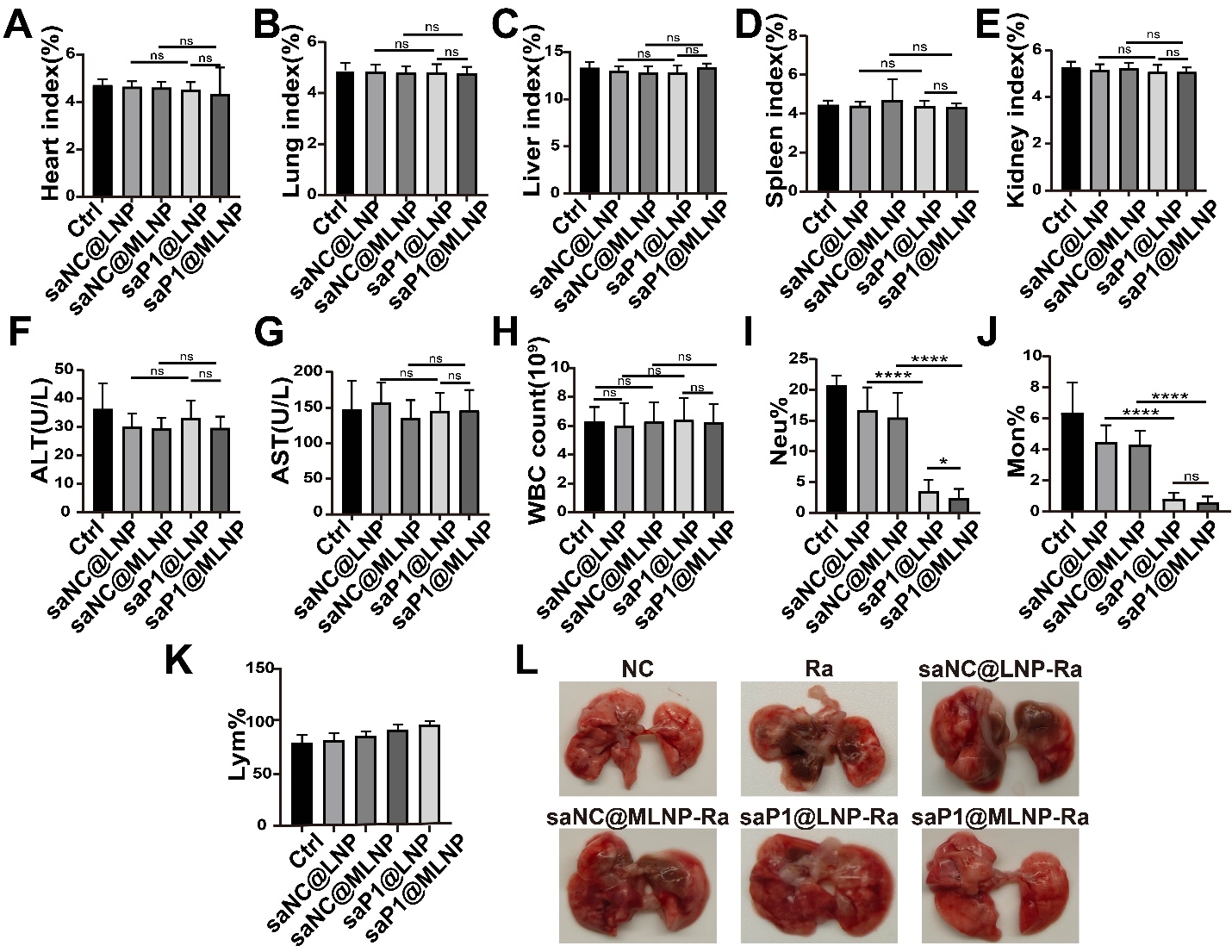
Supplementary Figure 8. Safety profile and immunological effects of PCBP1‑activating nanoparticles in Mtb‑infected mice.**

(A–E) Organ‑to‑body weight ratios of the heart (A), lung (B), liver (C), spleen (D), and kidney (E) in the indicated treatment groups at the experimental endpoint. (F–G) Serum levels of liver function markers AST (F) and ALT (G). No significant differences were observed compared to the non‑specific control groups (saNC@LNP and saNC@MLNP). (H–K) Peripheral blood cell analysis. (H) Total WBC counts. (I, J) Percentages of neutrophils (Neu%) and monocytes (Mon%). (K) Percentage of Lym%. (L) Representative macroscopic photographs of lungs from the indicated treatment groups, showing a reduction in gross pathological lesions following saPCBP1@MLNP therapy. Statistical significance was determined by ANOVA **p* < 0.05, *****p* < 0.0001.

**
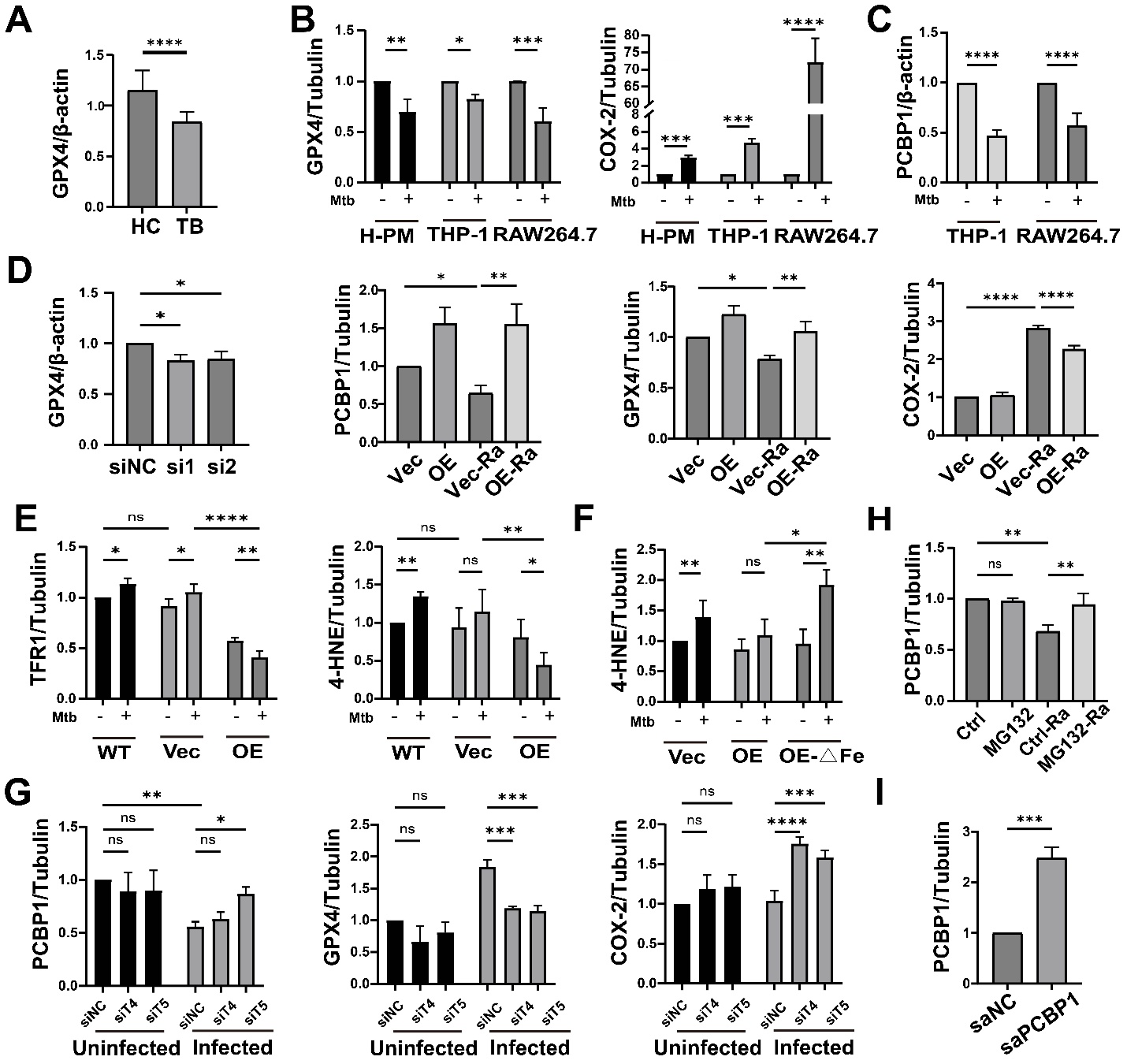
Supplementary Figure 9. Semi-quantitative densitometric analysis of key Western blot bands shown in the main figures.**

Densitometric quantification of protein expression levels normalized to the corresponding loading control (Tubulin, GAPDH, or β‑actin). Data are presented as mean ± SD from at least three independent experiments. Statistical significance was determined by Student's t‑test, one‑way ANOVA, or two‑way ANOVA (*p < 0.05, **p < 0.01, ***p < 0.001, ****p < 0.0001). Each bar graph corresponds to the following main figure panel:

(A) Figure 1D – GPX4 protein expression in PBMCs from TB patients (n=6) and healthy controls (n=6). (B) Figure 1F – GPX4 and COX‑2 protein levels in Mtb‑infected human primary macrophages, THP‑1‑derived macrophages, and RAW264.7 macrophages. (C) Figure 2H & Supplementary Figure 3C – PCBP1 protein expression in Mtb‑infected macrophages (THP‑1, RAW264.7). (D) Figure 3A – GPX4 and COX‑2 expression after PCBP1 knockdown (siPCBP1) or overexpression (OE‑PCBP1) in THP‑1 macrophages. (E) Figure 3F – 4‑HNE and TFR1 protein levels in PCBP1‑overexpressing macrophages. (F) Figure 4D – 4‑HNE expression in macrophages overexpressing PCBP1 versus the Fe‑binding domain mutant (ΔFe). (G) Figure 6I – PCBP1, GPX4 and COX‑2 expression after Trim21 knockdown (siTrim21) in Mtb‑infected macrophages. (H) Figure 6Q – PCBP1 protein levels in THP‑1‑derived macrophages under the indicated conditions (Mtb infection ± MG132 treatment). (I) Figure 7I – PCBP1 expression in RAW264.7 macrophages transfected with saPCBP1‑loaded MLNPs.
